# Development of a Smoke-Free Homes Intervention for Parents: An Intervention Mapping Approach

**DOI:** 10.5334/hpb.20

**Published:** 2019-12-19

**Authors:** Rachel O’Donnell, Ruaraidh Dobson, Marijn de Bruin, Stephen Turner, Lorna Booth, Sean Semple

**Affiliations:** * University of Stirling, GB; † University of Aberdeen, GB; ‡ Glasgow Caledonian University, GB

**Keywords:** Intervention mapping, second-hand smoke, smoke-free home, air quality monitoring, children

## Abstract

Exposure to second-hand smoke (SHS) is associated with various ill-health outcomes for children and adults. Barriers to creating a smoke-free home (SFH) are well-documented. Feasible and effective interventions to create smoke-free homes for disadvantaged households are lacking. Interventions that include providing parents with objective information about the impact of smoking on air quality in their home may be particularly effective. This study describes the development of a novel, theory- and evidence-based smoke-free homes intervention using objectively-assessed air quality feedback. The intervention was developed using the six-step Intervention Mapping (IM) protocol. Findings from literature reviews, focus groups with parents, interviews with health/care professionals, and expert panel discussions shaped intervention content and materials. Findings highlighted the importance of parents receiving personalised information on second-hand smoke levels in their home. Professionals considered the use of non-judgemental language essential in developed materials. Previous literature highlighted the need to address home smoking behaviour at a household rather than individual level. The AFRESH intervention is modular and designed to be delivered face-to-face by healthcare professionals. It includes up to five meetings with parents, two sets of five days’ air quality monitoring and personalised feedback, and the option to involve other household members in creating a smoke-free home using educational, motivational, and goal setting techniques. Further research is needed to evaluate the acceptability and effectiveness of the AFRESH intervention and which specific groups of parents this intervention will most likely benefit. IM was a useful framework for developing this complex intervention. This paper does not present evaluation findings.

## Introduction

Exposure to second-hand smoke is associated with a wide-range of preventable, adverse health outcomes in infants, children and adults. Inhaling second-hand smoke can lead to acute irritant and chronic inflammatory effects on the respiratory system, and is likely to be especially harmful during early life.

Fifteen percent of children living in the most deprived areas of Scotland are exposed to second-hand smoke in their homes, compared to close to zero percent of children living in the least deprived communities (Scottish Government 2016). Alongside this widening inequality in exposure, children in poorer communities in countries where smoke-free laws are partial or poorly enforced have seen almost no improvement in exposure levels. More than eighty-five percent of second-hand smoke is invisible (Gee et al. 2013), and recent work has shown that second-hand smoke remains in household air for up to five hours after a cigarette is extinguished (Semple & Latif 2014). Many smokers remain unaware of the impact of their smoking on air-quality in their home (Wilson et al. 2013a). Most smokers try to protect their children from second-hand smoke, often applying strategies that reduce rather than eliminate second-hand smoke risks completely, such as smoking at the kitchen window, or only smoking in the house when children are absent (Wilson et al. 2013a; Wilson et al. 2013b).

A recent systematic review and meta-analysis (Rosen et al. 2015) of interventions designed to reduce household second-hand smoke exposure identified seven interventions that had used objective measures of household air quality as an outcome measure. The meta-analysis demonstrated that these approaches improved air concentrations of fine particulate matter (PM_2.5_) or nicotine within the home. However, all studies reported evidence of continued second-hand smoke exposure to some degree post-intervention. More recently, a Cochrane review (Behbod et al. 2018) of interventions designed to reduce children’s exposure to second-hand smoke in the home screened 78 relevant studies – only 24 of which reported a statistically significant intervention effect for reducing children’s second-hand smoke exposure levels. Of these 24 studies, 13 used objective measures of children’s second-hand smoke exposure, and the authors were unable to pinpoint what made these interventions effective. None of these studies used objective measures of household air quality (PM_2.5_) as an outcome measure.

Several studies have explored the use of air quality feedback as a means of changing smoking behaviour in the home, and there is mixed evidence on the effectiveness of interventions using this approach in disadvantaged households. The REFRESH intervention showed considerable promise in a feasibility study (Wilson et al. 2013b) using face-to-face discussions between parents and health workers on why/how to make the home smoke-free. An air quality monitor was installed in each home, and parents received personalised air quality feedback to enhance their awareness of the impact of their smoking behaviour. Key barriers identified were instrumentation cost and the labour-intensive method of intervention delivery, which involved researchers conducting home visits to install and retrieve air quality monitoring devices. The intervention had developed organically based on experience developed over the years, and did not make full use of existing theories and evidence on behaviour change.

Based on REFRESH, a recent randomised controlled trial (Semple et al. 2018) examined whether delivery of personalised air quality feedback plus standard advice on the health effects of second-hand smoke was more effective than standard advice alone in helping disadvantaged mothers protect their children from second-hand smoke. The intervention was embedded within the National Health Service (NHS) Lanarkshire First Steps (FS) early intervention programme, overcoming the labour-intensive delivery methods used in REFRESH. Neither standard advice nor standard advice plus air quality feedback were effective in reducing PM_2.5_ concentrations. This may reflect the intervention targeting young mothers, despite the fact that in many households, other adults (partners, parents and visitors) also smoked in the home. The authors conclude that future work should consider ways to engage with all adults in the home to achieve sustained household behaviour change in relation to smoking. Another recent randomised controlled trial (Ratschen et al. 2018) tested a complex intervention based on other feasibility work (Marsh et al. 2016), providing families with personalised feedback on home air quality, behavioural support and nicotine replacement therapy for temporary abstinence. This approach was effective in significantly reducing children’s exposure to second-hand smoke in the home, and most participants ranked the personalised air quality feedback as the single most important intervention component.

Interventions using personalised air quality feedback to reduce children’s exposure to second-hand smoke have found mixed evidence of effectiveness. Recent work has highlighted the complex interplay that exists between barriers, motivators and enablers to creating a smoke-free home in many households, which can make this a difficult achievement (Rowa-Dewar, Lumsdaine & Amos 2015; Passey et al. 2016). However, developing effective interventions that enable parents to create a smoke-free home is one of the key ways that children’s exposure to second-hand smoke can be reduced globally. A recent call (Conner & Norman 2017) has been made for a greater focus on developing and testing theory-based, rather than “theory inspired” (Michie & Abraham 2004) interventions to advance research on effective health behaviour change. Smoke-free home interventions have yet to make optimal use of existing behaviour change theories. Our study aimed to develop a theory-based intervention programme to reduce second-hand smoke levels in the home (AFRESH), utilising personalised air quality feedback. To address an identified gap in previous research in this area, the intervention was developed for health/care professionals (e.g. family nurses, early years’ workers) to use with parents who wish to move towards having a smoke-free home, and other household members who smoke in the home.

### Intervention Mapping

**Figure 1:**
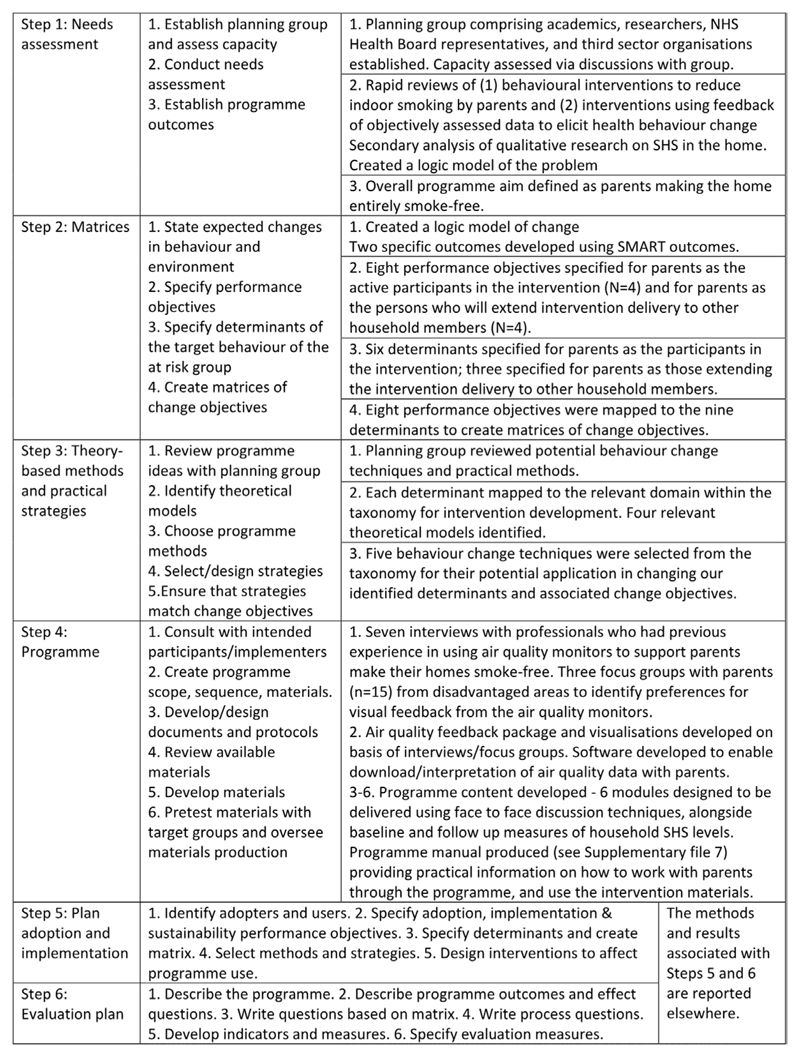
Intervention mapping steps and tasks.

Intervention Mapping (IM) is a widely-used framework for developing (complex) behaviour change interventions based on theory, empirical evidence, and input from key stakeholders (Bartholomew et al. 2016). This framework was chosen to develop AFRESH as it details the methods that are suitable for changing important behavioural determinants such as knowledge, attitude and self-efficacy, and how these methods should be applied for optimal effectiveness. IM ensures all components in the programme development process are transparent, linked, and supported by a clear rationale for each choice based on theory, evidence, and/or expert opinion. For the replicability and future synthesis of interventions, it is important that detailed descriptions of the interventions and the process of their development are published (Bartholomew et al. 2016; Schaalma & Kok 2009). IM comprises of six steps, each with several specific tasks (see Figure 1). This manuscript reports on the development of AFRESH using Steps 1 to 4. Steps 5 and 6 focus on implementation and evaluation plans. The results of piloting AFRESH are reported elsewhere (Dobson et al. 2017).

## Methods and Results

### Step 1: Needs Assessment

#### Method

A planning group was established with academics and researchers involved in previous second-hand smoke-related research, NHS Board representatives, and relevant non-profit organisations. A member of our research team spoke informally with each group member about their prior experiences of delivering air quality interventions. These discussions informed the development of topic guides and interview schedules used in Stage 4 (see Supplementary file 1 – Interview schedule for use with health and care professionals, and Supplementary file 2 – Parent focus group topic guide).

We also conducted 1) a rapid review of the literature on behavioural interventions to reduce indoor smoking by parents; 2) a rapid review of interventions that use feedback of objectively assessed data to elicit health behaviour change; and 3) a secondary analysis of previous qualitative research regarding reasons for reducing second-hand smoke in the home. These sources of information were utilised not only in the needs assessment, but also in later steps in the intervention development process.

During Step 1, the following literature reviews were developed:

- AFRESH review of behavioural interventions to reduce indoor smoking by parents (2016) (see Supplementary file 3)
- AFRESH review of successful interventions that have used objectively assessed feedback to motivate health behaviour change (2016) (see Supplementary file 4)
- AFRESH secondary analysis of qualitative data: second-hand smoke exposure in the home (2016) (see Supplementary file 5)
- Details of the literature search strategies are provided in Supplementary file 6.

#### Results

##### Literature reviews: reasons for smoking indoors

**Figure 2:**
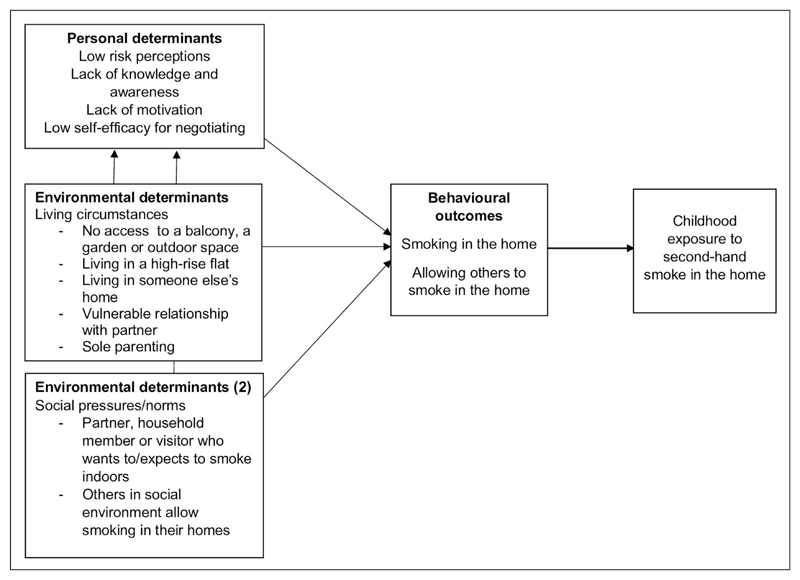
Logic model of the problem.

Smoking restrictions in the home are shaped by a range of sociocultural influences and other factors that create enablers, and barriers for future public health initiatives. The reviews identified that people do not automatically make the connection between the (extent of) risks of second-hand smoke and their smoking behaviour in the home (Rowa-Dewar, Lumsdaine & Amos 2015). However, most parents state that they are aware that children are more vulnerable, and that they want to protect their children’s health (Wilson 2013a). Living circumstances seem to have a major impact on the reduction of second-hand smoke in the home; for example, living in a block of flats and being unable to leave the children alone to go outside to smoke. Qualitative review findings also suggested that parent/carers’ level of knowledge, awareness and risk perception are themes that should be considered when developing future second-hand smoke reduction intervention studies, which should also encourage positive social norms (good parenting), tackle negative social norms and address negative feelings of stigmatisation (Passey et al. 2016; Phillips et al. 2007; Rowa-Dewar, Lumsdaine & Amos 2015; Wilson et al. 2013a). The key factors identified as reasons for smoking indoors during the Step 1 needs assessment work are displayed in a logic model of the problem (see Figure 2).

Given that the World Health Organisation recognises there is no safe level of exposure to second-hand smoke in the home (WHO Framework Convention Alliance for Tobacco Control, 2005), the research team agreed that the overall programme aim is to have parents make the home entirely smoke-free. Our previous work has identified that health/care professionals are the desired delivery personnel for this type intervention. This was also the finding of our review of second-hand smoke interventions, which highlighted that successful feedback interventions involved face-to-face communications with health/care professionals (Bovet et al. 2002; De Blok et al. 2006). Incorporating objectively assessed data and motivational interviewing (MI) appear to be the most popular adopted intervention methods and the most effective for second-hand smoke reduction with parents and caregivers of young children (Baheiraei et al. 2011; Emmons et al. 2001; Harutyunyan et al. 2013; Wilson et al. 2013b).

### IM Step 2: Creating matrices of change

#### Method

**Figure 3:**
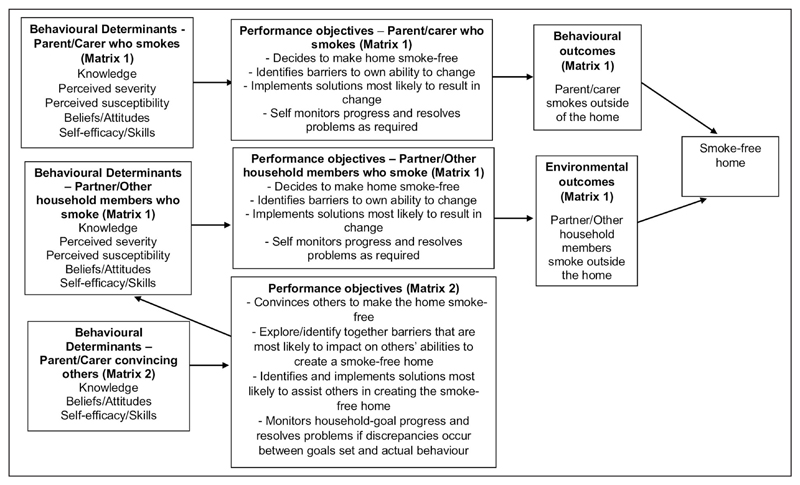
Logic model of change.

The tasks in step 2 (see Figure 1) were completed through discussion, consulting the literature and behaviour change theory. Two specific outcomes for the behaviour of the target group were developed using SMART (specific, measurable, achievable, realistic and timely) outcomes. Outcome 1 was developed using published literature on the outcomes of smoke-free homes interventions as a guide, and specifies that within three months of intervention initiation, 40% of participating parents no longer smoke indoors. Previous studies have suggested that the smoking behaviours of other household members should not be overlooked in any second-hand smoking intervention, as a ‘household approach’ to creating smoke-free homes may be more effective than an individualised approach in households where more than one adult smokes (Brown et al. 2015; Semple et al. 2018). On this basis and given the lack of published smoke-free homes research that includes partners/other household members as active participants, ‘participating parents’ (Outcome 1) also includes partners and other adult household members who smoke. Outcome 2 was developed through research team discussions, and states that within three months of intervention initiation, parents/carers engaged in the intervention will have engaged with as many partners/other household members who smoke as possible, to convince them to no longer smoke indoors. A logic model of change was created to summarise the desired outcomes of the intervention (see Figure 3).

Our review findings demonstrated that central to our behaviour change intervention is parents’ intrinsic motivation to protect their children and be considered by others as a good parent (Wilson et al. 2013a; Wilson et al. 2013b) and the importance of behavioural feedback and self-monitoring in self-regulating behaviour (de Bruin et al. 2012), provided here through the air quality monitor. Self-determination Theory (SDT) (Deci & Ryan 1985) and Control Theory (Carver & Scheier 1982; Carver & Scheier, 1998) were therefore selected as the key theories to guide the development of our intervention. The outputs of Step 2 were placed in matrices of change objectives, which detail exactly what changes need to be accomplished to achieve the overall desired outcome of the intervention – the creation of smoke-free homes, and the SMART outcomes outlined above.

#### Results

Two matrices are provided in Tables 1 and 2. The first applies to parents (mothers, fathers or step-parents) who are the participants in the intervention, and the partners/other household members who they persuade to create a smoke-free home. The second matrix outlines the steps required for parents to effectively extend intervention delivery to other household members who smoke. Although Matrix 2 could also be considered as part of intervention implementation (Step 5), it directly addresses issues identified in the needs assessment and is therefore included here. Performance objectives, which are sub-behaviours or actions that should result in the final health behaviour of making the home smoke free are given in column 1 of Tables 1 and 2. In the top column, the most important and changeable determinants of behaviour change are given. Wherever the behavioural determinants were judged to be relevant for that particular behaviour, a change objective was formulated.

Each of the performance objectives, determinants and change objectives were derived from existing literature, our review work, interviews, and expert input, in combination with SDT and Control Theory as our main theories. For example, the performance objective ‘Decide to make home smoke-free’ (see Table 1) reflects the setting of a goal following a motivational process (Carver & Scheier 1982; Carver & Scheier, 1988). For this performance objective, the determinants knowledge, risk perception (susceptibility and severity), beliefs/attitudes and self-efficacy/skills were identified to be important, based on previous literature (Passey et al. 2016; Phillips et al. 2007; Rowa-Dewar, Lumsdaine & Amos 2015; Wilson et al. 2013a). Associated change objectives formulated through team discussion were ‘Explain the health risks associated with childhood second-hand smoke exposure in the home’, ‘Explain that there is no safe level of exposure to second-hand smoke’ and ‘Recall the strategies that are ineffective in removing risk completely’. The performance objective ‘Self-monitor goal progress and respond by resolving problems if discrepancies occur between the goals set and actual behaviour’ (see Table 1) was also derived from control theory, i.e. that people have a goal in mind and try to move towards it, are more successful in achieving their goals if they monitor their progress towards this goal, and take steps to reduce the discrepancy between their goal and actual behaviour as required (Carver & Scheier 1982; Carver & Scheier 1998).

SDT is particularly focused on the ways in which an individual acquires the motivation for initiating behaviour change, and maintaining new behaviours over time. The theory argues that developing a sense of autonomy, competence and relatedness are critical to the process of ‘internalisation’ – the process by which behaviours become more autonomously regulated, or valued, over time. According to SDT, the least internalised form of regulation is ‘*external*’, and reflects engaging in behaviours to gain some reward or recognition (for example, an individual creating a smoke-free home in order to receive a financial incentive, or because a health care practitioner pressures them to do so). In such instances, long-term health behaviour change is unlikely (Ng et al. 2012). The most internalised form of regulation is ‘*integrated*’, and reflects engaging in behaviours because they are consistent with an individual’s own goals and values (for example, creating a smoke-free home because an individual values the health of their children, and because this behaviour is consistent with other goals in their life such as reducing cigarette consumption) (Patrick & Williams 2012). Engaging in health behaviours for more autonomous reasons is likely to result in better behavioural adoption and maintenance (Deci & Ryan 2008). Previous research has also suggested that combining MI approaches with the theoretical approach of SDT may elicit greater behaviour change (i.e. Deci & Ryan 2012; Patrick & Williams 2012). MI can be used to put the theoretical foundation of SDT – attainment of competence, autonomy and relatedness – into practice (Vansteenkiste et al. 2012).

We used the principles of SDT to seek to strengthen parents/carers’ personal motivation for and commitment to creating a smoke-free home by eliciting and exploring individual reasons for change. The rationale is thus that if the intervention is successful in increasing autonomous behaviours (the extent to which behaviours originate from the self), parents/carers will be more likely to decide to create and maintain their smoke-free home. On this basis, SDT was used to develop the performance objectives ‘Explore and identify barriers that are most likely to impact on their own ability to create a smoke-free home’ and ‘Identify and implement solutions that are most likely to assist them in creating a smoke-free home’, which support autonomy through eliciting and acknowledging individuals’ perspectives regarding their own barriers and solutions, whilst minimising control and avoiding judgement (Patrick & Williams 2012). Other components of the intervention fit well with SDT, for example the change objectives ‘Recognise that people are more successful if they see ‘failure’ as a learning opportunity’ and ‘Recognise that most parents in the same situation are struggling on occasions’ (associated with the ‘Self-monitor goal progress…’ performance objective), as they provide support for competence (reframing past failures as short successes and being positive that individuals can succeed) and relatedness (providing unconditional positive regard, particularly in the face of failure to achieve desired goals) (Patrick & Williams 2012).

### IM Step 3: Theoretical methods and practical strategies

#### Method

The tasks in Step 3 (see Figure 1) were completed using Bartholomew et al.’s (1998) taxonomy for intervention development, published by Kok et al. (2016) and extracted from Bartholomew et al.’s (2011) Intervention Mapping protocol. This taxonomy details the methods that are suitable for changing important behavioural determinants (such as knowledge, attitude, or self-efficacy), and how these methods should be applied for optimal effectiveness. Given our focus on translating intentions into behaviour, we identified behaviour change methods from the trans-theoretical model, goal setting theory, and theories of self-regulation to underpin the intervention, in conjunction with theories of information processing. Our main behaviour change intervention methods were discussion, elaboration, individualisation, goal setting and planning coping responses. These decisions were supported by our literature review work. For example, planning coping responses is listed in the taxonomy as a suitable method to change self-efficacy and overcome barriers. This requires prompting participants to list potential barriers and ways to overcome these. It also involves identification of high-risk situations and practice of coping responses. The use of this method is also supported by research by Passey et al. (2016), who recommend that professionals should be trained to develop skills in advising on smoke-free home-related practical strategies, for example, how to overcome weather-related barriers, to support households in creating a smoke-free home. The methods selected also linked back to Control Theory. For example, to change social norms and support, individuals are encouraged to develop effective solutions for dealing with social pressure that may hinder change, and to increase self-efficacy/skills, individuals are prompted to list potential barriers and overcome them. The discussion and elaboration methods used to increase knowledge and change beliefs/attitudes drew on MI techniques and SDT, designed to strengthen personal motivation for and commitment to creating a smoke-free home by exploring the parent/carer’s own reasons for change with a compassionate approach.

#### Results

Table 3 shows examples of our determinants, theoretical methods, and their parameters for use and practical applications. Each behaviour change method was considered for its potential application in changing our identified determinants and the change objectives associated with them. These matrices also enabled us to consider what an intervention might look like if we incorporated each specific behaviour change method. A full list of the behaviour change methods selected for each determinant and associated change objectives is presented in Table 4.

For example (see Table 3), for the determinant ‘Knowledge’ and the change objective ‘Explain the health risks associated with childhood second-hand smoke exposure in the home’, we selected the ‘Discussion’ method, which encourages consideration of a topic in an open, informal debate. Its parameters involve listening to the learner to ensure that the correct schemas are activated. Practical application of this method in our intervention therefore involved an informal discussion of the health risks, barriers and solutions for creating a smoke-free home.

### IM Step 4: Programme development

#### Method

The tasks in Step 4 were completed benefiting from previously developed materials in REFRESH (Wilson et al. 2013b) and the intervention literature review. To assist in developing intervention materials, including the specifics of the personalised air quality feedback, seven semi-structured interviews were conducted with health and care professionals who had previous experience in using air quality monitors to support parents making their homes smoke-free. The purpose of the interviews was to elicit their views on the practicalities, challenges and benefits using air quality monitors with parents; the best ways in which to deliver smoke-free homes interventions, the impacts of current visual feedback tools and whether they could be improved, and preferred delivery mode for future smoke-free homes interventions. Detailed notes were taken during each interview by the interviewer. These notes were then written up and key themes were identified and agreed across interviews by two members of the team.

Three focus groups were conducted with parents (n = 15) from disadvantaged urban areas, between March–April 2016, to identify preferences for visual feedback from the air quality monitors. Parents were recruited via gatekeepers from two community groups that work with families in disadvantaged areas to promote positive health and wellbeing. Gatekeepers identified potential participants, distributed invitation letters and information sheets to them and liaised with the researcher to set up suitable focus group times and dates. All focus groups took place within the community centres during usual group meeting times. Written consent was obtained from each participant before the start of each focus group. All parents who took part either smoked, or lived with someone who smoked, and had one or more children under the age of five.

**Figure 4:**
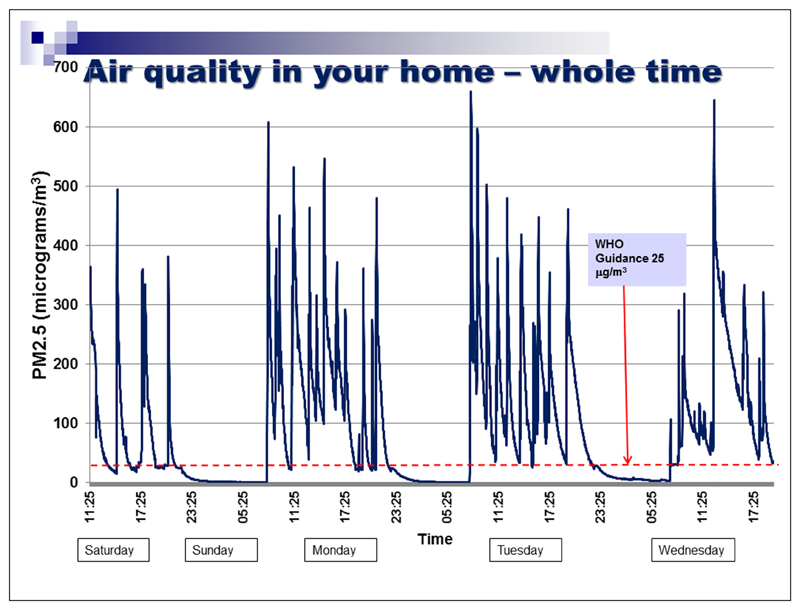
Examples of current feedback package at the time of the needs analysis.

**Figure 5:**
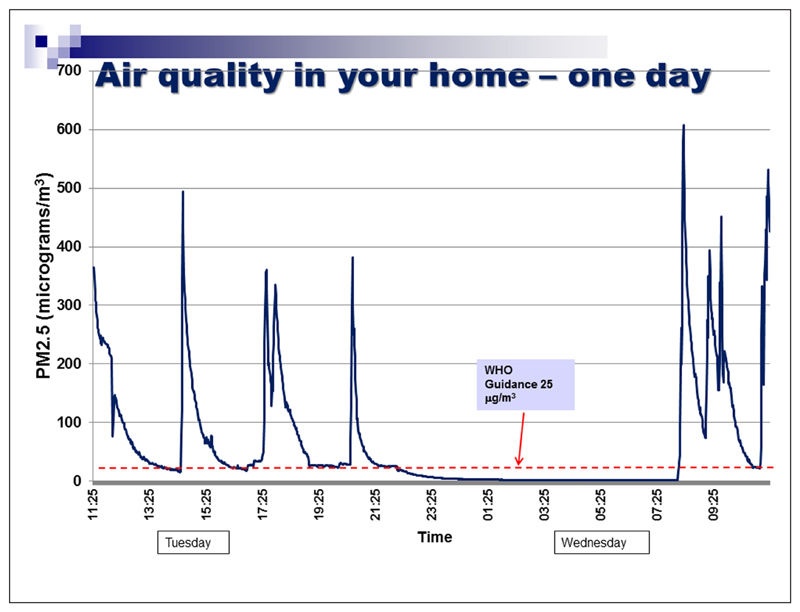
Examples of current feedback package at the time of the needs analysis.

**Figure 6:**
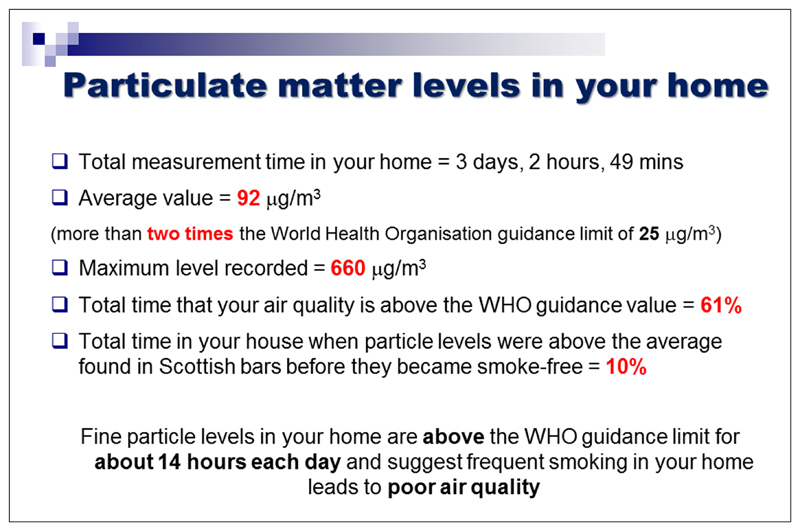
Examples of current feedback package at the time of the needs analysis.

We did not aim to recruit a representative population sample for this phase of the study. Instead, we recruited participants from groups that had no prior experience of having their air quality measured. Some had established smoking restrictions in their own homes, but others had experienced barriers in doing so, relating to sole-parenting, lack of outdoor home space, or lack of motivation. The focus groups included a brief introduction to air quality monitoring as a means of reducing second-hand smoke exposure in the home. Parents were asked for their views on current air quality feedback packages used with a view to improving the current feedback package outlined in Figures 4–6. The discussion elicited parents’ views on ease of understanding the graphs, their views on presenting personalised air quality feedback as a motivational tool for health behaviour change, and their preferences for receiving personalised feedback across a 24 hour period, a one week period, or both. The focus group discussions were digitally audio-recorded and transcribed. Two members of the research team identified key themes independently, which were then discussed with agreement reached on findings and their implications for programme development. Ethical approval was obtained from the University of Aberdeen College Ethics Review Board.

#### Results

##### i Format of delivery

Professionals agreed that providing parents with personalised air quality feedback is credible, and that providing ‘proof’ of indoor second-hand smoke levels has the potential to change smoking behaviour and reduce second-hand smoke levels in the home. This finding was supported by our rapid review of successful interventions using objectively assessed feedback to motivate health behaviour change, which found that presenting personalised feedback to individuals may increase motivation to change their health behaviour by removing the perception of the alternative health risks as hypothetical (Bovet et al. 2002; Van Hoye et al. 2015). Professionals also agreed that feasibility of change is important, and that there are some circumstances where there is ‘*no realistic chance of going outside*.’ Having a close existing relationship with parents was considered key to successful recruitment of parents to air quality feedback studies. Professionals valued the importance of parents being able to see changes in their air quality levels at follow up visits, and spoke of an ‘ideal’ intervention taking between one to five days.

##### ii Air quality feedback

Professionals were asked specifically about ways in which current air quality feedback packages (see Figures 4–6) could be improved.

Professionals valued being able to discuss peaks in second-hand smoke levels with parents, and the day and time axis enables parents to identify particular events or reasons why second-hand smoke levels changed. Explanations of second-hand smoke levels by day, and across the duration of the time for which the monitor was installed was considered useful. Professionals felt that parents focus more on the visuals of the graph than on the accompanying information.

**Figure 7:**
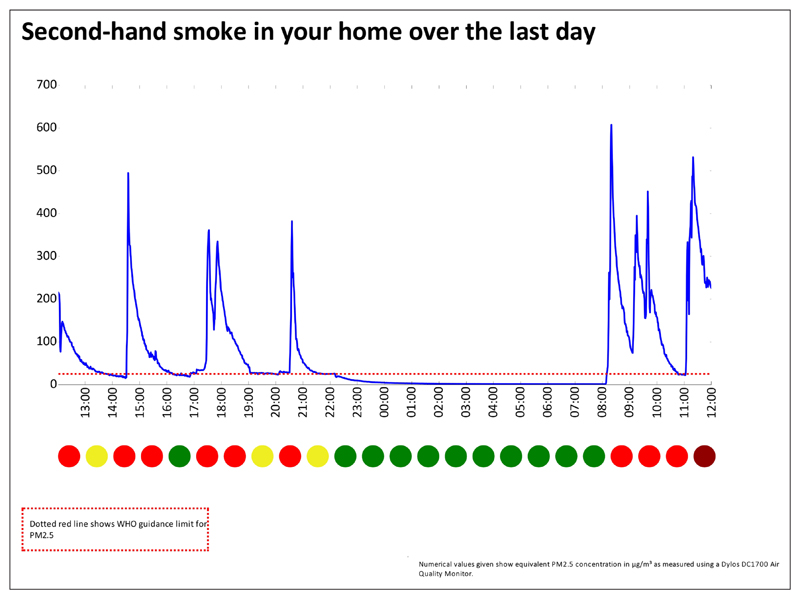
Examples of revised personalised feedback from a home where smoking takes place. Dotted red line shows WHO guidance limit for PM_2.5_. Numerical values given show equivalent PM_2.5_ concentrations in μg/m^3^ as measured using a Dylos DC1700 Air Quality Monitor.

**Figure 8:**
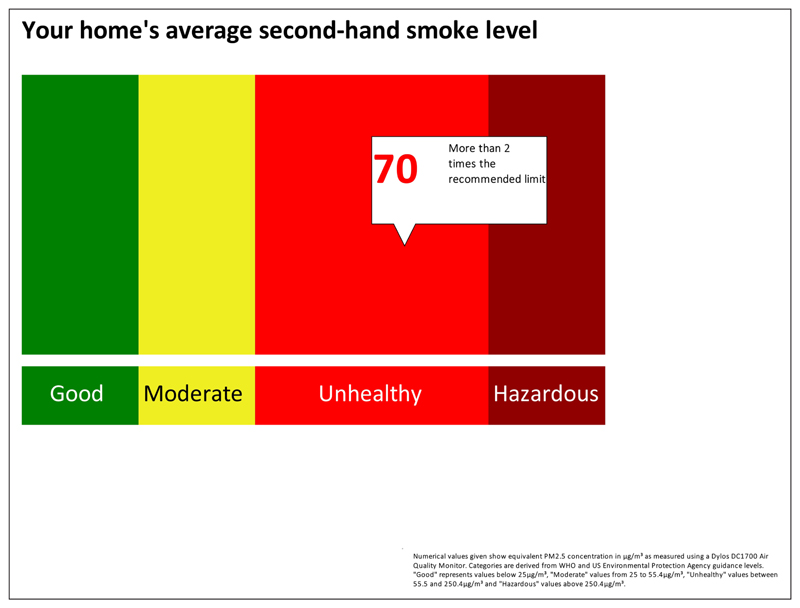
Examples of revised personalised feedback from a home where smoking takes place. Numerical values given show equivalent PM_2.5_ concentrations in μg/m^3^ as measured using a Dylos DC1700 Air Quality Monitor. Categories are derived from WHO and US Environmental Protection Agency guidance levels. ‘Good’ represents values below 25 μg/m^3^, ‘Moderate’ represents values from 25 to 55.4 μg/m^3^, ‘Unhealthy’ represents levels between 55.5–250.4 μg/m^3^ and ‘Hazardous’ represents values above 250.4 μg/m^3^.

Most parents found the feedback graph easy to understand. They reiterated the importance of receiving personalised air quality feedback, stating that non-personalised information might not be a sufficient motivator for actual behaviour change. As one parent noted, *“If you see a graph of someone else’s levels, you could always just say ‘well, mine would be lower than that anyway’.”* Parents suggested using feedback on baseline second-hand smoke levels more explicitly as a motivator for behaviour change and stressed the importance of using non-judgemental language when readings are higher than anticipated. On this basis the air quality feedback package was further developed as per Figures 7–8.

To represent air quality over time in the home, a hybrid visualisation was developed, based on a simplified version of the US Environmental Protection Agency’s Air Quality Index (2014) representing hourly average levels of PM_2.5_, along with a simplified version of Figures 4–6. To allow participants to make a clear connection between recent events in their home and the quality of their air, this visualisation was presented only for the last twenty-four hours of measurement. To emphasise the importance of the average level of PM_2.5_, a colourful visualisation was developed using the VizHealth toolkit (The Regents of the University of Michigan & the Robert Wood Johnson Foundation 2014), comparing the average PM_2.5_ level to the same air quality index used to display hourly average levels.

AFRESH software (The University of Aberdeen, 2016) was developed in tandem with the development of programme materials to enable health/care professionals to download and interpret air quality data, and present air quality feedback to parents. We also developed the AFRESH programme manual (2016) (see Supplementary file 7 – programme manual and materials) providing practical information on how to work with parents through the programme, and how to use the materials as part of the intervention.

##### iii The AFRESH programme

**Figure 9:**
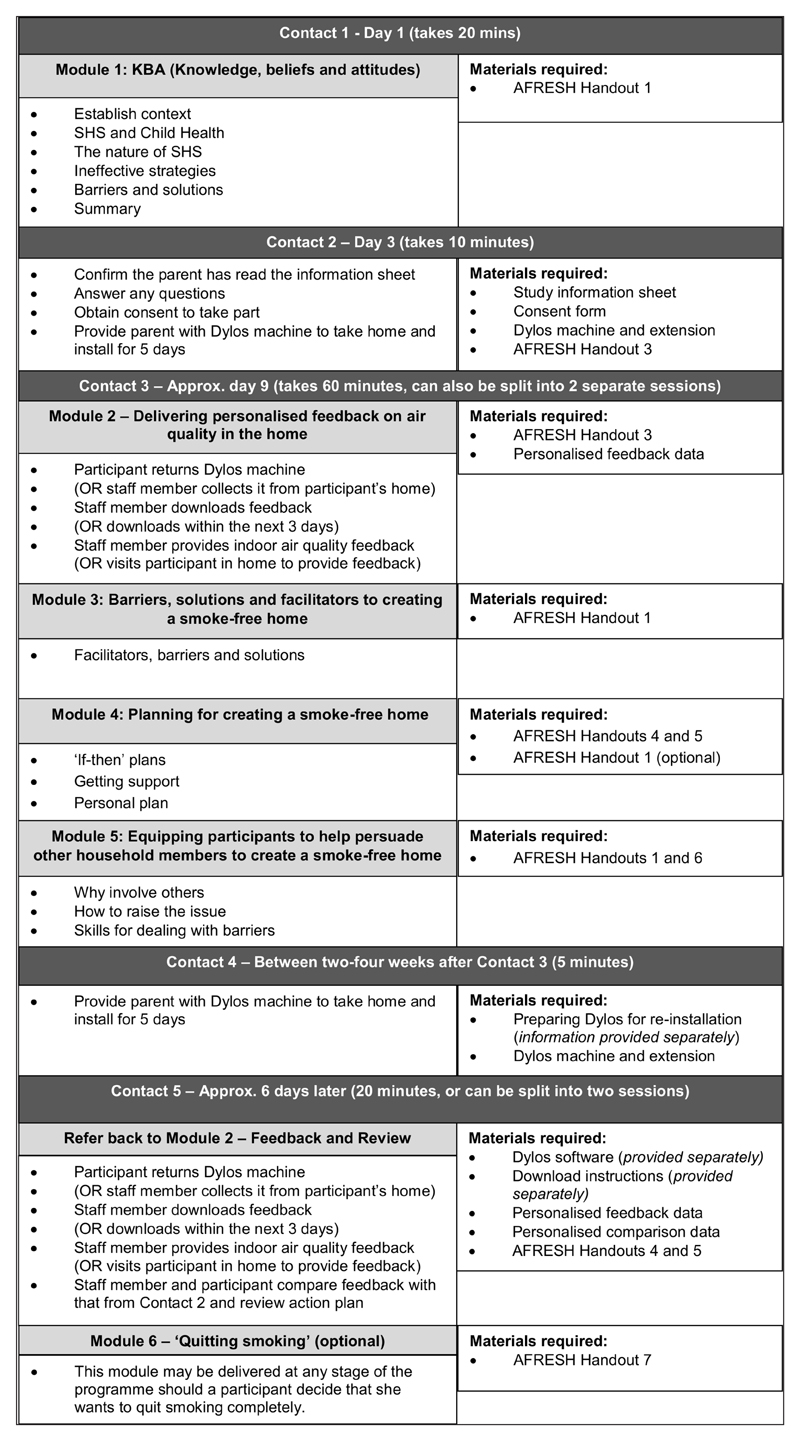
The AFRESH programme flowchart.

The AFRESH programme consists of 6 modules designed to be delivered using face to face discussion techniques, alongside baseline and follow up measures of household second-hand smoke levels. Parents can self-install air quality monitors (Dylos DC1700) and return them to their health/care contact, eliminating the need for home visits. Figure 9 outlines the AFRESH intervention and accompanying materials. Table 4 shows how the AFRESH intervention and materials related to the performance and change objectives/methods identified during the intervention mapping process.

The AFRESH programme consists of five contact points over the course of approximately 1 month, although the programme could run over a longer time span if required. Each point of contact ranges from between five and sixty minutes in duration, although the sixty-minute session may be broken down into shorter sessions. Working through four core modules, health/care professionals establish parents’ current smoking practices, level of knowledge, and their beliefs and attitudes related to second-hand smoke exposure in the home (Module 1). Parents are then invited to take part in the AFRESH intervention, and they are given an air quality monitor to install in their home for a minimum of 5 days, together with written information on the AFRESH intervention.

The air quality monitor is returned to the health/care professional at the start of Module 2, so that air quality data can be downloaded using the AFRESH software. The health/care professional then arranges a suitable time to provide the parent with personalised air quality feedback. Following discussion of air quality levels in the home, the professional discusses parent facilitators and barriers to creating a smoke-free home. They then work through Module 4 with the parent, explaining that behaviour change is helped by a detailed plan of how parents are going to change their behaviour. The parent then develops a personal smoke-free home action plan, and the care professional encourages them to make a list of ‘if-then’ plans (i.e. ‘*If it is a weekday morning, then I will go outside and have a cigarette before my partner goes to work.*’). During this module, the parent is encouraged to identify sources of support who will encourage them in their behaviour change attempt.

Between two and four weeks later, parents install the air quality monitor again to record second-hand smoke levels in their home for another minimum of five days. Health/care professionals download the air quality data as before and prepare personalised air quality feedback. When parents receive their feedback this time, they compare it with baseline levels, and review the personal smoke-free homes action plan accordingly. The health/care professional praises any success, discusses any benefits and setbacks, and reviews the goal as appropriate. Where a smoke-free home has been obtained, techniques are discussed for maintaining this in the long term.

The AFRESH programme also contains two optional modules. Module 5 contains guidance on creating a smoke-free home with the help of visitors/other household members. It offers advice on effectively raising the issue with others, on skills for dealing with family barriers, and on planning for a smoke-free home together. Module 6 can be used at any time in the programme, should a parent decide that they want to quit smoking. It includes materials based on current cessation guidelines in Scotland (NHS Health Scotland 2017).

One of the outcomes of the semi-structured interviews with professionals conducted during Step 4 was that they needed to be better trained in the skills required to deliver elements of the intervention including dealing with situations where parents are unable to realistically take their smoking outside. On this basis, we developed AFRESH training (available from the authors on request) to accompany the AFRESH programme manual, based on existing materials already utilised by the research team (ASH Scotland, the University of Aberdeen & the University of Edinburgh 2012) and the British Psychological Society/Department of Health’s ‘Improving Health: Changing Behaviour’ NHS Health Trainer Handbook (Michie et al. 2008). The training included an overview of the theoretical underpinnings for the intervention and a general introduction to the topic area of second-hand smoke exposure in the home.

## Discussion

This article describes the systematic use of the IM protocol (Bartholomew et al. 2016) to develop a theory- and evidence-based smoke-free homes intervention. The AFRESH programme was specifically designed and developed to address the lack of feasible and effective smoke-free homes interventions available for use with disadvantaged households. Based on our rapid review findings, the intervention was developed to be delivered by health/care professionals, involving face to face communications with parents. It included MI techniques and a focus on providing parents with personalised air quality feedback. We built on previous studies to develop an arm of the intervention that could be used by parents to support their partners/other household members in assisting with the creation and maintenance of a smoke-free home.

We found that discussion, elaboration, individualisation, goal setting, planning coping responses and building skills for resistance were methods well suited to encouraging smoking parents to create a smoke-free home. We utilised feedback from parents and health/care professionals on the design and execution of air quality monitoring studies, and involved potential AFRESH programme centres from the outset of this work to assist with securing implementation.

In order to take account of identified implementation barriers, we developed a tailored AFRESH training programme, a step-by step guide to delivering the intervention, and a range of tailored support materials to use with parents, depending on their individual needs. We planned an intervention with feedback of personalised air quality levels in the home at baseline, and a short-term follow up measure between two and four weeks later.

During the course of developing the AFRESH programme, the use of IM served as a useful protocol to ensure the integration of theory, evidence and existing practice in this field. It is a thorough process which requires a systematic approach, but when followed step-by step it serves to ensure that all intervention objectives are sufficiently addressed through design and development phases.

## Conclusion

This paper demonstrates that it is feasible to adopt an intervention mapping approach to develop a rigorous, theory- and evidence-based smoke-free homes intervention for use with disadvantaged households.

## Supplementary Material

Supplementary file 1

Supplementary file 2

Supplementary file 3

Supplementary file 4

Supplementary file 5

Supplementary file 6

Supplementary file 7

Supplementary file 8

## Figures and Tables

**Table 1:** Matrix 1 – Performance objectives, determinants and change objectives related to creating a smoke-free home.

Determinants	Knowledge	Perceived severity	Perceived susceptibility	Beliefs/Attitudes	Social norms and support	Self-efficacy/Skills
Behaviours						
– Decide to make home smoke-free	– Explain the health risks associated with childhood second-hand smoke exposure in the home – Explain that there is no safe level of exposure to second-hand smoke – Recall the strategies that are ineffective in removing risk completely	– Recognise that exposure to second-hand smoke in childhood is a serious health threat – Recognise that even the occasional relapse has an impact on child health	– Recognise that their child/children are susceptible to the health risks associated with second-hand smoke exposure in the home – Recognise that their own smoking behaviour is creating high levels of second-hand smoke in their home	– State that having a smoke-free home will make the house look/smell nicer – Explain that having a smoke-free home will better protect child/children from health risks – Explain that having a smoke-free home will reduce the likelihood of their children becoming smokers themselves – Identify that creating a smoke-free home can be a useful stepping stone to quitting		– Plan to make the home smoke-free either in one go, or through making incremental changes
– Explore and identify barriers that are most likely to impact on their own ability to create a smoke-free home	– Define the general barriers to creating a smoke-free home – Describe how smoking ‘norms’ within the family home may act as a barrier to behaviour change					– Identify the domestic circumstances that need to change for a smoke-free home to be achievable
– Identify and implement solutions that are most likely to assist them in creating a smoke-free home	– Describe effective strategies for exploring solutions					– Conclude that identified barriers can be overcome with the solutions discussed and provided
– Self-monitor goal progress and respond by resolving problems if discrepancies occur between the goals set and actual behaviour	– Recognise that people are more successful if they see ‘failure’ as a learning opportunity			– Express that the benefits of a smoke-free home to outweigh the costs – Recognise that good planning of a smoke-free home is important to the success of the attempt	– Recognise that most parents in the same situation are struggling on occasions	– Value the personalised action plan for making the home smoke-free

**Table 2:** Matrix 2 – Performance objectives, determinants and change objectives related to engaging other household members in creating a smoke-free home.

Determinants	Knowledge	Beliefs/Attitudes	Self efficacy/Skills
Behaviours			
Convince others to make the home smoke-free	– Recognise that the child will only fully benefit if others also smoke outdoors – Identify that they are not asking others to quit smoking altogether, but to smoke outside. – List effective ways to open up a discussion with family and friends about creating a smoke-free home – Demonstrate effective responses to others’ questions about creating a smoke-free home.	– Recognise that the advantages (health, smell, etc) of trying to have household members also smoke outside outweigh the disadvantages (effort, conflict) – Express that creating a smoke-free home together is achievable. – Recognise that it is their responsibility to protect the child against second-hand smoke from others	– Identify that the personalised feedback is an efficient way for opening up the discussion about others’ roles in creating second-hand smoke in the home. – Express confidence in ability to share PM2.5 feedback with others – Express confidence in their ability to ask others in the home/visitors for their support in making the home smoke-free. – Discuss effectively with others the reasons why some strategies are ineffective for creating a smoke-free home.
Explore/identify together barriers that are most likely to impact on others’ abilities to create a smoke-free home.	– Demonstrate how to use the intervention materials to help others identify barriers to making the home smoke-free.	– Recognise it is important to identify concrete problems and solutions together, for this to work	– Express confidence in their ability to support others to overcome barriers
Identify and implement solutions that are most likely to assist others in creating the smoke-free home.	– Demonstrate how to use intervention materials to help others explore solutions to making the home smoke-free.	– Conclude that family/friends will be able to implement the effective solutions consistently	– Express confidence in ability to support/continue to motivate others to implement solutions
Monitor household-goal progress and respond by resolving problems if discrepancies occur between the goals set and actual behaviour		– Express motivation to keep the home smoke-free despite possible relapses or ongoing struggles with household members	– Express confidence in ability to raise problems with family/friends if discrepancies occur

**Table 3:** Examples of methods and applications for change objectives related to behavioural determinants.

Determinant	Change objectives	Method	Definition	Parameters for use	Practical application

Knowledge	Explain the health risks associated with childhood second-hand smoke exposure in the home Explain that there is no safe level of exposure to second-hand smoke Define the general barriers to creating a smoke-free home Recognise that the child will only fully benefit if others also smoke outdoors List effective ways to open up a discussion with family and friends about creating a smoke-free home.	Discussion	Encouraging consideration of a topic in open informal debate.	Listening to the learner to ensure that the correct schemas are activated.	Informal discussion of the health risks, barriers and solutions for creating a smoke-free home.
		Elaboration	Stimulating the learner to add meaning to the information that is processed.	Motivated and cognitively able individuals. Messages must be relevant and easy to understand	Encouraging the parent to apply knowledge to their own situation, with personalised air quality feedback.
Perceived severity	Recognise that exposure to second-hand smoke in childhood is a serious health threat Recognise that even the occasional relapse has an impact on child health Recognise that their child/children are susceptible to the health risks associated with second-hand smoke exposure in the home Recognise that their own smoking behaviour is creating high levels of second-hand smoke in their home.	Individualisation	Providing opportunities for learners to have questions answered.	Personal communication that responds to a learner’s needs.	Individual air quality feedback sessions with opportunities for parents to have questions answered, and discussion regarding solutions to actual and perceived barriers.
Perceived Susceptibility	
Beliefs and Attitudes	Explain the benefits of a smoke-free home Value their attempts to create a smoke-free home Recognise that good planning of a smoke-free home is key to success Conclude that identified barriers can be overcome with the solutions discussed/provided Express that creating a smoke-free home together with other family members is achievable	Elaboration	As above	As above	General discussion on creating a smoke-free home whereby the parent is encouraged to add meaning to messages i,e ‘I would value having a smoke-free home because…’
Social norms and support	Recognise that smoking doesn’t occur in most homes Arrange practical support from family and friends to create a smoke-free home for child Organise support from others in the household to implement the solutions	Resistance to social pressure	Stimulating building skills for resistance to social pressure.	Commitment to earlier intention	Rehearsing situations where social pressure may occur and developing effective solutions for dealing with it.
Self-efficacy	Value the personalised action plan for creating the smoke-free home Identify the domestic circumstances that need to change in order for a smoke-free home to be achievable Conclude that identified barriers can be overcome with the solutions discussed/provided Express confidence in their ability to ask others in the home/visitors for their support in making the home smokefree. Express confidence in their ability to share personalised feedback/second-hand smoke information with others	Planning coping responses	Prompting participants to list potential barriers and ways to overcome these.	Identification of high-risk situations and practice of coping response.	The parent could engage in a motivational type interview with family members to identify and overcome barriers. Rehearsal strategies could also be utilised to practice coping responses, i.e. ‘if X [high risk situation] occurs, then I will do Y to overcome it.’

**Table 4:** The AFRESH programme: Outline of content in relation to selected behaviour change methods and targeted determinants.

	Outline of content	Behaviour change methods selected	Determinants targeted	Accompanying Fact Sheet
**Module 1: Parent knowledge, beliefs and attitudes regarding second-hand smoke in the home**	Establish context Second-hand smoke and child health – The effects of second-hand smoke and how they apply to your own child/children Ineffective strategies for eliminating second-hand smoke in the home Barriers and solutions to creating and maintaining a smoke-free home Summary	Tailoring Discussion Elaboration	Knowledge Self-efficacy Perceived susceptibility	Fact Sheet 1: What you need to know about second-hand smoke Fact Sheet 2: second-hand smoke and effects on children’s health Handout 1: Parent barriers, facilitators and solutions for creating a smoke-free home Handout 2: Colour print out of example feedback graph from a home where smoking takes place
		Discussion	Knowledge	
		Discussion	Knowledge	
**Module 2: Delivering personalised feedback on air quality in the home**	Deciding whether to have air quality measured in the home Reinforcing positive steps already taken Install the air quality monitor Providing feedback on personalised air quality levels	Self-affirmation	Self-efficacy	Handout 3: Your guide to the air quality intervention
		Individualisation Personalised risk Goal setting	Knowledge, Self efficacy	
**Module 3: Barriers, solutions and facilitators to creating a smoke-free home**	Facilitators to creating a smoke-free home Barriers to creating a smoke-free home	Elaboration Planning coping responses	Beliefs Self-efficacy, Knowledge, beliefs	Handout 1: Parent barriers, facilitators and solutions for creating a smoke-free home
**Module 4: Planning for creating a smoke-free home**	Developing a personal smoke-free home action plan Difficult situations and ‘if-then’ plans Getting support Personal action plan Mini-goals Reviewing the action plan Praising any success Building habits	Discussion Elaboration	Knowledge, beliefs, self-efficacy	Handout 4: Personal smoke free home action plan
		Discussion	Knowledge, self-efficacy	Handout 5: Difficult situations and ‘if-then’ plan Handout 1: Parent barriers, facilitators and solutions for creating a smoke-free home (optional)
**Module 5: Delivering the intervention to others in the household (optional)**	Why involve other family members in creating a smoke-free home? How to raise the issue of creating a smoke-free home with other household members Skills for dealing with family barriers to creating a smoke-free home	Discussion	Knowledge	Handout 1: Parent barriers, facilitators and solutions for creating a smoke-free home
		Discussion	Knowledge, self-efficacy	
		Planning coping responses Resistance to social pressure		
**Module 6: Quitting smoking (optional)**	Why quit? What tools can help with quitting? What else can help?			
