# Supplementary material for: Development of a Smoke-Free Homes Intervention for Parents: An Intervention Mapping Approach
Source: Health Psychol Bull. Author manuscript; Available in PMC 2020 Apr 24. (PMC7182446; doi:10.5334/hpb.20)
Supplement: Supplementary file 3 [file EMS86099-supplement-Supplementary_file_3.docx]

**Supplementary file 3**

**Review on Behavioural Interventions to Reduce Indoor Smoking by Parents**

**Objective:** Our initial intention was to conduct a review of published papers to investigate the effectiveness of behavioural interventions which promote second-hand smoke reduction (SHSr) by parents. However, a recent systematic review by Brown and colleagues (2015) has been published which included a similar objective. Therefore, the current rapid review will synthesise findings from the identified papers in the Brown et al. (2015) review, with a specific focus on identifying the important behavioural and psychological components involved in SHSr by parents.

**Eligibility:**

As papers will be taken from the systematic review performed by Brown et al. (2015), the eligibility criteria will initially match the criteria stated in their review, including: 1/ empirical study reports of interventions aimed at promoting a smoke free home environment; 2/ primary carers (parents, guardians, foster carers or grandparents) involved in the parenting of infants and young children and/or young children. Where child age range exceeded 0–5 years, a mean age within the 0–5 year range was used as a criterion. Included papers were published between 2000 and 2014 in peer reviewed journals to ensure a focus on the most recent research in the topic. Papers were excluded if they were not written in English.

The same study numbers will be used as Brown et al. (2015) to allow for easier cross referencing.

Additionally, the following eligibility (Table 1) must also be met for the requirements of the current rapid review.

**Table 1: Eligibility Criteria**

| **Population/problem** | **Intervention** | **Outcome** | **Study design** |
| --- | --- | --- | --- |
| Parents who smoke/SHS | Any intervention that has SHS reduction as a primary or secondary outcome. | Changes in indoor smoking behaviour by parents. Should have a subjective and/or objective measure of SHS. | Randomized Controlled Trials (RCT’s) |

**Information Sources:** Electronic databases searched by Brown et al. (2015) included; MEDLINE, Cochrane Database of Systematic Reviews, PubMed, and CINAHL. Search terms included cigarettes, smoking, tobacco, parent, and family, as well as terms aimed at identifying intervention studies. The reference lists of included studies were searched manually by Brown et al. (2015).

**Inclusion/Exclusion:** Twelve papers were reviewed for SHSr by Brown et al. (2015). Five of these papers were excluded from the current rapid review (please see reasons for exclusion in Table 2). The current review has included an additional paper (study 58) which Brown et al. (2015) identified as a smoking cessation paper, however on closer examination this paper also focussed on SHSr. Therefore, eight papers in total will be included in the current rapid review.

**Table 2: Exclusions**

| **Reference** | **Reason for exclusion** |
| --- | --- |
| Fossum et al.  2004. Sweden [58] | SHSr or indoor smoking was not measured therefore outcome eligibility not met. |
| Chan-Yeung et al. 2000; Becker et al. 2004, Chan-Yeung et al. 2005. Canada [55–57] | [55] Does not state how data was measured therefore outcome eligibility criteria not met.  [56] No specific results or measurements given for SHSr therefore outcome eligibility criteria not met.  [57] No results given for SHSr measurements therefore outcome eligibility criteria not met. |
| Huang *et al.* 2013. Taiwan [60] | Focus was on intention to reduce SHS rather than actual behaviour change and no measurement of actual/perceived SHS change in the home was recorded, therefore intervention and outcome eligibility criteria not met. |

**Summaries of Included Papers**

Brief summaries of the included papers has been provided. For more detail regarding design and outcomes, please see Table 4, Appendix A.

**Hovell et al. (2009). USA [34]:**

The intervention was a counselling intervention based on the Learning Theory which included: behavioural contracting, self-monitoring and problem solving. Nicotine Replacement Therapy (NRT) was offered to the intervention group (IG) throughout the trial but only to the control group (CG) after the final study measure was taken, which may have introduced bias to the results of the study. Participant objectives which were achieved, resulted in positive feedback and prompting to do more.

Hovell and colleagues reported that SHS was reduced, which was measured by self-reports and confirmed through cotinine and nicotine assays (although not to a significant level).

The author’s also state that the study shows that about two thirds of the experimental effect may be accounted for by measurement reactivity. This highlights that the presence of an objective measurement may have an effect per se, even before providing feedback, which should be taken into consideration when designing a controlled trial.

This study offered fourteen counselling sessions but the authors highlight that high risk families may find it difficult to attend all of these due to other difficult life events including: unemployment, domestic abuse, and alcohol or other drug abuse. Thus, when designing interventions of this nature, it may be beneficial to keep visits/sessions to a minimum.

**Yücel et al. (2014). Turkey [49]:**

Five behavioural change techniques were used during the intervention: (i) providing information, (ii) goal setting behaviour (not smoking in the home) and outcome (to reduce children’s SHS exposure), (iii) use of follow-up prompts, (iv) educating to use prompts (i.e. hanging ‘no smoking’ warning signs in the home) and (v) environmental restructuring (i.e. removing ashtrays in the house). Language that appeals to fathers was used in the information pamphlets due to the impracticality of face-to-face interviews with the father’s and the high prevalence of fathers’ smoking.

The intervention was successful for reducing SHS in the home which was measured through self-reports and confirmed through urinary cotinine measurements. Again, this study provides support that minimal contact with smokers can be as effective as numerous sessions as there was no significant difference in changes between the intense intervention and the minimal intervention in regards to SHSr. However, the intense intervention did appear to have a positive effect on smoking bans compared to the minimal intervention due to the intensive intervention strategy that kept the issue on the agenda by repeating the education.

The change in both intervention groups in this study suggests that informing parents about children’s SHS exposure (education provision during the home visit) and confirming it using laboratory tests (feedback of cotinine levels) was an efficient strategy.

**Harutyunyan et al. 2013. Armenia [50]:**

The intense intervention which included an MI based counselling session, the provision of personalised air quality feedback, a tailored educational brochure and two follow-up counselling telephone calls, had a significant effect on SHSr. However, there were no significant differences between the intense intervention group and the brief intervention group whereby the brief intervention group received only a brief educational leaflet regarding SHS hazards. Therefore this study highlights a need for a cost-effective evaluation to justify implementation of the intensive intervention. SHSr was measured through children’s hair nicotine concentrations and self-reports.

The personalised air quality feedback provided was measured using an “Aerosol Sidepak” machine. In addition, outdoor air quality feedback was also provided for comparison with the indoor air quality.

The author’s report that the intense intervention had a more positive effect on educating non-smoking mothers regarding SHS hazards and also the promotion of smoking restrictions at home, compared with the brief intervention.

**Zakarian et al. 2004. USA [51]:**

The study found that behavioural counselling with parents for reducing children’s SHS exposure was not effective when added to well-child health care services at community clinics. However, the intervention was not delivered as planned due to high staff turnover, training issues and funding. Self-reported SHS reduced but urinary cotinine concentrations did not. Self-reported mother’s indoor smoking and mother’s overall smoking significantly decreased.

Study 51 was conducted by many of the same authors as study 34. The intervention used in study 34 appears to be a very similar intervention as study 51 but used at a later date in a different setting. The main difference between both these interventions is that study 51 did not combine SHSr with smoking cessation counselling.

**Emmons et al. 2001 [52]:**

The MI counselling intervention was successful as it significantly reduced SHS levels in the home, whereas the Self-Help (SH) group’s level of SHS increased, although not to a significant level. The differences of effects between each of these groups reveal that educational materials alone, which were provided to the SH group, are not sufficient to reduce children’s SHS exposure in smoking households.

The study demonstrated that the provision of objectively assessed feedback of SHS levels and MI counselling strategies including adopting a supportive problem-solving approach to addressing the parents’ barriers to SHSr, are effective components for helping parents to reduce SHS in their homes.

The author’s also report that the interventionists received extensive training and on-going supervision which may have had a positive impact on the effectiveness of the intervention.

**Baheiraei et al. 2011. Iran [53]:**

The intervention (MI counselling, phone counselling and education provision) was successful as it significantly reduced: infant urinary cotinine levels, the amount of cigarettes consumed in the presence of the child (self-reported), and increased home-smoking bans, compared to the CG (usual care).

The study demonstrates that previously reported successful MI counselling interventions for SHSr in the family home can be generalized to Iran and also that it is beneficial to target both the mothers and the fathers to take action to protect their children from SHS exposure.

**Wilson et al. 2013. Scotland [54]:**

The feasibility study demonstrated that using objectively assessed air quality feedback to mothers, in addition to MI sessions can have a positive effect on SHS reduction in the home (measured by PM2.5) as SHS exposure was significantly reduced from receiving the feedback to the follow-up session (approx. four weeks later).

Decreases were found in salivary cotinine concentrations although not to a significant level, which suggests that the PM2.5 is a more sensitive measurement of SHS exposure than cotinine concentrations.

**Conway et al. 2004. USA [59]:**

This behavioural problem solving intervention, which was delivered by lay women, was not effective in significantly reducing SHS exposure in the home as no differences between groups for self-reported SHS exposure and hair cotinine levels were found.

The author’s suggest that any changes between baseline and follow-up may be have been prompted simply by participating in the measurement portion of the study. Overall, this was a costly intervention with little effects.

A possible reason for the intervention ineffectiveness could be related to the complex intervention being delivered by lay people untrained in behaviour change theory and methods, which again highlights the importance of adequate training of the interventionists.

**Table 3. Characteristics of intervention:**

| Author | Content | Delivery Personnel | Method of communication | Intensity/  Complexity | Enviro-nment | Conceptual Framework |
| --- | --- | --- | --- | --- | --- | --- |
| Hovell et al. 2009  [34] | 10 in-person at home and 4 telephone counselling sessions over 6 months and additional pre- and post-quit telephone sessions. Counselling procedures included behavioural contracting, self-monitoring, and problem solving. All smokers in the counselling group families were offered free nicotine patches and/or gum to assist with quit attempts. | Study counsellor (research assistants). Received 30 hrs of training. | Face to face + phone. | 14 x biweekly sessions over 6 months (10 x face to face + 4 x phone). Mean Time for each session: 23 minutes | Home | Learning theory (including behavioural contracting, self-monitoring and problem solving). |
| Yucel et al. 2014  [49] | On the initial home visit, mothers were interviewed in order to complete the initial form and were trained using brochures. An intermediate follow-up survey form was administered to the mothers during the second home visit, which occurred within 1 month after the initial visit. The parents were also informed about the urinary cotinine levels of the samples taken from the children in the course of the initial home visit. The mothers were telephoned twice within an interval of 2 weeks to learn the smoking status at home. One month after the second telephone interview, the final home visit was carried out, the final follow-up survey form was administered to the mothers, and a second urine sample was obtained from the children. | Researcher. Training not stated. | Face to face + phone + written materials. | Intensive group: 3 x home visits (at baseline, 1 & 3 months). 2 x phone calls at 6 & 8 weeks. Minimal intensity group: home visit at baseline and 3 months. Mail out urine cotinine result. | Home | Not stated but Five behavioural change techniques were used during the intervention: (i) providing information, (ii) goal setting behaviour (not smoking in the home) and outcome (to reduce children’s ETS exposure), (iii) use of follow-up prompts, (iv) educating to use prompts (i.e. hanging ‘no smoking’ warning signs in the home) and (v) environmental restructuring (i.e. removing ashtrays in the house). |
| Haruty-unyan  et al [50]. | Counselling sessions, distribution of tailored educational brochures, demonstration of home air pollution, and 2 follow-up counselling telephone calls. Intervention methods included: Importance of healthy environment, dangers of smoking and SHSe, smoking cessation, smoke-free home, PM2.5 feedback and written materials. CG: written materials only. | Research staff. Training intensity not stated. | Face to face + written material + phone. | 40 minute MI + 2 follow  up phone calls  (timeframe not specified) | Home | MI. |
| Zakarian et al.  2004 [51] | Behavioural counselling programme. Intervention consisting of 7 behavioural counselling sessions over 6 months. Counselling included behavioural contracting to reduce SHSe, self-monitoring, problem solving, goal setting and positive reinforcement. | Health educators (trained in MI by an experienced MI trainer). Nurses, Medical assistants.  Very intense comprehensive training provided. Received 6 hrs of initial training and then ongoing training and supervision. | Face to face. | 7 counselling sessions over 6 months. | Clinic (× 3) + home via phone (× 4). | SLT (Bandura 1977)  and behavioural  ecological model  (Hovell, Wahlgreen &  Gehrman, 2002 [ref]) |
| Emmons et al.  2001 [52] | Motivational intervention. The MI condition consisted of a 30- to 45-minute motivational interviewing session at the participant’s home with a trained health educator and 4 follow-up telephone counselling calls. Feedback from baseline household air nicotine assessments and assessment of the participant’s carbon monoxide level was provided as part of the intervention. Intervention methods included: Choice, personal responsibility for change, self-efficacy, feedback on CO level. Tailored to interest in quitting smoking or reducing SHSe. Goal setting. | Health educator. MI training provided and ongoing training and supervision. | Face to face + phone. | 1 x 30–45 MI + 4 follow  up phone calls | Home | MI |
| Baheira-ei et al.  2011 [53] | Motivational intervention.  Mothers of the intervention group were provided 3 counselling sessions, one of which was face to face and 2 of which were by telephone. Fathers were provided 3 counselling sessions by telephone. Parents were also given an educational pamphlet and a sticker depicting a smoke-free home. MI used and counselling sessions based on “Smoke-free children” method (Arborelius & Bremberg, 2001; Fossum et al., 2004; Greenberg et al., 1994). | Research student. Training intensity not stated. | Face to face, phone + written  Materials. | 1 x face to face interview  + 2 x phone interviews with mothers or 3 x phone interviews with fathers (max: 20 min each). Parental questionnaire | Home | MI |
| Wilson et al. 2013  [54] | Four home visits over a 1-month period, which involved two 24-h measurements of home air quality (PM2.5) and a motivational interview to encourage changes to smoking behaviour within the home in order to reduce child SHS exposure. The enhanced group received their air quality data as part of their motivational interview at visit 2; the control group received that information at visit 4. | Research staff (trained in MI techniques). Intensity of training not stated. | Face to face. | 4 x visits over a 1 x month period. | Home | MI |
| Conway et al.  2004 [59] | A tailored behavioural problem solving intervention. IG guided by lay community health advisors to do problem solving aimed at lowering the target child’s exposure to SHS in the household. Intervention methods included contracting, shaping, positive reinforcement, problem solving, social support and identifying barriers, to assist families achieve their SHS reduction goals. | Lay bicultural and bilingual Latina community health advisors. All received 20 hrs of training over 4 weeks. | Face to face + phone. | 6 x sessions over 4 months. | Home | Operant and social learning theory. |

The intervention characteristics for each included study have been highlighted in Table 3 and will be discussed in the following synthesis.

**Synthesis:**

A narrative synthesis has been conducted by extracting relevant information from the brief summaries, intervention characteristics (Table 3) and the overall review table (Table 4; Appendix A).

**Objectively Assessed Feedback Data**

Half of the reviewed studies (49, 50, 52, 54) used objectively assessed feedback as part of the intervention including; urinary cotinine feedback (49) and air quality feedback (50, 52, 54). All of these four interventions were successful in significantly reducing SHS in the home when objectively measured. Of the four studies that did not use objectively assessed feedback as part of the intervention; study 34 found that child urine cotinine decreased quicker in the intervention group than the control group but the final differences were not significant; study 51 results show that children’s reported SHS exposure declined for both groups but were not significantly different from each other. Study 51 also found that urinary cotinine concentrations did not significantly change over time for either the intervention group or the control group. Study 53 reported that urine cotinine levels decreased for both conditions, but only to a significant level for the intervention group. No significant differences were found for any of the measured variables in study 59, including confirmed SHS reduction. Thus, 100% of the interventions which included objectively assessed data as a specific part of the methods used could be deemed as successful interventions for reducing SHS compared to 25% of the interventions which did not include objectively assessed data as part of the intervention, implying that implementing objectively assessed data as part of a SHSr intervention is an important component for this type of behaviour change.

**Measurement Methods of SHSr**

Five out of the eight studies adopted subjective measurements of SHSr and all of the studies included at least one objective measurement of SHSr. Child Urinary Cotinine appears to be the most common method of objectively assessed data with half of the studies using this method (34, 49, 51, 53). Child salivary cotinine was used in only one of the studies with no significant changes found, implying that this may be the least detectable/useful method of SHSr. Self-reported SHSe was measured in 3 of the 8 studies (34, 53, 59) whereby the results of these self-reports were confirmed by an objective measurement. For example, study 53 found that there were significant changes for self-reported SHSe both within the IG and between the IG and CG which was confirmed by measuring child urinary cotinine which found the same significant changes (within the IG and between groups). Therefore, self-reported SHSe emerges as a reliable method of SHSr measurement.

**Conceptual Frameworks**

Four (50, 52, 53, 54) out of eight studies used MI techniques which all reported significant positive outcomes for SHSr. Three interventions (34, 51, 59) included components taken from the social learning theory comprising of; goal setting and/or behavioural contracting (34, 51, 59); self-monitoring (34, 51); problem solving (34, 51, 59). One intervention (49) did not state a conceptual framework, but did state five behavioural change techniques which were implemented which included goal setting.

**Training Intensity**

The intensity of the training provided to interventionists varied between some studies. Half of the studies did not state the training intensity (49, 50, 53, 54) and of the remaining four studies, two studies provided MI training and also ongoing training and supervision (51, 52), one study provided 20 hours of training (59) and one study provided 30 hours of training (34).

**What have we learned?**

- On average, the majority of studies had at least some effect on SHS reduction in the home.
- Incorporating objectively assessed data and MI appear to be the most popular adopted intervention methods and the most effective for SHS reduction with parents and caregivers of young children.
- Alternatively, sole provision of information is not an effective strategy for this specific behaviour change type.
- Better control measures have to be taken in RCT’s when testing the effectiveness of providing objective feedback, by eliminating the risk of measurement reactivity.
- Extensive training and ongoing supervision provision may play a vital role in the success of SHSr interventions.
- A balance has to be sought to make an intervention intensive enough to be effective but also ensuring too many sessions are not required, as the target population (often socioeconomically disadvantaged people) may find multiple session attendance problematic.
- It is beneficial to include both mothers and fathers in SHSr interventions in the home.

These learning points should be taken into consideration when planning/developing interventions to reduce SHS exposure in the home by parents.

**References**

34. Hovell, M. F., Zakarian, J. M., Matt, G. E., Liles, S., Jones, J. A., Hofstetter, C. R., … Benowitz. N. L. (2009). Counseling to reduce children’s secondhand smoke exposure and help parents quit smoking: A controlled trial. *Nicotine Tob. Res. 11*(12), 1383-1394. doi:[10.1093/ntr/ntp148](http://dx.doi.org/10.1093%2Fntr%2Fntp148)

49. Yucel, U.,Ocek, K. A., & Ciceklioglu, M. (2014). Evaluation of an intensive intervention programme to protect children aged 1-5 years form environmental tobacco smoke exposure at home in Turkey. *Health Educ. Res. 29*(3), 442-455. doi:[10.1093/her/cyu005](http://dx.doi.org/10.1093%2Fntr%2Fntp148)

50. Harutyunyan, A., Movsisyan, N., Petrosyan, V., Petrosyan, D., & Stillman, F. (2013). Reducing children’s exposure to secondhand smoke at home: A randomized trial. *Pediatrics, 132*(6), 1071-1080. doi:[10.1542/peds.2012-2351](http://dx.doi.org/10.1093%2Fntr%2Fntp148)

51. Zakarian, J. M., Hovell, M. F., Sandweiss, R. D., Hofstetter, C. R., Matt, G. E., Bernert, J. T., … Hammond, S. K. (2004). Behavioral counselling for reducing children’s ETS exposure: Implementation in community clinics. *Nicotine Tob. Res. 6*(6), 1061-1074. doi:[10.1080/1462220412331324820](http://dx.doi.org/10.1093%2Fntr%2Fntp148)

52. Emmons, K. M.,Hammond, S. K., Fava, J. L., Velicer, W. F., Evans, J. L., & Monroe, A. D. (2001). A randomized trial to reduce passive smoke exposure in low-income households with young children. *Pediatrics, 108*(1), 18-24.

53. Baheiraei, A., Kharaghani, R., Mohsenifar, A., Kazemnejad, A., Alikhana, S., Milani, H. S., … Hovell, M. F. (2011). *Nicotine Tob. Res. 13*(9), 840-847. doi:[10.1093/ntr/ntr085](http://dx.doi.org/10.1093%2Fntr%2Fntp148)

54. Wilson, I., Semple, S., Mills, L. M., Ritchie, D., Shaw, A., O’Donnell, R., … Amos, A. (2012). REFRESH_Reducing families’ exposure to secondhand smoke in the home: A feasibility study. *Tob. Control*, doi:[10.1136/tobaccocontrol-2011-050212](http://dx.doi.org/10.1093%2Fntr%2Fntp148)

59. Conway, T. L., Woodruff, S. I., Edwards, C. C., Hovell, M. F., & Klein, J. (2004). Interventions to reduce environmental tobacco smoke exposure in Latino children: Null effects on hair biomarkers and parent reports. *Tob. Control. 13*, 90-92. doi:[10.1136/tc.2003.004440](http://dx.doi.org/10.1093%2Fntr%2Fntp148)

Table 4. General Components and Results of Studies Which have Investigated SHSr in the Home with Parents of Young Children.

| Reference | Objective | Sample | Design | Intervention Content | Comparator | How FB was embedded | Results/Outcomes |
| --- | --- | --- | --- | --- | --- | --- | --- |
| Hovell et al.  2009. USA [34] | To test the effects of SHS and smoking counselling in high risk families. | 150 of 244 eligible mothers of children aged  less than 4 years exposed to minimum of 3 maternal cigarettes per day. | RCT | SHSe reduction and tailored smoking cessation including option of NRT. | Usual care (No SHS or cessation counselling). When smoking or SHSe was identified, personnel provided a referral to a free Smokers helpline for counselling. | Objective and subjective measures taken but no mention of providing this as feedback information. | **Reported SHS exposure:** Decrease in both IG (80%) & CG (55%) in first 6 months. Group main effect 6–18 months significant for IG (p = 0.011). Group differences for changes n.s.  **Child urine cotinine:** Decreased baseline to 6 months only (25% both gps). Only the group main effect significant for 6–18 months (p = 0.026). Controls higher throughout baseline & follow up. Group differences for changes n.s. |
| Yücel et al. 2014.  Turkey [49] | To evaluate the effectiveness of an intensive intervention *vs.* a minimal intervention to reduce SHSe. | Mothers of 80 out of 182 children aged  1–5 years, who smoked and/or whose spouses smoked. | RCT | SHSe information, goal setting, use of resources, urine cotinine feedback. | Minimal intervention. Comprising 2 home visits and urine cotinine notification compared to 3 home visits, 2 telephone follow-ups and urine cotinine notification. | Intensive intervention: Parents were informed (at second home visit) about the urinary cotinine levels of the samples taken from the children in the course of the initial home visit. Minimal intervention: This group was notified by mail of the urinary cotinine levels of their children’s samples obtained during the initial home visit. | **Urinary cotinine–pre and post intervention:** Urine cotinine decreased across time in both groups (p < .001). Decrease in the level of cotinine in the final follow-up was higher in the intensive intervention group than the minimal intervention group. However, this difference was not statistically significant on the t-test (P =0.831).  Significant decline in number of cigarettes smoked at home in past 7 days (p = .012).  **Complete home smoking bans**: Authors report that 30.6% of Intensive IG households who did not have a ban at baseline, did have a total ban at 3 months (p = 0.001). In the minimal IG, 10.5% more families had ban at 3 months, but n.s (p = 0.125). Sig difference between groups for smoking bans (p < .001). |
| Harutyunyan et  al. 2013.  Armenia [50] | To test an intense intervention to reduce  child SHSe. | 250 households with children aged 2–6 years recruited via paediatrician primary health care clinics. Maternal age M = 30 years (SD 5.2 years). 53% employed, 36% had a university degree. Household smokers predominately fathers (80%).  Child age M = 4 years (SD 1.2 years). Smoking was permitted in all households, some restrictions in approximately half of homes. | RCT | Importance of healthy environment,  dangers of smoking and SHSe, smoking cessation, smoke-free home, PM25 feedback, written materials. CG: written materials only | Minimal intervention:  The control group received  only a brief educational leaflet on  the hazards of SHS | Used Sidepak to compare the quality of indoor air with outdoor air - providing personal Pm2.5 feedback. | **Child hair nicotine concentration:** 17% lower in IG than CG although not significant (*p* = 0.239). Significantly decreased in IG from baseline to follow up (0.30 ng/mg to 0.23 ng/mg; *p* = 0.024). According to the smokers, the increase in the proportion of households with smoking restrictions at follow-up was statistically significant only in the intervention group.  **Maternal knowledge of SHSe and smoking hazards:** IG: From 9.5 at baseline to 11.3 at follow up. CG: From 9.8 to 10.5. 10% higher in IG than CG after controlling for baseline score (*p* = 0.006). |
| Zakarian et al.  2004. USA [51] | To evaluate the effectiveness of a behavioural counselling program for reducing child SHSe. | 150 mothers of children aged less than 4 years attending a well-child community clinic. Most mothers were White, not employed, low education. Approximately 40% were single parents. | RCT | Behavioural counselling including contracting to reduce SHSe, problem solving, goal setting and self-monitoring. | Measures only control group. | No feedback mentioned. | **Maternal report of child SHSe (number of maternal cigarettes child exposed to per week:** Declined for baseline to 6 months post-test for both groups (IG: 18.89 at baseline to 5.41 at 12 months. CG: 13.25 at baseline to 5.23 at 12 months) (*p* < 0.001). Data presented in graph difficult to report exact results. Priest *et al.* (2008) reported data. Total exposure to cigarettes/week (IG 53.2 at baseline to 21.99 at 12 months. CG: l 54.48 at baseline to 18.22 at 12 months) (*p* < 0.001). No significant group x time differences. Number of counselling sessions completed was not a significant covariate.  **Children’s urinary cotinine concentration:** No significant change over time in either group. No significant group x time or group differences. |
| Emmons et al.  2001. USA [52] | Outcome evaluation of project KISS (Keep  Infants Safe From Smoke) which is a motivational intervention. | 291 smoking low-income parent/caregivers Children younger than 3 years. | RCT | Choice, personal responsibility for change, self-efficacy, feedback on CO level. Tailored to interest in quitting smoking or reducing SHSe. Goal setting. | Self-help comparison condition. Received a copy of a smoking cessation manual, passive smoke reduction tip sheet, and the resource guide in the mail. | Feedback from baseline household air nicotine assessments and assessment of the participant’s carbon monoxide level was provided as part of the intervention. | **Nicotine levels in household:** significant time-by-treatment effect (*F* (2406) = 4.80, *p* < 0.01). IG: Levels at 3 & 6 months significantly lower than baseline (*F* (2200) = 4.36; *p* < 0.5). SH group nicotine levels not significantly different from baseline. |
| Baheiraei et al.  2011. Iran [53] | To assess whether counselling both mother and father reduces infant SHSe. | 130 parents of health infants  (<12 months) with at least one parent smoker. Families from predominately lower SES. | RCT | MI used and counselling sessions based on “Smoke-free children” method (Arborelius & Bremberg, 2001; Fossum et al., 2004; Greenberg et al., 1994). | Usual care including usual health care visits for checking infants’ growth and development milestones. | Urinary cotinine levels taken but no mention of providing these levels as feedback to participants. | **Urine cotinine:** Decreased for both groups but significantly decreased in IG (Baseline: IG 48.72 vs. CG 40.83; 3 months IG: 28.68 vs. CG 3.32). p = 0.029).  **Total daily cigarette consumption:** Greater decrease in presence of child in IG (median = 0, interquartile range: 0, 2.71) than CG (median = 1, interquartile range: 0, 3.21) at the 3 month follow up (one tailed p, 0.3). No significant correlation between cigarettes consumed and reported level of SHSe. **Home and car smoking bans:** Increase in both IG & CG, but not significant in CG. Statistically significant between groups (p = 0.49). |
| Wilson et al.  2013.  Scotland [54] | To investigate feasibility of an intervention  (REFRESH) to reduce SHSe for children  in their homes. | 59/1693 smoking mothers with at least one child younger than 6 years.  Maternal age M = 30 years; child age  M = 3.5 years (range 1.2–5.7 years). | Pilot RCT | 24 h measure on home air quality PM2.5 (particulate matter) & motivational interview. | Control group received same intervention with the exclusion of air quality feedback at visit 2. | Personalised air quality feedback was provided to parents at visit 2 (midway through intervention). | **Difference in PM2.5 from visit 2 to visit 4:** IG achieved a greater reduction between baseline and final PM levels (p <.05).  **Peak concentration of PM2.5**: IG 67 vs. CG 148 (p = 0.006).  **The percentage of time when household PM2.5 concentrations exceeded 35** μ/m3: IG 0.49 vs. CG 3.6 (p = 0.017).  **Children’s salivary cotinine:** No significant difference. |
| Conway et al.  2004. USA [59] | To evaluate the effectiveness of a lay delivered intervention to reduce SHS exposure in Latino children. | 143 Latino parent-child pairs. Child age 1–9 years (M = 4 years). | RCT | Problem solving aimed at lowering  child SHS in the household | Measurement only controls. (participated in surveys but received no intervention) | No feedback reported. | **Child hair nicotine (log ng/mg):** Baseline (IG: 0.25 *vs.* CG 0.23), post intervention (IG: 0.17 *vs.* CG: 0.19, 3 months (IG: 0.28 *vs.* CG 0.32), 12 months: (IG: 0.23 *vs.* CG: 0.23). No significant differences between groups over time.  **Child hair cotinine (log ng/mg):** Baseline (IG 0.05 *vs.* CG 0.05), post intervention (IG 0.03 *vs.* CG 0.03), 3 months (IG 0.04 *vs.* CG 0.04), 12 month (IG 0.02 *vs.* CF 0.04). No significant differences between groups, but time effect detected (*p* < 0.001).  **Parent report of number of cigarettes child exposed to in household over one month:** Baseline (IG 1.75 *vs.* CG 1.85), post intervention  (IG 1.42 *vs.* CG 1.62), 3 months (IG: 1.27 *vs.* CG 1.44), 12 months (IG: 1.06 *vs.* CG 1.27). No significant difference between groups, trending toward significance over time (*p* = 0.048).  **Confirmed reduction (dichotomous variable based on parent report and child hair biomarkers:** Not significant. |
