# Supplementary material for: Development of a Smoke-Free Homes Intervention for Parents: An Intervention Mapping Approach
Source: Health Psychol Bull. Author manuscript; Available in PMC 2020 Apr 24. (PMC7182446; doi:10.5334/hpb.20)
Supplement: Supplementary file 4 [file EMS86099-supplement-Supplementary_file_4.docx]

**Review of Successful Interventions That Have Used Objectively Assessed Feedback to Motivate Health Behaviour Change**

**Objective:** Our objective was to conduct a rapid review of published papers to investigate the effectiveness of providing objective feedback to improve/change health behaviours. The three health behaviours chosen to review included: smoking cessation; medication adherence (MA), and physical activity (PA). These specific health behaviours were selected as objective feedback measures have been established in previous research including; CO2 assessments, electronic MA monitoring and pedometer use, respectively. The purpose of the current rapid review is to extract important and/or successful components of feedback interventions, which could in turn, guide the development of a practical intervention to reduce second-hand smoke in homes using an air-quality feedback instrument.

**Eligibility:** The eligibility criteria used in the current review included: 1/ empirical study reports of interventions aimed at promoting a specific health behaviour through the use of objective feedback, 2/ study reports which found the intervention to be effective, 3/ RCT’s or Pilot RCT’s, 4/ study reports that provided a comprehensive description of the intervention. Included papers were published between 2001 and 2015 in peer reviewed journals to ensure a focus on the most recent research in the topic. Papers were not considered if they were not written in English.

**Information Sources:** Cochrane Database of Systematic Reviews was used for the smoking cessation element of the current review. Search terms included carbon monoxide and feedback. A review by Bize et al. [1] was selected to extract adequate studies, as their subject focus was relevant to the current rapid review: “Biomedical Risk Assessment as an Aid for Smoking Cessation (Review)”. Bize et al. [1] found that out of the fifteen trials that were included in the review, only two feedback interventions were found to be effective in increasing smoking cessation rates. Only one category of feedback type (smoking related harm) had a long term effect on smoking cessation, whereas biomarkers of smoking exposure including cotinine and carbon monoxide, and smoking related disease risk were not found to have a significant effect. The two successful feedback intervention studies identified by Bize and colleagues [1] included in the current review are: Bovet et al. [2]; Parkes et al. [3].

For the medication adherence element of the current review, Cochrane Database of Systematic Reviews was searched in an attempt to find systematic reviews specifically investigating trials which used objective feedback as part of the intervention, but none were found. Thereafter, we conducted a manual search on Cochrane for individual trials using the search terms: "medication adherence" OR "adherence enhancing" AND "feedback intervention.” Two relevant studies were identified: “Electronic monitoring-based counselling to enhance adherence among HIV-infected patients: A randomized controlled trial” by De Bruin et al. [4] and “Randomized Pilot Study of a Behavioral Feedback Intervention to Improve Medication Adherence in Older Adults with Hypertension” by Ruppar [5].

Although two study protocols have been published proposing pedometer-based interventions to increase physical activity (Harris et al. [6]; Pillay et al. [7]), no actual effective studies were found using Cochrane Database of Systematic Reviews, but instead the instruments (pedometers) were used as a measurement of outcome rather than a feedback intervention, for example Dishman et al. [8]. We then conducted a manual search on Cochrane for individual trials using the search terms: “physical activity AND feedback” and one relevant study was identified: “The effects of a lifestyle physical activity counselling program with feedback of a pedometer during pulmonary rehabilitation in patients with COPD: A pilot study” by De Blok et al. [9]. A manual search was then conducted on the University of Aberdeen’s Primo Central search engine using the search terms: “physical activity AND feedback intervention”. The identified study from the Primo search was: “The impact of different degrees of feedback on physical activity levels: A 4-week intervention study” by Van Hoye et al. [10].

**Summaries of Included Papers**

Brief summaries of the included papers have been provided. For more detail regarding design and outcomes, please see Table 2, Appendix A.

**Bovet et al. (2002). The Seychelles [2]:**

The intervention involved submitting smokers to ultrasonography of their carotid and femoral arteries. Patients who had an identified plaque then received two personal ultrasound photographs of the plaque with a relevant explanation provided.

The study suggests that providing smokers with photographs of ultrasonic images demonstrating their own atherosclerotic lesions together with relevant explanations, have better smoking cessation success rates than smoking cessation counselling alone. Since ultrasonography is used increasingly often in clinical practice for cardiovascular risk stratification, this can provide an additional opportunity and means to deter smokers from smoking, and thus making it a cost-effective intervention.

Making smokers aware of their own plaques does not necessitate a smoker’s prior decision to quit smoking which means that it is not limited to only treatment-seeking smokers, therefore implying that a large proportion of smokers in the general population could potentially benefit from this intervention.

Presenting the personalised photographs to smokers may have increased motivation to quit by removing the perception of smoking as a hypothetical and remote health hazard. The author’s suggest that demonstrating atherosclerotic plaques to smokers could facilitate a transition of early stages of behaviour change (pre-contemplation, contemplation) to action stages of changes, as per the transtheoretical model of behaviour change.

**Parkes et al. (2008). UK [3]:**

The intervention involved providing participants with results of their lung function assessment in the form of lung age with a graphic display. The graphs (feedback) were used as a visual aid to explain how the lung function normally reduces gradually with age and that smoking can damage lungs as if they are ageing more rapidly than normal. If lung age was greater than chronological age, participants were provided with “lung age” in years. The study demonstrates that individualised feedback of lung age along with health risk education and methods of quitting is effective in promoting smoking cessation.

Severity of lung damage was not found to be related to successful quitting which suggests that all smokers have the potential to benefit from this intervention, whether or not they have a deterioration of lung function. The author’s suggest that the behaviour change is more successful when information is presented in an understandable and visual way, irrespective of whether the information given is positive or negative, than when people are given information that cannot be understood easily.

Although a full economic evaluation was not conducted, the authors suggest that the intervention has comparable effectiveness to, and potentially less expensive than, other currently available treatments on the NHS.

**De Bruin et al. (2010). Amsterdam [4]:**

A minimal intervention (adherence sustaining) comprising of MEMS report feedback, reinforcement and a brief discussion to tackle adherence difficulties, was delivered to HIV-patients if their baseline medication adherence was at least 95%. Patients with less than 95% baseline adherence were subjected to an intense intervention (adherence improving) which involved counselling sessions with the HIV-nurse who followed a 9-point intervention protocol.

The AIMS intervention was effective for improving medication adherence, even though the control group already received high quality standard care. However, the treatment effect on adherence only occurred among the patients with adherence below 95% at baseline (adherence improving group), therefore suggesting that the minimal intervention may not have an additional value over high-quality standard care. Consequently, the authors suggest that efforts should be targeted primarily at non-adherent patients.

The use of detailed MEMS-feedback in the intervention was claimed to be a core element of AIMS and was appreciated by both patients and health care providers. The CG participants received feedback about their viral load and CD4 count (health status) at their routine clinic appointments, therefore suggesting that it is specifically behavioural feedback (IG) that has an effect on people’s health behaviour rather than health status feedback.

**Ruppar (2010). USA [5]:**

The eight-week behavioural feedback intervention involved providing participants with; biweekly medication adherence and blood pressure feedback which was obtained through electronic monitoring, habit counselling, medication and disease education, a medication instruction card, and an electronic medication bottle cap with a digital display that provided daily adherence feedback throughout the intervention period. This study demonstrates the potential effectiveness of this feedback-based antihypertensive MA intervention protocol for older adults.

The author’s claim that most participants reported a sense of benefit from the intervention, particularly from the verbal and visual adherence feedback and BP feedback components. Many intervention participants would compare their adherence and BP levels with those from their prior visit to gauge their progress and the need for further attention to their medication-taking behaviour. MEMS data at feedback visits also allowed the interventionist to analyse participants’ dosing times and suggest modifications to daily routines to facilitate improved adherence by changing medication administration times or by linking medication administration to a different routine behaviour.

The authors also state that the ability to link study outcomes to the conceptual framework was limited and that additional concepts were needed to be added, including; illness interpretations, medication beliefs, and barriers to obtaining medications.

The final 2 weeks of the 6-week screening period were used to determine baseline MA for study eligibility. This run-in period was designed to eliminate the MEMS intervention effect of about 1 month seen in other populations. This is an important factor to consider when designing/developing other types of feedback interventions as one could have more confidence in the cause/effect relationship.

**De Blok et al. (2006). Netherlands [9]:**

The intervention was a lifestyle physical activity counselling programme with feedback of a pedometer, in addition to a regular pulmonary rehabilitation programme. The authors of this study claim that the intervention in addition to rehabilitation effects, results in a clinically relevant increase in daily steps.

Using a pedometer as a feedback tool to change behaviour is increasing in popularity due to the pedometer being: affordable, user-friendly output, and measurement accuracy, according to De Blok et al. (2006). These three factors should therefore be considered when designing/developing other behavioural feedback interventions.

**Van Hoye et al. (2015). Belgium [10].**

The intervention involved a four-arm design: a Minimal Intervention Group (MIG-no feedback), Pedometer Group (PG-feedback on steps taken), Display Group (DG-feedback on steps, minutes of moderate to vigorous physical activity and energy expenditure) and a Coaching Group (CoachG-same as DG with need-supportive coaching).

This study revealed a higher adjusted step count during the first week of the intervention for the PG compared with the MIG. However, no significant differences were found between these two groups after one week of the intervention. The authors suggest that the initial increase in daily steps may be caused by the issue of reactivity to self-monitoring devices, concluding that if pedometers are to be used as an intervention tool, further strategies need to be used to sustain the motivational impact of pedometer use after the first week.

No differences in the PA behaviour between the two feedback modalities (PG and DG) were found across time. Therefore, in terms of self-monitoring, using a highly technological device with detailed feedback seems of no greater value than a low-cost pedometer.

The results demonstrated that having a weekly meeting with a coach leads to a higher and sustained increase in PA compared with using only a self-monitoring device. However, the SWA data was exported and used to objectively and graphically present the PA behaviour of the participants during the coaching sessions, therefore making it possible that the graphical display of information independently contributed to the increase in PA. The SWA display also served as a tool to personalize the exercise targets and to provide motivational messages when the individualized targets were met. Overall, the study provides evidence that minimal contact interventions have the capacity to produce behaviour change.

Table 1: Intervention Characteristics

| Author | Content | Delivery Personnel | Method of communication | Intensity/ Complexity | Conceptual Framework |
| --- | --- | --- | --- | --- | --- |
| Bovet et al. (2002). [2] | Smokers in all three groups received 10-min smoking cessation counselling by a physician. Those randomised to the ultrasonography also received two ultrasound photographs of a plaque with a relevant explanation provided (group C). Participants who did not have identified plaque therefore did not receive photographs (Group B). | Examiner/ Physician | Face to face, phone. | 1 x ultrasonography session + 2 telephone interviews. | None mentioned although relates findings to transtheoretical model. |
| Parkes et al. (2008). [3] | All participants underwent standard measurements of lung function. All participants were strongly encouraged to give up smoking and advised how to access local NHS smoking cessation clinics. Participants in the IG were given their results verbally, immediately after randomisation, in the form of “lung age” with a graphic display. The graphs were used as a visual aid to explain how the lung function normally reduces gradually with age and that smoking can damage lungs as if they are ageing more rapidly than normal. In the IG, if lung age was greater than chronological age, participants were provided with “lung age” in years. The CG were not given their results. Participants underwent follow-up examination with repeat spirometry after 12 months. Self-reported quitters had carbon monoxide breath testing immediately for confirmation of smoking cessation. | Practice nurses, healthcare assistant, GP. | Face to face, written. | 2 x spirometry assessments + 2 x survey interviews + 1 x salivary cotinine measurement. | Transtheoretical model. |
| De Bruin et al. (2010). [4]. | Patients in IG had their adherence monitored and recorded from MEMS caps data. If patients achieved >95% adherence, they were shown their data in graph form and encouraged to maintain adherence and discuss any barriers they may have. Patients in the IG who did not achieve a min of 95% adherence had a discussion/session with their HIV-nurse, who followed a 9-point intervention protocol including: providing tailored and practical adherence knowledge, discussion of reasons for non-adherence, desired status compared with actual status (feedback), problem solving, action planning, self-monitoring, re-evaluation, maintenance planning (intensive intervention). | HIV-nurses. | Face to face, written. | 3 x clinic appointments. 1 x screening assessment. | Author’s own Behavioural Model for Medication Adherence, based on Theory of Planned Behaviour (TPB). |
| Ruppar (2010). [5]. | The MA intervention consisted of 5 components: MA feedback, hypertension feedback, medication-taking skills, habit adjustment, and succinct medication and disease information delivered to the participant over an 8-week period. At each home visit, participants’ MEMS caps data was fed back using graphical displays. Participant’s resting BP was also measured and recorded in participant’s diaries. The interventionist discussed the degree of change in the participants’ BP and how it could be positively impacted by improvements in MA. | Gerontological advanced practice nurse (T.M.R.). | Face to face, written. | 4 x home visits over 8 weeks. 1 x screening assessment. | The intervention approach for this study was guided  by a framework adapted from the Self-regulation  Model of Leventhal et al. although the study uses MA and BP feedback as a surrogate for the perceived symptoms that would normally help individual’s self-regulate medication-taking behaviour. |
| De Blok et al. (2006). [9]. | The first counselling session was carried out 2 weeks prior to rehabilitation and dealt with motivation for increasing physical activity. In this session, the pedometer with user instructions were given to the patient. The second counselling session was carried out in week 1 of the rehabilitation and dealt with goal-setting. The third counselling session was carried out in week 5 of the rehabilitation and dealt with shifting boundaries. Mean steps/day were evaluated and patients were asked to set a goal for seeking their maximal physical activity limit once (measured in numbers of steps). The fourth counselling session was carried out in week 7 of the rehabilitation and dealt with consolidation of physical activity behaviour. Patients were asked to set a goal for their personal physical activity norm, which should be between their mean steps/day until then and their maximal number of steps. | Exercise counselling trained physical therapists. | Face to face. | 4 x individually exercise counselling sessions lasting approximately 30 minutes each. | Motivational Interviewing principles. |
| Van Hoye et al. (2015). [10]. | Four week intervention period. MIG group received no feedback during the 4-week intervention period which involved a meeting with the test instructor before entering the 4-week intervention period. During this meeting, their objectively measured PA level was discussed and compared with national  PA recommendations. PG group received information on their daily step count during the 4-week intervention by using a pedometer and was given a step diary to write down their daily steps. They were instructed to take at least 10,000 steps a day. DG group received feedback on steps, minutes of MVPA per day and total EE per day during the intervention by means of a real-time wrist watch SWA display and were required to complete a PA diary daily with information on step counts attained, minutes of MVPA, total kcal burned and type of activity performed. They also received a list of possible activities they could perform to increase their energy expenditure. CoachG group also received the SWA display and PA diary. The daily targets on steps, minutes of PA and total EE were weekly increased so that at the start of the intervention targets were easily met and by the end of the intervention, more effort was needed. Additionally these individuals had weekly meetings with a Personal Coach to discuss their PA behaviour (as written down in their PA diary) and the efforts that were made to change that behaviour. Furthermore, the graphical display of information was used to help foster understanding of the PA behaviour. | The coach was an academic master in Physical Education and Movement Sciences. | Face to face. | 4 x weekly coaching sessions lasting between 30 and 45 minutes. Intervention period = four weeks. | The personalized feedback provided as part of the intervention (CoachG) was based on the Self  Determination Theory (SDT). |

**Synthesis:**

A narrative synthesis has been conducted by extracting relevant information from the brief summaries, intervention characteristics (Table 1) and the overall review table (Table 2; Appendix A).

**Intervention Delivery and Complexity:**

The majority of the six interventions were delivered by health professionals (2, 3, 4, 5, and 9), whereby most of them were nurses (3, 4, and 5). All of the interventions involved face-to-face communication. The maximum number of intervention sessions that participants were required to attend was four (5, 9, 10) and the minimum was one (2).

**Feedback Format:**

All of the interventions reviewed provided some form of personalised feedback. The most common feedback format used was graphical displays (3, 4, and 5). Photographs were used in one intervention (2), a real-time measurement in another (10), and one intervention did not specifically state the format of the feedback (9).

**Feedback Type:**

Two thirds of the reviewed studies provided participants with behavioural feedback (4, 5, 9, and 10) and one third provided health status feedback (2, 3) as a way to motivate behaviour change. Although all of these studies found positive effects, one study suggests that behavioural feedback (intervention group) serves as a core component to behaviour change, compared to health status feedback (control group). Subsequently, health status feedback, (e.g., spirometry assessment) may not provide accurate data of changed behaviour (e.g., smoking cessation), and therefore still has to be confirmed through additional means (e.g., carbon monoxide breath testing), as suggested by study 3.

**Other strategies Used:**

Two of the six studies did not use any other strategy other than feedback (2, 3). Three of the remaining studies used counselling sessions appropriate to the targeted health behaviour (4, 5, and 9) and one study used need-supportive coaching sessions (10).

**Theoretical Frameworks:**

There was not one particular theoretical framework which dominated the six reviewed studies. Each study adopted a different theory/conceptual framework to guide the intervention: Transtheoretical model (3), Theory of Planned Behaviour (4), Self-regulation model (5), Motivational Interviewing principles (9), Self-determination theory (10, study 2 did not state any theory or concept as the basis for intervention). Nevertheless, common adopted concepts were explicitly mentioned, the most common being: goal setting (4, 9, 10), education provision (4, 5, 9), self-monitoring (4, 10), and problem solving (overcoming barriers; 4, 10).

**What Have We Learned?**

- Health professionals are the desired delivery personnel among successful feedback intervention, especially nurses.
- Face-to-face communication is the consistent approach used by researchers who have used feedback interventions and found positive effects.
- Multiple attendance to intervention sessions is not required as the maximum number needed for the successful intervention was four sessions.
- Using graphical displays is the most common feedback format used.
- Presenting personalised feedback to individuals may increase motivation to change their health behaviour by removing the perception of the alternative health risks as hypothetical.
- Providing behavioural feedback as opposed to health status feedback may be more motivational for individuals to change their health behaviour.
- Counselling/coaching sessions is the preferred strategy to use with feedback interventions in order to achieve positive behaviour change.
- The most common concepts to use alongside feedback interventions are: goal setting, education provision, self-monitoring and problem solving.
- The issue of reactivity to self-monitoring devices should be considered when developing behavioural feedback interventions, by incorporating an appropriate run-in period.
- Important feedback components include affordability, user-friendly output and measurement accuracy.

These learning points should be taken into consideration when planning/developing interventions to reduce SHS in the home by parents.

References

1. Bize, R., Burnard, B., Mueller, Y., Rĕge-Walther, M.,Camain, J. Y., & Cornuz, J. (2012). Biomedical risk assessment as an aid for smoking cessation (Review). *The Cochrane Collaboration, 12*.
2. Bovet, P., Perret, F., Cornuz, J., Quilindo, J., & Paccaud, F. (2002). Improved smoking cessation in smokers given ultrasound photographs of their own Atherosclerotic plaques. *Preventive Medicine. 34*, 215-220. doi:[10.1006/pmed.2001.0976](http://dx.doi.org/10.1093%2Fntr%2Fntp148)
3. Parkes, G., Greenhalgh, T., Griffin, M., & Dent, R. (2008). Effect on smoking quit rate of telling patients their lung age: The step2quit randomised controlled trial. *BMJ, 336*(7644), 598-600.
4. De Bruin, M., Hospers, H. J., van Breukelen, G. J. P., Kok, G., Koevoets, W. M., & Prins, J. M. (2010). Electronic monitoring-based counseling to enhance adherence among HIV-infected patients: A randomized controlled trial. *Health Psychology, 29*(4), 421-428.
5. Ruppar, T. M. (2010). Randomized pilot study of a behavioural feedback intervention to improve medication adherence in older adults with hypertension. Journal of Cardiovascular Nursing, 25(6), 470-479. doi:[10.1097/JCN.0b013e3181d5f9c5](http://dx.doi.org/10.1093%2Fntr%2Fntp148)
6. Harris, T., Kerry, S., Victor, C., Ekelund, U.,Woodcock, A., Lliffe, S., … Cook, D. (2013). Randomised controlled trial of a complex intervention by primary care nurses to increase walking in patients aged 60-74 years: protocol of the PACE-Lift (Pedometer Accelerometer Consultation Evaluation – Lift) trial. *BMC Public Health, 13:5*. doi:10.1186/1471-2458-13-5
7. Pillay, J., Kolbe-Alexander, T. L., Proper, K. I., Van Mechelen, W., & Lambert, E. V. (2012). Steps that count! : The development of a pedometer-based health promotion intervention in an employed, health insured South African population. *BMC Public Health, 12:880*. doi:10.1186/1471-2458-12-880
8. Dishman, R. K., DeJoy, D. M., Wilson, M. G., & Vandenberg, R. J. (2009). Move to improve: A randomized workplace trial to increase physical activity. *American Journal of Preventive Medicine, 36*(2), 133-141.
9. De Blok, B. M. J., de Greef, M. H. G., ten Hacken, N. H. T., Sprenger, S. R., Postema, K., & Wempe , J. B. (2006). The effects of a lifestyle physical activity counselling program with feedback of a pedometer during pulmonary rehabilitation in patients with COPD: A pilot study. *Patient Education and Counseling, 61*, 48-55. doi:[10.1016/j.pec.2005.02.005](http://dx.doi.org/10.1093%2Fntr%2Fntp148)
10. Van Hoye, K., Boen, F., & Lefevre, J. (2015). The impact of different degrees of feedback on physical activity levels: A 4-week intervention study. *International Journal of Environmental Research and Public Health, 12*, 6561-6581. doi:[10.3390](http://dx.doi.org/10.1093%2Fntr%2Fntp148)/ijerph120606561

Appendix A

Table 2. General Components and Results of Studies Which have used objective feedback to improve health behaviours.

| Reference | Objective | Sample | Design | Intervention | Comparator | How FB was embedded | Results/Outcomes |
| --- | --- | --- | --- | --- | --- | --- | --- |
| Bovet et al. (2002). [2] The Seychelles. | SMOKING CESSATION. To examine whether making smokers aware that they had developed peripheral atherosclerosis would improve smoking cessation. | 153 adults who were current smokers. | RCT | Smokers submitted to ultrasonography of their carotid and femoral arteries and given personal ultrasound photographs of a plaque and a brief explanation. | 3 groups in total: Group A = control group and did not receive ultrasonography or photograph. Group B = received ultrasonography but did not have plaques, so did not receive a photograph. Group C = IG. | Participants in Group C received photograph of their arterial plaque. | Quit rates, measured 6 months after the intervention, were respectively, 6.3, 5.0, and 22.2% in Groups A, B and C. Quit rates were higher in smokers submitted to ultrasonography (B + C vs A; *p* = .031) and in those receiving photographs (C vs A + B; *p* = .003. Smoking cessation was independently associated with intervention C. |
| Parkes et al. (2008). [3] UK | SMOKING CESSATION. To evaluate the impact of telling patients their estimated spirometric lung age as an incentive to quit smoking. | 561 current smokers aged ≥ 35 years. | RCT | IG given spirometric assessment of lung function and received results in terms of lung age. | CG given spirometric assessment of lung function and received a raw figure for forced expiratory volume at one second. | IG given personalised results verbally with a graphical display. | Independently verified quit rates at 12 months in the IG and CG, respectively, were 13.6% and 6.4% (difference 7.2%, P=0.005, 95% confidence interval 2.2% to 12.1%; number needed to treat 14). People with worse spirometric lung age were no more likely to have quit than those with normal lung age in either group. |
| De Bruin et al. (2010). [4] Amsterdam. | MEDICATION ADHERENCE. To investigate the effectiveness of an adherence intervention (AIMS) designed to fit with HIV-clinics’ routine care practices. | 133 HIV-infected patients. Mean age = 48 years. | RCT | Electronic monitoring-based counselling.  Data from MEMS cap provided to patient and behaviour reinforced if adherence was adequate (minimal intervention). If adherence was suboptimal, a 9-step intervention protocol was initiated. | Control group = standard care provided, including education, use of helpful devices, problems and solution discussion, and feedback provision about their viral load and CD4 count.  IG included 2 sub-groups = (intensive intervention, <95% adherence at baseline = adherence improving sub-group) and (minimal intervention, > 95% adherence at baseline = adherence sustaining group). | Antiretroviral medication adherence measured electronically with Medication Event Monitoring System (MEMS) caps. MEMS data were used to measure the outcome of the intervention, but also served as feedback to participants during the intervention. | Adherence improved significantly in the complete intervention sample. Subgroup analyses showed that this effect was caused by participants scoring <95% at baseline (mean difference = 15.20%; *p* < .001). These effects remained stable during follow-up. The number of patients with an undetectable viral load increased in the intervention group compared to the control group (OR = 2.96, *p* < .05). |
| Ruppar (2010). [5]. USA. | MEDICATION ADHERENCE: To pilot test a new behavioural feedback intervention to improve medication adherence (MA) among older adults with hypertension. | Fifteen participants (median age,  71 years; 73% female). | Pilot RCT. | 8-week MA intervention consisting of MA  feedback, BP feedback, habit modification, and medication and hypertension education in a sample of older adults who were non-adherent to their antihypertensive medication regimens. | Control group = usual-care-group: participants were seen by the investigator only for data collection at 12 and 20 weeks. | At the home visits, the participants’ MEMS cap data were downloaded to a laptop computer. Participants were informed of their adherence rate since the last visit and were shown a graphical display of their MA behaviour to date. BP measured at each visit and recorded in diaries. | At the end of the intervention, the treatment group had better antihypertensive MA than did the control group (median MA: 100% vs 27.3%, U = 5.00, P = .013). Systolic BP improved slightly in the intervention group during the study and was significantly different at week 12 (median systolic BP: 130 vs 152 mm Hg; U = 4.50, P = .008). Diastolic BP was largely unchanged over the course of the study. |
| De Blok et al. (2006). [9] Netherlands. | PHYSICAL ACTIVITY: To study the effects of a lifestyle physical activity counselling programme with feedback of a pedometer during pulmonary rehabilitation. | Twenty-one chronic obstructive pulmonary disease (COPD) patients. (43% female, mean age > 60 years). | Pilot RCT. | A lifestyle physical activity counselling program with feedback of a pedometer in addition to the regular pulmonary rehabilitation program. IG group wore the pedometer for 10 weeks (1 week prior to rehabilitation and 9  weeks during rehabilitation) | Control group = received same standard rehabilitation programme as IG which contained exercise training, dietary intervention and psycho-educational modules. CG wore the pedometer 1 week prior to the rehabilitation and 1 week during week 9 of their rehabilitation. | IG wore a pedometer which was used as a motivational and a feedback tool. Feedback of amount of steps taken was analysed at session 3 (out of 4) and goals were set for the next session. Daily recording of pedometer steps provided daily feedback to participant. | The experimental group showed an increase of 1430 steps/day (+69% from baseline), whereas the control group showed an increase of 455 steps/day (+19%) (p = 0.11 for group _ time interaction). The effect of the lifestyle physical activity  counselling program with feedback of a pedometer was not statistically significant, but the between-groups effect size  was large (>0.80) |
| Van Hoye et al. (2015). [10] Belgium. | PHYSICAL ACTIVITY: To investigate the effectiveness of different degrees of feedback and the added value of coaching  In promoting PA behavioural change. | 227 self-reported inactive adult participants. (124 female; | RCT. | Four intervention arms  varying in different degrees of feedback: Minimal  Intervention Group (MIG-no feedback). Pedometer Group (PG-feedback on steps taken),  Display Group (DG-feedback on steps, minutes of moderate to vigorous physical activity and energy expenditure) or Coaching Group (CoachG-same as DG with need-supportive coaching). | Minimal  Intervention Group (MIG-no feedback) | During the intervention period, all groups with the exception of the MIG, received real-time feedback about their activity behaviour. The PG received feedback on the number of steps by means of a waist-mounted device. The DG and CoachG used the SenseWear Armband (SWA) display which provided feedback on both the activity behaviour (i.e., daily steps, minutes of MVPA) as well as the behavioural outcome (i.e., daily EE). | No significant Group × Time interaction effect for the different PA variables between the MIG and PG. Also no differences emerged between PG and DG. As hypothesized, CoachG had higher PA values throughout the intervention compared with DG. |
