# Supplementary material for: Development of a Smoke-Free Homes Intervention for Parents: An Intervention Mapping Approach
Source: Health Psychol Bull. Author manuscript; Available in PMC 2020 Apr 24. (PMC7182446; doi:10.5334/hpb.20)
Supplement: Supplementary file 5 [file EMS86099-supplement-Supplementary_file_5.docx]

**Review of Qualitative data: SHS exposure (SHSe) in the home**

Information regarding relevant determinants of outdoor smoking was extracted from qualitative research which members of the current research team have been involved with. All of the included studies took place in Scotland with individuals who smoked in the home and/or who lived with people who smoked in the home, therefore making the results relevant to “our” project aims.

The main objective of conducting the current review of these qualitative findings was to establish: the current level of awareness/knowledge of SHSe; strategies/enablers to reduce SHSe in the home, and barriers to reducing SHSe in the home. Each of the aforementioned components are discussed as follows:

**Awareness/knowledge of SHSe:**

The majority of participants reported that they were aware of the risks of SHSe. However the extent of SHSe awareness varied between participants, with a few even rejecting the evidence of such risks. Although parents claimed that they were aware of the SHSe risks, they did not always appear to be aware that even when risk reduction strategies were adopted (e.g. smoking at the kitchen window) that the level of smoke in their home was above the recommended safe level. These results suggest that individuals do not always automatically make the connection between the risks of SHSe and their smoking behaviour in the home or the extent of this link, even though most do make the connection between smoking in the car and SHS risks.

In most cases, it does not appear to be the fact that individuals are submissive to the risk knowledge of SHSe, as many participants stated that they knew that children were more vulnerable and that there was a desire to protect their children’s health. Therefore, it is imperative that smokefree homes interventions are developed to include an element of tailored information regarding individual smoking behaviour and how this links to SHSe risks. This type of intervention will enhance/help parents to accomplish the already expressed desire to protect their child form SHSe. Therefore, providing individuals with air quality feedback alongside information regarding how this can be translated into SHSe risks would be ideal.

**Strategies/enablers to reduce SHSe in the home:**

A common theme of strategies to reduce children’s SHS exposure in homes involved spatial and dispersal measures including: smoking at a window/outside door; only smoking in certain rooms (usually the kitchen); throwing ashtrays away or moving ashtrays into the kitchen; only smoking indoors when the child/children were absent. Therefore parents do actively try to reduce SHSe in the home, although the strategies may not be as effective, which again supports the idea that parents’ knowledge of their own smoking behaviour and the risks of SHS has to be increased.

There appears to be two main categories of enablers to reduce SHSe in the homes; practical enablers and internal motivational enablers. Practical enablers include: having access to outdoor space (garden) and having the presence of a partner to look after the child. Internal motivational factors include: social norms, e.g. the desire to be seen as behaving in morally and socially acceptable ways (being a good parent), concerns about children’s health and becoming future smokers (role modelling), and pressure from children not to smoke. Other factors which were mentioned in some studies also included aesthetic factors as a motivator to reduce smoking in the home.

These mutual strategies and enablers should be considered when developing a smokefree homes intervention. They could be included as part of a counselling programme as ways of supporting people to change their smoking behaviour. Nevertheless, it must also be noted that many of these factors may be out-with an individual’s control, in particular the practical enablers.

**Barriers to reducing SHSe in the home:**

Living circumstances have a massive impact on the reduction of SHSe in the home such as, living in a block of flats and therefore unable to leave the child/children alone to go outside to smoke, and also living in small homes means that there is less distance between smokers and children. These barriers are particularly true of disadvantaged families.

Social norms act as barriers to reducing SHSe in the homes, similar to how they act as enablers. For example, the acceptability of smoking in the home among family and friends and alternatively feeling reluctant to have to ask others to smoke outside can have a detrimental effect on people’s plans to reduce SHSe in their homes.

Another important factor that appears to “encourage” parents to smoke in the home is the feelings of being stigmatised as a bad parent because they are smoking which ultimately discourages people from smoking in public, thus they are more inclined to smoke indoors. If parents therefore care about how others view them as parents, future intervention should address this issue in a supportive manner by reinforcing that they would be doing more for the child’s health if they smoked outdoors rather than indoors, i.e. make parents feel good about making positive steps to reduce SHSe in the home.

Other barriers to reducing SHSe in the home include: being lazy, the weather, beliefs and knowledge of the effectiveness of SHS reduction, increasing mobility of children, the need to smoke, increased moodiness, stressful life events and weight gain.

**Conclusion:**

Much of the identified determinants of outdoor smoking are out-with the reach of any single intervention and may be more a case for public policy to address, such as living circumstances. Nevertheless, there are still many factors that could be addressed by a smokefree intervention. The main factors which should be considered when developing a smokefree home intervention based on past qualitative findings are; Education, Tailored information regarding individual smoking behaviour and how this links to SHS risks, Counselling/coaching to encourage positive social norms (good parenting), how to tackle negative social norms and addressing negative feelings of stigmatisation, and Smoking cessation referrals. For specific details of each study, please see Table 1, Appendix A.

**References:**

1. Rowa-Dewar, N., Amos, A., & Cunningham-Burley, S. (2014). Children’s perspectives on how parents protect them from secondhand smoke in their homes and cars in socioeconomically contrasting communities: *A qualitative study. Nicotine & Tobacco Research, 16*(11), 1429-1435. doi:10.1093/ntr/ntu096.
2. Wilson, S. I., Ritchie, D., Amos, A., Shaw, A., O’Donnell, R., Mills, L. M., … Turner, S. W. (2013). “I’m not doing this for me”: mother’s accounts of creating smoke-free homes. *Health Education Research, 28*(1), 165-178. doi:10.1093/her/cys082.
3. Phillips, R., Amos, A., Ritchie, D., Cunningham-Burley, S. (2007). Smoking in the home after the smoke-free legislation in Scotland: qualitative study. *BMJ, 335*(7619): 553. doi:10.1136/bmj.39301.497593.55.
4. Rowa-Dewar, N., Lumsdaine, C., & Amos, A. (2015). Protecting children from smoke exposure in disadvantaged homes. Nicotine & Tobacco Research, 17(4), 496-501. doi:10.1093/ntr/ntu217.
5. Amos, A., Morrison, R., Lockhart, D., Ritchie, D., Turner, S., Watson, J., & Semple, S. (In preparation). Using air quality feedback to reduce secondhand smoke levels in homes in an area of deprivation: a community-based approach.

Appendix A

Table 1. Qualitative Data of Studies on SHSe

| Reference | Objective | Sample | Awareness/knowledge of SHS | Strategies (enablers) SHSe | Barriers to SHSe | Conclusions/ Additional info |
| --- | --- | --- | --- | --- | --- | --- |
| Rowa-Dewar et al. 2014. [1]. Scotland | To examine children's accounts of the strategies family members employ to protect them from SHS and to examine how these may be constrained or facilitated in communities with contrasting smoking prevalence rates. | 38 children aged 10-15 years, who lived in 2 Scottish communities of contrasting socioeconomic status and had a close family member who smoked. | Participants expressed strong negative views about smoking in cars and the perceived ineffectiveness of dispersal measures in this context. | Parents were reported to employ spatial and dispersal measures to reduce children's SHS exposure in homes and cars. Smoking was restricted to certain rooms and when those considered more vulnerable were absent. | Less distance between smokers and children, and more smoking in the home were reported in the disadvantaged community, reflecting less space within homes and greater parental smoking. | Although there was general awareness that SHS exposure was potentially harmful, SHS in the home was considered "safe" by some participants if certain conditions were met, particularly by those from the disadvantaged area. |
| Wilson et al. 2013. [2]. Scotland. | To explore mothers’ narratives of changing home smoking behaviours after participating in an intervention (Reducing Families’ Exposure to Smoking in the Home [REFRESH]) aimed at reducing families’ exposure to secondhand smoke (SHS) in homes in Scotland. | 17 mothers with non-smoke-free home at baseline. | Aware that  SHS is harmful to child but had to reassess smoking behaviour (smoking at window) after receiving air quality feedback. | Smoking at kitchen window. Throw away ashtray so that there would be nowhere to extinguish cigarette in house. Child could play outside while mother was smoking. Only smoke indoors when child is not there. Only smoking in kitchen and not living room. Smoking less. Move ashtray into the kitchen. Using social networks as a channel for change (share information with family). | Being lazy. Weather, having to leave children alone (lived at top of 3-story block of flats), increased moodiness and weight gain. Weather, childcare responsibilities. Low level of smoking. Lack of support from partner. Need to see real evidence of the harmful effects of SHS. | Mothers can be supported to make changes which they value and which can reduce SHS exposure in their home. Some mothers living in different domestic circumstances were able to make changes to their smoking that reduced SHS levels in their home. They value their attempts to create a smoke-free home. Changes were incremental which reflected the nature and extent of the barriers that they had to overcome. Importance of attitudes and behaviour of other family members in enabling or blocking change was highlighted. One main motivation for mothers to change their smoking behaviour was their expressed desire to protect their children’s health. Strong moral discourse ran throughout mothers narratives (so should take an assets approach). |
| Phillips et al. 2007 [3]. Scotland. | To explore the accounts of smokers and non-smokers (who live with smokers) of smoking in their homes and cars after the Scottish smoke-free legislation; to examine the reported impact of the legislation on smoking in the home; and to consider the implication for future initiatives aimed at reducing children’s exposure to second-hand smoke in the home. | Fifty adults (aged 18-75) smokers and non-smokers who lived with smokers. | Passive smoking was a well-recognised term.  Respondents had varied understandings of the risks of second-hand smoke, with a few rejecting evidence of such risks. Children, however, were perceived as vulnerable. | Enablers included: The increasing level of restrictions and the reported modification of partial or no restrictions in some circumstances. Increasing awareness of the risks of SHS, particularly in relation to children. Concerns about children and grandchildren not becoming smokers. Desire to be seen as behaving in morally and socially acceptable ways.  Other attempts, both aesthetic and health related, to moderate or remove the perceived negative aspects of smoke in the home. Social norms about the unacceptability of smoking in the home among family and friends, including pressure from children.  Most reported that they restricted smoking in their homes, which were influenced by Spatial, relational, health, and aesthetic factors. Strategies were also used to militate against second-hand smoke, such as opening windows. | Barriers included: Limited understanding of and resistance to messages about the health risks of SHS. Beliefs about the effectiveness of ways of removing or managing SHS in the home. The need to smoke and smoker identity. The home perceived as a private space, protected from public controls and sanctions. Social norms among family and friends about the acceptability of smoking in the home (e.g., concerns of being regarded as hypocritical).  Several spoke about increased feelings of stigma when smoking in public and therefore the increased importance of being able to smoke in private. | The findings indicate that smoking restrictions in the home are shaped by a range of sociocultural influences and other factors that create enablers, and barriers for future public health initiatives on this issue. The author’s suggest that initiatives to reduce second-hand smoke in the  home could include media campaigns and tailored  advice and support for individuals from health and  other professionals on how to develop more effective  smoke-free strategies in the home |
| Rowa-Dewar et al. (2015) [4]. Scotland. | To explore the particular challenges mothers who smoke face when attempting to protect their children from SHS exposure in disadvantaged homes. | Twenty two disadvantaged mothers of children aged 1-3 years. | Mothers reported attempting to protect their children from both SHS and becoming smokers, motivated by the perceived future health and financial burdens these entail.  Strategies to protect children from SHS appeared to be underpinned by an understanding that smoke can be contained and reduced within the home to some extent. | Examples of circumstances which enable reducing SHS in the home include: access to a garden, the presence of a partner, to be and to be seen to be a good mother, and children’s increasing awareness of smoking. | Challenging and changing domestic living circumstances and relationships and the increasing mobility of children in their first few years are key barriers to creating smoke-free homes for disadvantaged mothers. | Smoke-free home interventions should not be one-off but should provide sustained support through key events.  Rather than guilt-inducing SHS communication (stigmatising), which may negatively impact on mother’s future seeking of cessation support, support should build on their motivation to be a good parent. |
| Amos et al. [5]. Scotland | To test the feasibility of delivering the modified version of REFRESH to smoking parents whose children were enrolled at a child and family centre, and to evaluate the experience of participating families and staff. | Five participants of the collective eight took part in the qualitative interviews. | All reported that they were aware of the risks of SHS prior to the project, expressed a desire to protect child health and had already taken steps to limit SHS exposure in the home. | Receiving air quality measurement feedback motivated behaviour change as the feedback created feeling of shock and disbelief.  Facilitators included: help with childcare enabling them to smoke outside, self-imposed restrictions and rules such as removing ashtrays, and external motivating factors such as wanting to make their home smokefree to increase chances of securing childcare rights. | Challenges included difficulties involved in smoking outdoors while watching young children, concerns about making friends smoke outside and maintaining behavioural changes in stressful life circumstances. | Conclusions relate to quantitative section of the study. |
