# Supplementary material for: Development of a Smoke-Free Homes Intervention for Parents: An Intervention Mapping Approach
Source: Health Psychol Bull. Author manuscript; Available in PMC 2020 Apr 24. (PMC7182446; doi:10.5334/hpb.20)
Supplement: Supplementary file 7 [file EMS86099-supplement-Supplementary_file_7_.docx]

**THE AFRESH PROGRAMME**

**May 2016**

Contents

[Summary 4](#_Toc449540557)

[The AFRESH programme flowchart 5](#_Toc449540558)

[Module 1: Parent knowledge, attitudes and beliefs regarding second- hand smoke (SHS) in the home 7](#_Toc449540559)

[a. Establishing context 7](#_Toc449540560)

[b. SHS and child health 8](#_Toc449540561)

[Key Discussion Area 1: The effects of second-hand smoke 8](#_Toc449540562)

[Key Discussion Area 2: How the effects of second-hand smoke apply to your own child/children 9](#_Toc449540563)

[c. Ineffective strategies for eliminating SHS in the home 9](#_Toc449540564)

[d. Barriers and solutions to creating and maintaining a smoke-free home 9](#_Toc449540565)

[e. Summary 10](#_Toc449540566)

[Module 2: Delivering personalised feedback on air quality in the home 11](#_Toc449540567)

[a. Deciding whether to have air quality measured in the home 11](#_Toc449540568)

[b. Reinforcing positive steps already being taken 11](#_Toc449540569)

[c. Establishing context 11](#_Toc449540570)

[d. Installing the air quality monitor 12](#_Toc449540571)

[e. Providing feedback on personalised air quality levels 12](#_Toc449540572)

[Module 3: Barriers, solutions and facilitators to creating a smoke-free home 14](#_Toc449540573)

[a. Facilitators to creating a smoke-free home 14](#_Toc449540574)

[b. Barriers to creating a smoke-free home 14](#_Toc449540575)

[Module 4: Planning for creating a smoke-free home 15](#_Toc449540576)

[a. Developing a personal smoke-free home action plan 15](#_Toc449540577)

[b. Difficult situations and ‘if-then’ plans 15](#_Toc449540578)

[c. Getting support 16](#_Toc449540579)

[d. Personal action plan 16](#_Toc449540580)

[e. Mini-goals 16](#_Toc449540581)

[f. Reviewing the action plan 16](#_Toc449540582)

[g. Praising any success 16](#_Toc449540583)

[h. Things you could do if the mother has had a setback: 16](#_Toc449540584)

[i. Building habits 17](#_Toc449540585)

[Module 5: Equipping mothers to help persuade other household members to create a smoke-free home (optional module) 18](#_Toc449540586)

[a. Why involve family members in creating a smoke-free home? 18](#_Toc449540587)

[b. How to raise the issue of creating a smoke-free home with other household members 18](#_Toc449540588)

[c. Skills for dealing with family barriers to creating a smoke-free home 19](#_Toc449540589)

[d. Planning for a smoke-free home together 19](#_Toc449540590)

[e. Asking visitors for their support 19](#_Toc449540591)

[Module 6: Quitting smoking (optional module) 20](#_Toc449540592)

[a. Why quit? 20](#_Toc449540593)

[b. What tools can help with quitting? 20](#_Toc449540594)

[c. What else can help? 20](#_Toc449540595)

AFRESH Fact Sheet 1: What you need to know about second-hand smoke………………………………………21

AFRESH Fact Sheet 2: Key facts on second-hand smoke and effects on childrens’ health..………………22

AFRESH Handout 1: Parent barriers, facilitators and solutions for creating a smoke-free home………23

AFRESH Handout 2: Example of personalised feedback from a home where smoking takes place…..26

AFRESH Handout 3: Your guide to the air quality intervention…………………………………………………………29

AFRESH Handout 4: Personal smoke-free home action plan…………………………………………………………….31

AFRESH Handout 5: Difficult situations and ‘if-then’ plans……………………………………………………………….32

AFRSH Handout 6: Encouraging other household members to assist with creating a smoke-free home.....................................................................................................................................................33

AFRESH Handout 7: Quitting smoking………………………………………………………………………………………………35

# Summary

The AFRESH programme, which consists of 6 modules, has been developed to assist parents change their smoking behaviour and create a smoke-free home. The programme is designed to be delivered by care professionals, using a combination of face to face discussion techniques, alongside baseline and follow up measures of second hand smoke levels in the home, as illustrated on pages 4-5.

# The programme flowchart

**Materials required:**

- AFRESH Fact Sheets 1 and 2
- AFRESH Handouts 1 and 2

**Contact 1 - Day 1 (takes 20 mins)**

**Module 1: KBA (Knowledge, beliefs and attitudes)**

- Establish context
- SHS and Child Health
- The nature of SHS
- Ineffective strategies
- Barriers and solutions
- Summary
- Invitation to take part in the study

**Materials required:**

- Study information sheet
- Consent form
- Dylos machine and extension
- AFRESH Handout 3

**Contact 2 – Day 3 (takes 10 minutes)**

- Confirm the parent has read the information sheet
- Answer any questions
- Obtain consent to take part
- Provide parent with Dylos machine to take home and install for 5 days

**Contact 3 – Approx. day 9 (takes 60 minutes, can also be split into 2 separate sessions)**

**Module 2 - Feedback**

**Materials required:**

- AFRESH Handout 3
- Personalised feedback data

- Mother returns Dylos machine

(OR staff member collects it from mother’s home)

- Staff member downloads feedback

(OR downloads within the next 3 days)

- Staff member provides indoor air quality feedback (OR visits mother in home to provide feedback)

**Module 3: Solutions**

**Materials required:**

- AFRESH Handout 1

- Facilitators, barriers and solutions

**Module 4: Planning**

**Materials required:**

- AFRESH Handouts 4 and 5
- AFRESH Handout 1 (optional)
- ‘If-then’ plans
- Getting support
- Personal plan
- Why involve others
- How to raise the issue
- Skills for dealing with barriers

**Materials required:**

- AFRESH Handouts 1 and 6

**Module 5: Dealing with others (optional)**

**Contact 4 – Between two-four weeks after Contact 3 (5 minutes)**

**Materials required:**

- Preparing Dylos for re-installation (*information provided separately*)
- Dylos machine and extension
- Provide parent with Dylos machine to take home and install for 5 days

**Contact 5 – Approx. 6 days later (20 minutes, or can be split into two sessions)**

**Refer back to Module 2 – Feedback and Review Progress**

**Materials required:**

- Dylos software (*provided separately)*
- Download instructions (*provided separately)*
- Personalised feedback data
- Personalised comparison data
- AFRESH Handouts 4 and 5
- Mother returns Dylos machine

(OR staff member collects it from mother’s home)

- Staff member downloads feedback

(OR downloads within the next 3 days)

- Staff member provides indoor air quality feedback (OR visits mother in home to provide feedback)
- Staff member and mother compare feedback with that from Contact 2 and review action plan

**Materials required:**

- AFRESH Handout 7
- This module may be delivered at any stage of the programme should a mother decide that she wants to quit smoking completely.

**Module 6 – ‘Quitting smoking’ (optional)**

# Module 1: Parent knowledge, attitudes and beliefs regarding second- hand smoke (SHS) in the home

***Materials required to deliver Module 1:***

- ***AFRESH Fact Sheets 1 and 2:***
  - ***What you need to know about second-hand smoke***
  - ***Second-hand smoke and effects on children’s health***
- ***AFRESH Handout 1: Parent barriers, facilitators and solutions for creating a smoke-free home***
- ***AFRESH Handout 2: Colour print out of example of personalised feedback from a home where smoking takes place***

## a. Establishing context

The care professional begins by establishing with the parent:

- Who currently smokes in the house and where
- How many cigarettes are smoked per day by each smoker in the home
- How many children live in the house, and their ages
- Any other household members
- Current rules for smokers who visit the house – where are they allowed to smoke?
- Are there any times when the existing smoke-free rules don’t apply (i.e. when children not present, when children are asleep, when the weather is bad etc.)
- Has the mother has ever considered making the home smoke-free before, and why (not)?

During the course of these discussions, or as a result of previous interactions with the mother, the care professional may have concerns about potential domestic abuse that could arise as a result of the mother initiating steps to make her home smoke-free.

In such instances the care professional should make a case-by-case judgement about the mothers’ participation in the study. For example, any of the following scenarios could be applied:

a. The care professional may consider that the domestic situation is too volatile for the mother to participate in the study

b. The care professional and the mother may agree that the mother will focus on changing her smoking behaviour in the home, without asking others in the household to do the same (so omit delivering Module 5). In this scenario, the care professional should emphasise that reducing SHS levels in the home will be beneficial to her child/children. In addition, the care professional and the mother may agree *not* to place the air quality monitor in the home to record personalised SHS levels (so omit delivering Module 2).

If b, the care professional should use their own judgement to tailor the discussions that follow so that they are more of a ‘light touch’ approach. For example, spend less time focusing on the health impacts of second hand smoke in the home, which may cause additional feelings of guilt for the mother given she may feel unable to control creating a smoke-free home at this time. Focus more on the steps that she can practically take to reduce exposure levels.

## b. SHS and child health

The health/social care professional uses a **tailored** approach to first identify what the mother already knows about SHS in the home; reinforces/confirms existing correct knowledge; addresses any misconceptions and provides new information where knowledge is missing. The care professional **discusses** health harms of SHS exposure with the parent and how this may apply to their children (to personalise risk), listening to the mother to establish whether she comprehends the information, and addressing any misconceptions.

- Discussion should cover key areas 1 and 2 below
- Remember throughout to *reinforce* existing knowledge, *address* any misconceptions, and *provide* new information where knowledge is missing
- Refer to the **AFRESH factsheets 1 and 2** if you need to, to help deliver this information

### Key Discussion Area 1: The effects of second-hand smoke

- 1. Ask the mother what she knows about second hand smoke and its effects in general
  2. Explain why second-hand smoke is dangerous
  3. Ask the mother how common she thinks it is for other parents keep a smoke-free home
  4. Explain that children whose parents smoke around them are three times more likely to take up smoking as a teenager
  5. Explain that children have smaller lungs, and breathe faster, and so are particularly susceptible to the effects of SHS (this is similar for pets too).
  6. Outline the effects of SHS exposure on child health

Using elaboration techniques may make it more likely that mothers remember new information on second-hand smoke. The care professional should try providing some of the information from Factsheet 1, and ask the parent to **elaborate** with any additional information she knows of. For example, they might say:

- Did you know there is no safe level of SHS exposure in the home?

And then ask parents to elaborate with other information they (are more likely to) know, such as:

- SHS travels through different rooms in the house
- After smoking a cigarette, SHS can linger in the house for 5 hours

### Key Discussion Area 2: How the effects of second-hand smoke apply to your own child/children

- 1. Ask if their child/children have/have ever had had any of the health conditions: coughs, ear infections, chest infections (for example bronchitis, bronchiolitis, pneumonia), wheezing, asthma
  2. If yes: explain that these conditions can be caused by exposure to second-hand smoke

If no: explain there is a higher likelihood that their children will develop such conditions compared to children who live in a smoke-free home

## c. Ineffective strategies for eliminating SHS in the home

The Scottish Government’s ‘Take it Right Outside’ video demonstrates how it’s never safe to smoke indoors, and could be used as a visual aid during this section:

<http://www.rightoutside.org/>

The care professional **discusses** ineffective strategies with the parent, listening to ensure the mother understands the information, and addressing any misconceptions she may have. Ineffective strategies include:

- Opening a window/smoking out of a window
- Smoking in the kitchen or other designated room
- Smoking inside, by the front/back door *(page 9 of the REFRESH How to Guide could be used to demonstrate this point)*
- Using a fan
- Burning candles
- Smoking when children aren’t present

## d. Barriers and solutions to creating and maintaining a smoke-free home

Use the AFRESH handout 1 to aid discussion in this section: *‘Parent barriers, facilitators and solutions for creating a smoke-free home’*

The care professional **discusses** with the parent some of the general barriers that other parents have found in trying to create a smoke-free home. This should include barriers relating to the parent themselves, barriers relating to other household members, and barriers relating to the housing environment if applicable.

To illustrate the impact that barriers have on creating a smoke-free home, the care professional should show the parent Handout 2, the personalised feedback graph from a home similar to theirs, where one or more parent is smoking in the home.

This is partly for illustration purposes, and also to show them what the feedback graph looks like if they choose to have measurements made in their own home. The care professional should also comment that by learning from previous difficulties in making the home smoke-free, people are more successful in their next attempt.

The care professional then discusses facilitators with parents, encouraging them to come up with effective strategies for exploring solutions.

## e. Summary

The care professional takes time to summarise (a) what parents already know about SHS exposure in the home and (b) what steps are already being taken in the home to reduce SHS levels. The professional checks that their summary matches with the parents’ perspective of current knowledge/steps being taken and any discrepancies are discussed and resolved.

# Module 2: Delivering personalised feedback on air quality in the home

***Materials required to deliver Module 2:***

- ***AFRESH Handout 3: Your guide to the air quality intervention***
- ***Colour print outs of personalised feedback data***
- ***Colour print out of personalised comparison data, for follow up sessions only***

## a. Deciding whether to have air quality measured in the home

*‘AFRESH Your guide to the air quality intervention’* includes more information on the Dylos air quality monitor, and the procedures involved in having air quality measured. This leaflet should be used to aid discussion in this section, **UNLESS** the intervention is being delivered as part of a research project, in which case refer to the research information sheet that will have been designed for the project, and follow informed consent procedures.

The care professional should ask mothers if they would like to see what the air quality is like in their own home. Be clear that this is the mother’s choice. They should in no way feel pressured to have an air quality monitor placed in their home. Using the AFRESH intervention guide, the care professional should:

- Explain what will happen during the intervention
- Explain what the air quality monitor does
- Show the mother what an air quality monitor looks like, either using a picture, or using a real-life example.
- Answer any questions that mothers may have using the AFRESH intervention guide.

If the mother decides not to have the air quality monitor installed in her home, reassure her that that’s ok. If the mother agrees to have her air quality monitored, let her know that you support her decision and continue with the next section of this module.

## b. Reinforcing positive steps already being taken

The health and social professional first uses **self-affirmation** techniques to reinforce and encourage parents to elaborate on the positive steps they have already taken to reduce SHS levels in the home (i.e. restricting smoking to particular rooms etc.). This should increase parents’ self-image and lower resistance to the personalised feedback if SHS levels are higher than the parent is expecting.

## c. Establishing context

Before the machine is installed in the home, the care professional should establish the following:

- who currently smokes in the house and where
- approximately how many cigarettes are smoked per day by each smoker in the home
- how many children currently live in the home, and their ages

The care professional then explains that, by building on what the parent already knows, and what they are already doing, they will be able to better protect their child/children from SHS in the home.

## d. Installing the air quality monitor

The care professional should give the mother the air quality monitor to install at home, explaining that:

- The air quality monitor should be placed in the living room, out of the reach of children (for example, on a shelf, on top of the television, or on a window sill)
- To install, simply plug in and switch on.
- The monitor should not be switched off overnight. It is safe to leave it running continuously, and this is required for the purposes of the intervention.
- The monitor uses minimal electricity – approximately 10p per day.

The care professional should then agree with the mother a convenient day for her to return the air quality monitor. The duration can be tailored to suit the needs of the mother taking part. The device will log the latest 6 days of measurements so generally return should take place within a week. Ideally measurements should span 5 days with some data from weekends included if possible.

## e. Providing feedback on personalised air quality levels

Once Dylos data has been downloaded (refer to ‘*Using the AFRESH software*’, provided separately, for step-by step instructions on downloading), the care professional uses **individualisation** and **personalised risk** techniques in providing personalised air quality feedback, giving mothers opportunities to have questions answered according to their individual feedback and progress.

Have a colour print out of the personalised air quality feedback to hand!

At this point, the care professional shares the graphs of personalised Dylos feedback with the mother. This includes discussion of:

- average particle (PM2.5) levels
- Lowest and highest PM2.5 level
- the percentage of time when the PM2.5 levels exceed World Health Organisation guidance
- the percentage of time when the household PM2.5 levels were higher than those measured in a typical bar before smoke-free legislation came into force in Scotland
- how the average level compares to other homes in Scotland where similar measurements have been carried out.

Visual information is then given on whether their current PM2.5 level is satisfactory in terms of risks to child health, and, if applicable, what more they need to do to reach satisfactory levels.

*At this point,* ***at further follow up sessions only,*** *any smoking behaviour change should also be discussed since PM2.5 was last measured, with a view to ascertaining whether changes have arisen as a result of the air quality monitoring, or whether there are other factors involved. If other factors are involved, the professional should find out more about them and their impact. Use the personalised comparison feedback graph as a starting point for discussion.*

The care professional then discusses air quality findings with the parent, to include:

- Parent reactions to feedback – how do they feel about the PM2.5 levels recorded?
- To what extent did they think their child was at risk from SHS exposure in the home, and has their view changed now having seen the feedback?
- What changes would they like to make to their smoking behaviour in the home? Are these changes realistic?

By this point, the care professional should be beginning to identify with the parent a realistic, but potentially challenging goal that they can set to achieve a smoke-free home.

# Module 3: Barriers, solutions and facilitators to creating a smoke-free home

***Materials required to deliver Module 3:***

- ***AFRESH handout 1: Parent barriers, facilitators and solutions for creating a smoke-free home OR*** [*www.rightoutside.org/yoursmoke-freetips*](http://www.rightoutside.org/yoursmokefreetips)

## a. Facilitators to creating a smoke-free home

The care professional should discuss with the parent the reasons why they want to change their smoking behaviour to establish a smoke-free home.

Encourage the parent to identify these ‘facilitators’ themselves, by asking them to complete the following sentence: ‘I would like to have a smoke-free home because…’

The care professional should then confirm the parent’s beliefs, and add new suggestions for facilitators. Encourage parents to elaborate on additional suggestions that apply to them as well. At this point, the care professional should also correct any existing beliefs about facilitators that the parent has that are incorrect, if applicable.

## b. Barriers to creating a smoke-free home

The care professional then encourages the parent to identify barriers to creating their smoke-free home. This can be done in conjunction with planning coping responses, by involving the parent in making a personalised list identifying high-risk situations/barriers and a plan for coping with them. The care professional should then encourage the mother to identify practical steps that need to be taken in order for her to reach the goal of creating a smoke-free home.

At this point, the care professional can share *Handout 1: Parent barriers, facilitators and solutions for creating a smoke-free home,* so that mothers can see how other parents have overcome challenges in creating a smoke-free home.

# Module 4: Planning for creating a smoke-free home

***Materials required to deliver Module 4:***

- ***AFRESH Handout 4: Personal smoke-free home action plan***
- ***AFRESH Handout 5: Difficult situations and ‘if-then’ plan***
- ***AFRESH Handout 1: Parent barriers, facilitators and solutions for creating a smoke-free home (optional)***

## Developing a personal smoke-free home action plan

The care professional opens the discussion by explaining that behaviour change is helped by a detailed plan of how mothers are going to change their behaviour:

- Creating a smoke-free home will have a positive impact on how you feel about yourself, and improve the health of your child/children. But creating a smoke-free home can be easier said than done. Even if you are strongly motivated, breaking old habits can be a challenge.
- Goals are easier to reach if they’re specific (“I’ll ask my friend to watch my child while I nip outside for a cigarette”, rather than “I’ll smoke outside”)
- It’s not enough to have a goal of creating a smoke-free home. You also need a plan which outlines practical ways to reach your goal.

At this point, the care professional should work through Section 1 of *Handout 4: Personal smoke-free home action plan,* with the mother, confirming her general goal to create a smoke-free home, and identifying associated specific goals.

## b. Difficult situations and ‘if-then’ plans

*Handout 5: Difficult situations and ‘if-then’ plans’* should be used at this point to encourage the parent to plan ways of overcoming her own challenges in establishing a smoke-free home. associated specific goals.

The care professional should encourage the mother to make a list of ‘if-then’ plans using the template provided in Handout 5, i.e. ‘*if situation Y arises, then I will perform behaviour X’*. The situation itself then becomes a trigger for performing the desired behaviour, for example: *“If it is a weekday morning, then I will go outside and have a cigarette before my partner goes to work.”*

The care professional may also want to refer back *to Handout 1: Parent barriers, facilitators and solutions for creating a smoke-free home* to aid discussions with the mother at this stage.

## c. Getting support

Planning where the mother can get support for creating a smoke-free home is also important. Ask the mother to think of people who are likely to be supportive and encourage them in changing their smoking behaviour. Together think of ways that the mother can get the most benefit from these people, for example, spending more time with them, having a specific person to phone when they need encouragement. Help the mother to identify any useful sources of support and encourage them to use them.

## d. Personal action plan

At the end of the meeting, the mother completes her Personal Action Plan and both the mother and the care professional sign it. This is effectively a behaviour change ‘contract’ – making contracts has been shown to help people stick to their action plans and achieve their goals.

## e. Mini-goals

Once the mother has filled in their Personal Action Plan, the care professional should help them to work out what their mini-goals could be. It is important to remember that the action plan should be reviewed regularly and revised in the light of experience. Mothers should be told that they won’t necessarily stick to their first plan, but their experience of trying to do this will give very helpful information that can be used to revise the plan to make it work better for them.

## f. Reviewing the action plan

Reviewing the action plan involves finding answers to the following questions:

- What was tried?
- With what effects (and did the mother achieve any success)?
- What benefits were there?
- What difficulties were there?
- How did the mother manage any difficulties?
- Were there any problems recording behaviour? How could these be solved?
- How confident is the mother that they can achieve their goal?
- Is the mother getting enough support?
- Does the action plan need changing? (The mini goal (s))

## g. Praising any success

Make sure you praise something that the mother has done, however small, to boost their motivation and level of self-confidence. This could include any progress made towards creating a smoke-free home, attending the meeting, recording behaviour or learning more about what they find difficult. Praising the mother may also improve your relationship with them and make communicating easier. Remind the mother that successfully creating a smoke-free home may be a long process in some households, and emphasise the need to build on each small success, learning from any setbacks.

## h. Things you could do if the mother has had a setback:

- Review the goal – look at the goal that was set. Was it realistic? If not, try setting another goal that the mother will find easier to achieve. Agree that you set the goal too high before.

- Barriers and facilitators – were there barriers that the mother hadn’t thought about? If so, the barrier, and possible solutions, need to be discussed
- Self-monitoring – is the mother still recording their behaviour? If they’ve stopped, it’s easy for them not to realise why the setback has happened. Talk about what is making it difficult for them to record their behaviour, and together come up with ideas that could help.
- Difficult situations – review or fill in the ‘if-then’ table.
- Support – check that the mother is receiving enough support from friends and family. Talk about ways of getting more support, and provide information on the types of support available from the community.

## i. Building habits

Once a mother has successfully changed her smoking behaviour, the next stage is to maintain this behaviour and continue it into the foreseeable future. Having a smoke-free home needs to become part of their lifestyle, an automatic habit that doesn’t require effort or thinking.

#### Support

Check that the mother is continuing to use the support they can get from friends and family.

#### Repetition

Simply by repeatedly taking their smoking outside, it is more likely to become a habit. The more frequently a mother performs the behaviour, the less time they spend deciding whether or not to do it and thinking about why they are doing it. As the mother repeats the behaviour many times, it takes less conscious effort and attention, and then becomes a habit.

#### Reviewing progress, benefits and outcomes

Encourage the client to look back at what they have already achieved. Reinforce the benefits that smoking outside has had or will have. Remind the mother of the skills they have acquired. The fact that they have smoked outside before should increase their confidence that they can do it again. The skills they have acquired may also give them the confidence to think about changing other health behaviours in the longer term.

# Module 5: Equipping mothers to help persuade other household members to create a smoke-free home (optional module)

***Materials required to deliver Module 5:***

- ***AFRESH Handout 6: Encouraging other household member to assist with creating a smoke-free home***
- ***AFRESH Handout 1: Parent barriers, facilitators and solutions for creating a smoke-free home***

## a. Why involve family members in creating a smoke-free home?

Handout 6 contains guidance for mothers on helping to persuade other household members to assist with creating their smoke-free home. The contents of Handout 6 should form the basis of the discussion between the care professional and the mother. In addition, the mother can refer to Handouts 1, 2, and 4-5 and 7 in her own discussions with other household members.

The care professional should emphasise that children will only fully benefit if others in the household smoke outside too. Reiterate that mothers are not asking family members to quit smoking altogether, but to smoke outside.

## b. How to raise the issue of creating a smoke-free home with other household members

The care professional should discuss with the mother her responsibility to protect her child/children from the SHS of others, and ways of initiating a discussion with other household members about their role in assisting with this. There are many ways of doing this, including:

- Sharing and discussing facts that are most relevant to household members from Module 1
- Sharing personalised feedback graphs regarding actual PM2.5 levels in the home
- Explaining an advantage of other household members trying to smoke outside and asking them for any additional advantages they can think of.
- Asking family members what they think/feel about their smoking in the home
- Encouraging other household members to reflect on the impact of their smoking behaviour on the well being of others in the household.

The parent should be encouraged to select the strategies that are most appropriate for them, including sharing the personalised feedback graphs regarding measured PM2.5 levels in the home.

## c. Skills for dealing with family barriers to creating a smoke-free home

This section outlines ways in which the care professional can equip the mother with skills for dealing with family barriers to having a smoke-free home.

*Handout 1: Parent barriers, facilitators and solutions for creating a smoke-free home* can be used here, to help the care professional and mother explore together barriers with family members, and solutions to overcome them.

During this section, it is key to explore together potential barriers with family members. The care professional should ask the mother to list any potential barriers with family members that they can think of, and then identify solutions to overcome them.

The mother should also be encouraged to rehearse methods of dealing with barriers with family members, partly to increase her own confidence and partly to practice coping responses associated with raising problems with family members if discrepancies occur.

The care professional should also discuss with the mother strategies for asking visitors for their support in making the home smoke-free. Similarly, the care professional should ask the mother to list any potential barriers with visitors that they can think of, and then identify together solutions to overcome them.

The mother should then be encouraged to rehearse methods of dealing with barriers with visitors, partly to increase her own confidence and partly to practice coping responses associated with raising problems with family/friends if discrepancies occur.

The care professional should remind the mother that most barriers can be overcome, especially when possible solutions are discussed together. However, if it becomes clear that other household members are unable or unwilling to assist the mother in creating a smoke-free home, suggest that the mother does the best she can to reduce second-hand smoke levels in her home by taking her own smoking outside.

## d. Planning for a smoke-free home together

If other household members are able to assist in creating a smoke-free home, suggest that the mother talk with them about how they will achieve this. They may want to complete their own personal action plan, and/or develop their own ‘if-then’ plans. Emphasise the importance of monitoring progress together, discussing any setbacks as they arise, and praising successes.

## e. Asking visitors for their support

Encourage the mother to think about strategies for asking visitors for their support in making the home smoke-free, just as they have done with family members.

# Module 6: Quitting smoking (optional module)

***Materials required to deliver Module 6:***

- ***AFRESH Handout 7: Quitting smoking***

At any stage of this programme, if a mother decides she wants to quit smoking completely, *Handout 7: Quitting smoking* should be used as a basis for discussing quit options and actions.

## a. Why quit?

The care professional should discuss with the mother her reasons for wanting to quit, and encourage her by pointing out that:

- Stopping smoking is the single most important thing a smoker can do to improve their health
- Stopping smoking immediately reduces the risk of heart disease, cancer, stroke, diabetes, rheumatoid arthritis and dementia
- A 20-a-day smoker paying £7.50 a packet will save over £2700 a year.

## b. What tools can help with quitting?

The care professional should discuss the different tools that are available to help with quitting smoking. Emphasise that different things work for different people. If the mother has tried to stop smoking before using one of the products listed, suggest that she could try something else:

- Nicotine therapies, such as chewing gum, patches, lozenges and inhalers can be provided free by NHS stop smoking services
- GPs may be able to prescribe stop smoking medicines
- Some people have used e-cigarettes to help them quit. We don’t know how effective they are, but some users have found them helpful. If this is the route they choose, explain that:
  - The vapours produced by e-cigarettes contain nicotine, but they are likely to be less harmful to children than second-hand smoke.
  - Parents may want to consider using e-cigarettes out of the sight of children. The hand to mouth action mimics that of smoking, which children may go on to imitate. Children may also find brightly coloured lights and flavoured smells associated with some e-cigarettes attractive.

## c. What else can help?

Be clear that stop smoking services offer the best chance of quitting smoking. Encourage the mother to either:

- Contact Smokeline on **0800 84 84 84** (8am – 10pm 7 days a week) to speak with an advisor
- Visit [www.canstopsmoking.com](http://www.canstopsmoking.com) for help, information or to web chat with an advisor
- Make an appointment with their GP who can refer them to their local stop smoking service
- Visit their local pharmacy to talk to their pharmacist about quitting

**AFRESH: FACT SHEET 1**

**WHAT YOU NEED TO KNOW ABOUT SECOND-HAND SMOKE^[[1]](#footnote-1)^^[[2]](#footnote-2)^^[[3]](#footnote-3)^**

**What is second-hand smoke?**

- Second-hand smoke is all the particles and vapours that go into the air when a tobacco product is burned. That means both smoke coming off the lit end of a cigarette, as well as smoke breathed out after taking a drag.
- More than 85% of tobacco smoke is invisible and has no smell.

**Why is second-hand smoke dangerous?**

- Ninety chemicals in second-hand smoke are thought to cause cancer, while others cause diseases like glue ear, meningitis and cot death. These chemicals can quickly build to dangerous levels in a home or car, even after just one cigarette.
- In a home, second-hand smoke can remain at dangerous levels for more than five hours. Levels can still be high everywhere in the home even if you open the windows or you only smoke in one room.
- There is no safe level of second-hand smoke in the home.

**Why are children particularly vulnerable to second-hand smoke?**

- Children have small, growing lungs, and breathe more deeply and more quickly than adults. Because of this, they can suffer more from the effects of second-hand smoke. For that reason, it’s important to keep children safe from second-hand smoke by never smoking inside the home or car.

**Is smoking in the home something that lots of other parents do?**

- Approximately 90% of children living in Scotland under the age of 16 live in smoke-free households.

**AFRESH FACT SHEET 2**

**KEY FACTS**^[[4]](#footnote-4)^1 **ON SECOND-HAND SMOKE AND EFFECTS ON CHILDRENS’ HEALTH**

**Second-hand smoke exposure has been shown to have a number of harmful effects on children’s health:**

- Children and infants are more vulnerable to tobacco smoke than adults. Children have smaller airways, breathe faster and their immune systems are still developing.
- A woman who smokes one to nine cigarettes a day during pregnancy is more than four times as likely to have a baby die as a cot death than a woman who doesn’t smoke at all during pregnancy
- Tobacco smoke can cause low birth weight in babies which has been associated with coronary heart disease, type 2 adult onset diabetes, and being overweight in adulthood
- Middle ear infection in a child is 50% more likely to occur if one parent smokes
- Children whose parents smoke at home are twice as likely to have asthma symptoms all year round
- Exposure to second-hand smoke is associated with an increased risk of respiratory tract infections such as coughing, wheezing and croup
- Approximately 9,500 children are admitted into hospital in the UK every year because of tobacco smoke
- Children are three times more likely to smoke when they get older if they grow up around smokers.

**AFRESH HANDOUT 1: PARENT BARRIERS, FACILITATORS AND SOLUTIONS FOR CREATING A SMOKE-FREE HOME**

| **POSSIBLE BARRIERS TO CREATING**  **A SMOKE-FREE HOME** | **POSSIBLE SOLUTIONS** |
| --- | --- |
| How do I handle the situation when my partner/visitors smoke? I’m concerned it may upset relationships in the home. | - Remember that you’re protecting both your child/children and others from the e­ffects of second-hand smoke. - Ask friends/partners for their support in helping make the home smoke-free. Most people respond well to requests for help so don’t be afraid to ask for their support. - Remind your partner/family/friends that you are not asking them to stop smoking altogether but to smoke outside if they want to have a smoke. - You could have a no-smoking sticker in the home or by the door, or ask your child to draw a smoke-free home poster, to remind visitors. |
| I live in a high-rise flat and don’t have access to a balcony where I could smoke OR I can’t leave my children alone whilst I go outside to smoke. | The safety of your children is essential. We know it can seem like you don’t have a choice, but there are other ways that might work for you and your kids:   - Do you live with someone who could look after the children while you go outside for a smoke? Or do you have a friend that could come over and mind your child whilst you nip outside for a cigarette? You could always offer to do the same for your friend if they smoke too. - Is there a safe area outside where your child/children could play while you have a cigarette? - Could you wait and have a cigarette when you are outside (e.g. on the way to the shops, during the nursery/school run, or whilst you walk the dog if you have one)? - You could consider using NRT products such as an inhalator or gum while in the flat. If you decide to use NRT, we recommend they take advice from a pharmacist or GP as to the product that would best suit your needs. |
| What do I do about the cravings that I get when I get stressed in the house? | - Most cravings will subside within a few minutes and doing something   different will take your mind o­ff cigarettes.   - Sipping water, chewing gum, or brushing your teeth can help with cravings. - Some parents find it helpful to take up a hobby that uses their hands to help take their mind o­ff the cravings (and help keep their hands off­ the cigarettes). Maybe drawing, or colouring in with your child might help? - Some parents find that phoning a friend or family member takes their mind off the cravings. Try and speak with someone who will be a source of support and encouragement for you. - Maybe you could go for a walk with your child/children, or play a game with them. - You could consider using NRT products such as an inhalator or gum while in the flat. If you decide to use NRT, we recommend they take advice from a pharmacist or GP as to the product that would best suit your needs. |
| I think that the e­ffects of second-hand smoke are exaggerated as my parents smoked when I was a kid and I’m fine. | There is a lot more information available these days on the health effects of second-hand smoke exposure in the home, compared to 20 years ago, when we knew very little about the impacts on children. A lot of the research that has been conducted comes from right here in Scotland!   - Have a look at the facts section of the ‘Right Outside’ website. Here you’ll find more information on harmful effects of second-hand smoke exposure in the home, including some of the key impacts on child health such as ear infections, chest infections and asthma: <http://www.rightoutside.org/facts> - You could also take a look at the ‘Right Outside’ video here, to learn about how second hand smoke lingers in your home after smoking: <https://youtu.be/OJaNZcgcvVc> |
| Can I smoke in one room with the window open/back door open? | Sometimes parents think that smoking in the kitchen with the window open, for example, is enough to protect their children from second hand smoke in the home:   - Smoking in a room with a window open, with an extractor fan on, or by the back door will lessen some of the smoke in the home, but it doesn’t remove the health risks because many of the harmful toxins in second-hand smoke remain in the room for many hours (see the ‘Right Outside’ video here: <https://youtu.be/OJaNZcgcvVc>). - Smoking outside of the home/car is the only way to adequately protect children from the health risks of second-hand smoke. - Perhaps you could put an ashtray and umbrella by the back door. If it’s possible, try to create a small area of the garden or communal area where you can smoke well away from the back door. |
| If I reduce the amount I smoke I might put on weight. | - You don’t have to give up smoking completely to protect your child/children from second hand smoke. People don’t always put on weight when they stop or reduce smoking. - Don’t let the fear of weight gain keep you from making a positive change to a smoke-free home which will beneficial for all of the family. - Tackle one thing at a time, and remember it’s a great achievement to create a smoke-free home. - If you do end up gaining a few pounds during the process, you can lose them later on with the same determination you are using to create a smoke-free home. Regular moderate exercise and avoiding high calorie snacks should help them avoid putting on any extra pounds. |

*Adapted from the REFRESH How to Guide (2012):* [*http://www.ashscotland.org.uk/media/458831/refresh_howtoguidecreate-a-smokefree-homejan12.pdf*](http://www.ashscotland.org.uk/media/458831/refresh_howtoguidecreate-a-smokefree-homejan12.pdf)

**AFRESH HANDOUT 2: EXAMPLE OF PERSONALISED FEEDBACK FROM A HOME WHERE SMOKING TAKES PLACE**


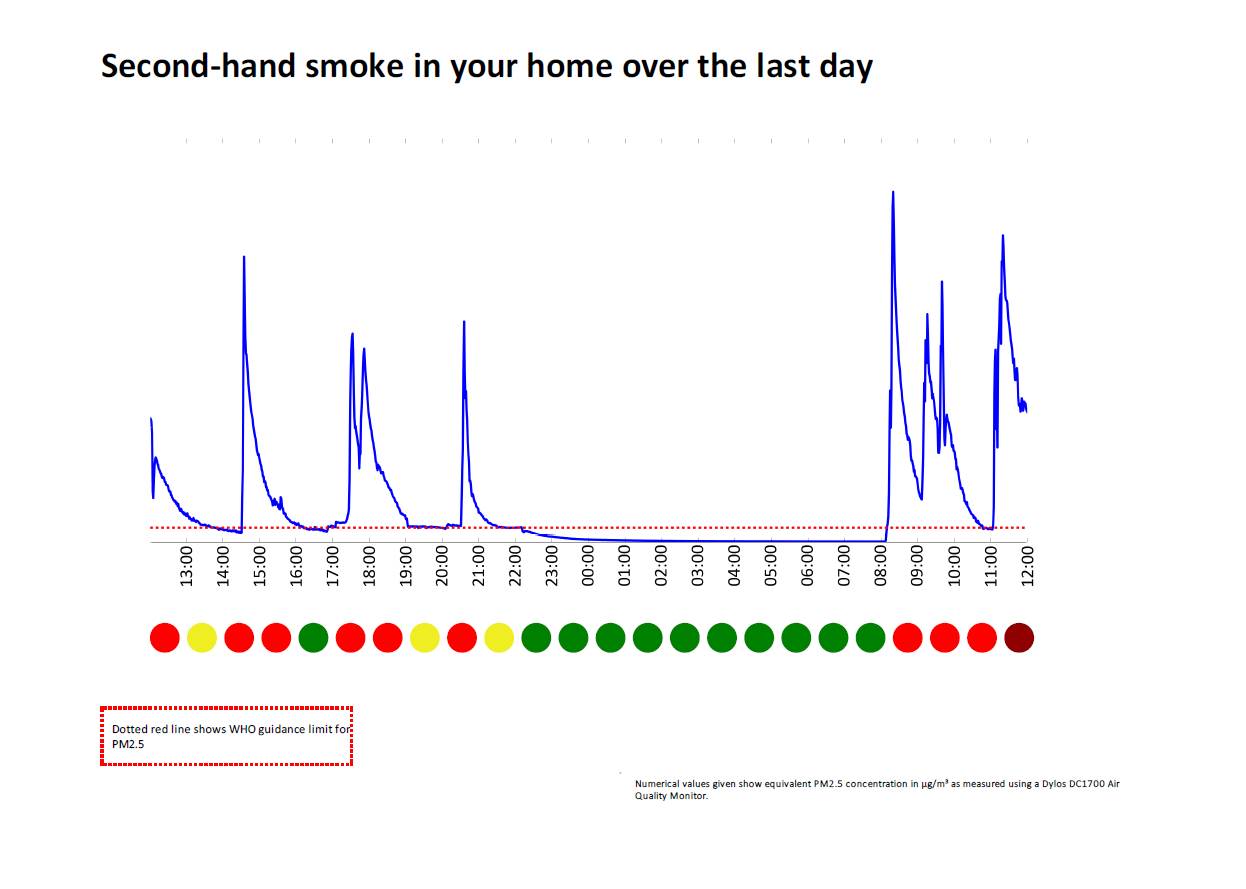


*(Numerical values given show equivalent PM2.5 concentrations in µg/m^3^ as measured using a Dylos DC1700 Air Quality Monitor)*

**AFRESH HANDOUT 2: EXAMPLE OF PERSONALISED FEEDBACK FROM A HOME WHERE SMOKING TAKES PLACE (cont.)**


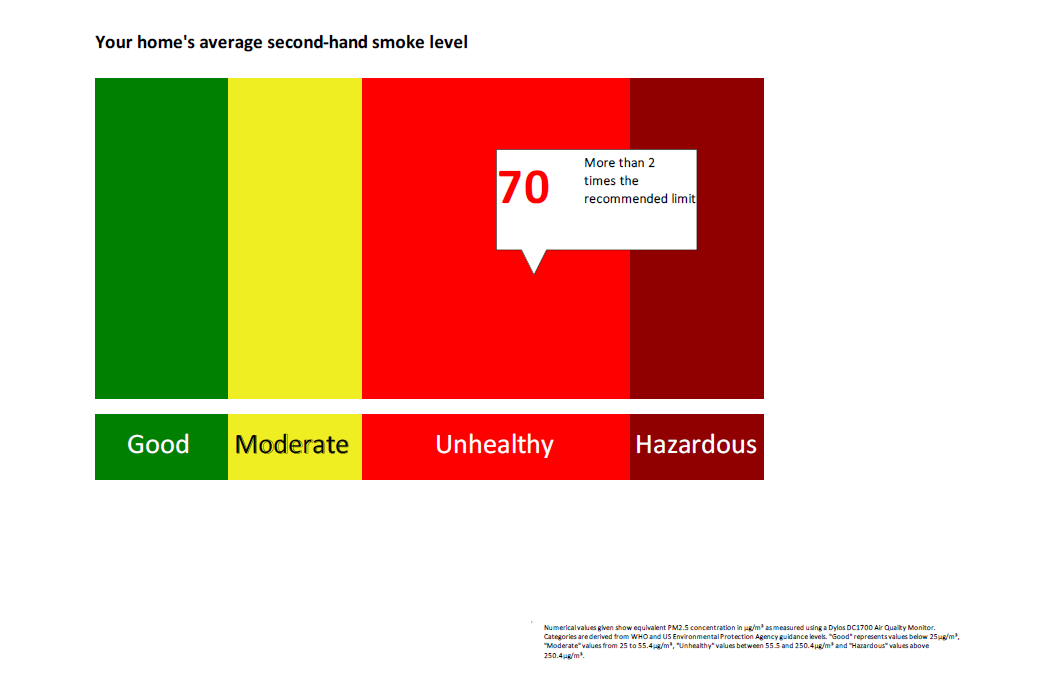


*(Numerical values given show equivalent PM2.5 concentrations in µg/m^3^as measured using a Dylos DC1700 Air Quality Monitor. Categories are derived from WHO and US Environmental Protection Agency guidance levels. ‘Good’ represents values below 25µg/m^3^, ‘Moderate’ represents values from 25 to 55.4µg/m^3^, ‘Unhealthy’ represents levels between 55.5-250.4µg/m^3^ and ‘Hazardous’ represents values above 250.4µg/m^3^)*

**AFRESH HANDOUT 2: EXAMPLE OF PERSONALISED FEEDBACK FROM A HOME WHERE SMOKING TAKES PLACE (cont.)**


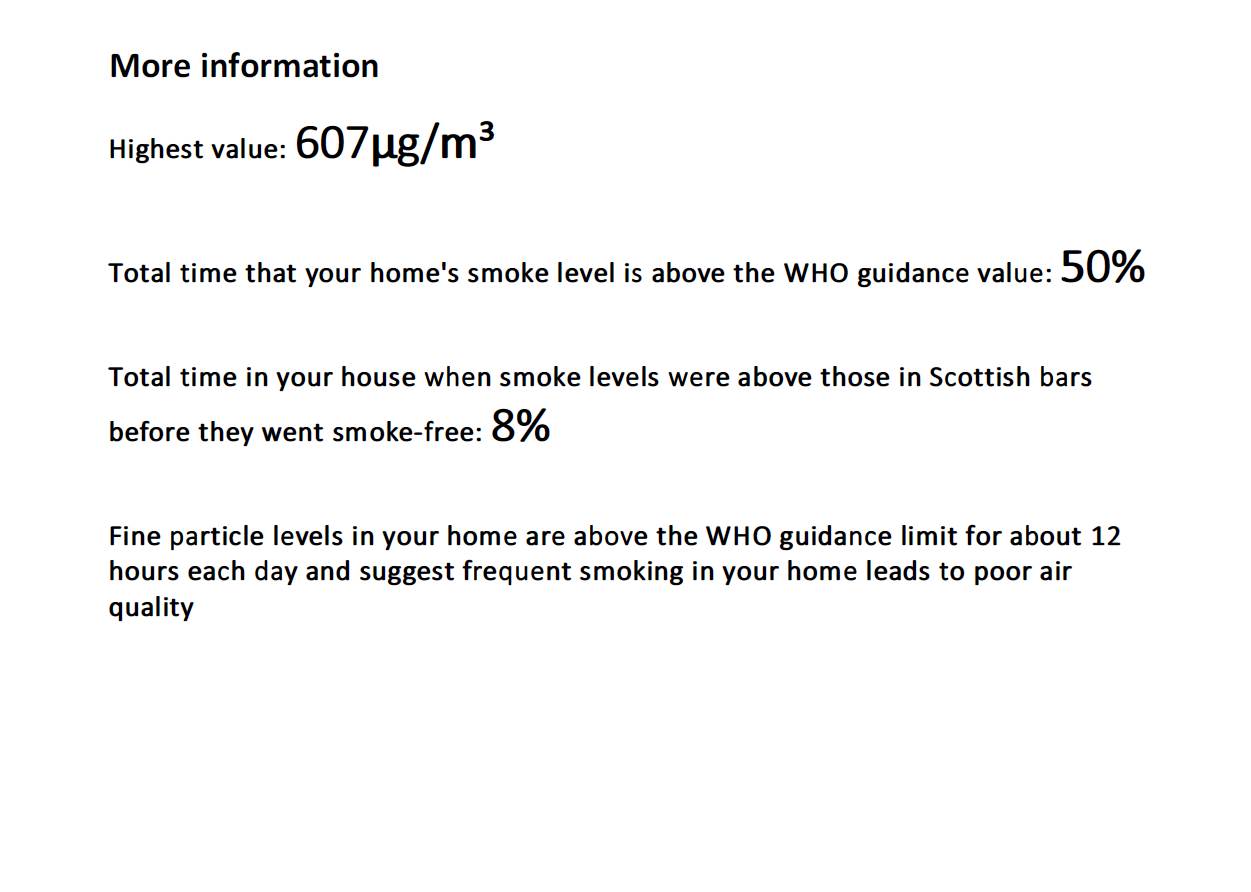


**AFRESH HANDOUT 3: YOUR GUIDE TO THE AIR QUALITY INTERVENTION**

- **What is the air quality intervention?**

Most parents know that second-hand smoke is dangerous and take steps to keep their children safe. But many people don’t realise that smoke can hang around in the air for many hours, even though you can’t see it or smell it.

If you use an air quality monitoring machine, you can count the invisible particles of smoke that are in someone’s home and turn the information into a graph. That lets you see if there are sometimes dangerous levels of smoke in the air.

It also shows you when there aren’t particles in the air. That means that if the air is mostly OK but sometimes has high levels of particles, you can focus on doing things to get the levels down when you need to.

-
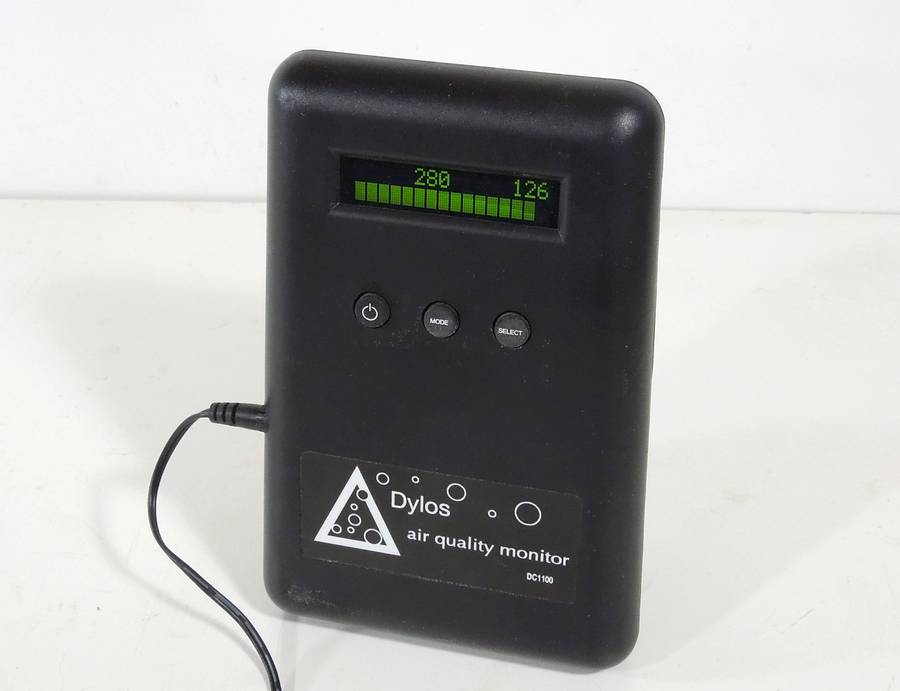
**What will happen during the intervention?**

It’s really easy to take part. You’ll need to give a couple of answers to a short survey, and then someone from a local organisation will give you simple instructions on installing an air quality monitor in your house. The monitor is a small black box that you plug into the electricity, and just leave in the living room for five days. After that, you’ll be able to unplug the monitor and bring it back to the centre staff.

A few days later, a worker from the organisation will talk to you about your results. They might let you know that there aren’t high levels of particles in the air. If they show that there are high levels, the worker will talk with you about a few simple ways you can get the levels down. They’ll also ask you a few more questions for a survey.

An air quality monitor

A few weeks later, the same worker will give you the monitor again, and you’ll be asked to install it in your home for another five days. Once you’ve brought the monitor back to the centre again, the worker will be able to give you some information about how your readings have changed.

- **What is second-hand smoke?**

Second-hand smoke is all the particles that go into the air when a tobacco product is burned. That means both smoke coming off the lit end of a cigarette, as well as smoke breathed out after taking a drag. Despite the name, it doesn’t just mean the smoke you can see and smell – more than 85% of tobacco smoke is invisible and has no smell.

- **Why is second-hand smoke dangerous?**

There are lots of dangerous chemicals in second-hand smoke. Ninety are thought to cause cancer, while some cause diseases like glue ear, meningitis and cot death. These chemicals can quickly build to dangerous levels in a home or car, even after just one cigarette. And in a home, second-hand smoke can remain at dangerous levels for more than five hours. Levels can still be high everywhere in the home even if you open the windows or you only smoke in one room.

- **Why are children particularly vulnerable to second-hand smoke?**

Children have small, growing lungs, and breathe more deeply and more quickly than adults. Because of this, they can suffer more from the effects of poisonous second-hand smoke. For that reason, it’s important to keep children safe from second-hand smoke by never smoking inside the home or car.

- **What does the air quality monitor do?**

The monitor counts tiny particles of second-hand smoke in the air. This “particulate matter” is known as PM_2.5­_. If there are news stories about high levels of air pollution in cities such as London or Beijing, PM_2.5­_ will often be the focus of the problem. It is only one of the many harmful components of second-hand smoke, but is fairly simple to test for in the air.

By using the monitor to measure the levels of PM_2.5_ in a home, we can visualise these invisible particles into a graph over time. That lets us demonstrate the existence of dangerous second-hand smoke levels even when people might think a room is clear.

- **Why am I being asked to take part?**

One of the workers at a local organisation thought you might be interested in trying the intervention out and seeing results in your home. There’s no pressure, so don’t worry if you wouldn’t like to try the intervention at the minute.

**AFRESH HANDOUT 4: PERSONAL SMOKE-FREE HOME ACTION PLAN**

**XXX’s [insert name here] smoke-free home action plan**

**SECTION 1:**

My general goal is to create a smoke-free home

My specific goal

- What am I going to do?

................................................................................................................................................................. ................................................................................................................................................................. .................................................................................................................................................................

- How am I going to do it?

................................................................................................................................................................. ................................................................................................................................................................. .................................................................................................................................................................

- With whom am I going to do it?

................................................................................................................................................................. ................................................................................................................................................................. .................................................................................................................................................................

**SECTION 2:**

How will I know how I’m doing? It is important to measure and record your progress, as it motivates you when you are succeeding, and helps you work out what you can change if your plan isn’t working.

- How will I record my progress?

................................................................................................................................................................. .................................................................................................................................................................

- When will I record my progress?

................................................................................................................................................................. .................................................................................................................................................................

We will chat about how you get on at our next meeting on [insert date/time here]

Parent signature ............................................................... Date ......................................................

Worker’s signature ........................................................... Date ......................................................

**AFRESH HANDOUT 5: DIFFICULT SITUATIONS AND ‘IF-THEN’ PLANS**

**Difficult situations**

- Are there any situations that you can think of that could make it especially difficult for you to create or maintain your smoke-free home? A time or a place or a feeling that might tempt you to go back to your old behaviour? For example: on a Saturday night when my friends are over, we just want to smoke indoors. Make a list of your difficult situations: ......................................................................................................................................... ........................................................................................................................................................................................................................................................................................................................................................................................................................... .................................................................................................................................................................................................................................................................................. .........................................................................................................................................

Now make some **plans** for how to avoid these situations or make them more manageable. For each difficult situation, think of something you could do that would lower the chance of it interfering with your planned behaviour. For example, ‘on a Saturday night when my friends are over, if they want to smoke in the kitchen, I’ll remind myself that most people smoke outdoors now, rather than in the home. I’m putting my child’s health first by asking them to smoke outside when they are here.’ Fill in the table below with your difficult situations, and for each one, make an ‘if-then’ plan for coping with it.

**Difficult situations How I will avoid or cope with them**

**If**...  **Then**…

- ......................................................... .................................................................
- ......................................................... .................................................................
- ......................................................... ..................................................................
- ......................................................... ..................................................................
- ......................................................... .................................................................
- ......................................................... .................................................................
- ......................................................... ..................................................................

**AFRESH HANDOUT 6: ENCOURAGING OTHER HOUSEHOLD MEMBERS TO ASSIST WITH CREATING A SMOKE-FREE HOME**

**Raising the issue**

Most people respond well to requests for help, so don’t be afraid to ask for support in helping to make the home smoke-free. These general tips may help you to raise the issue of making your smoke-free home:

- Firstly, you should consider whether other household members are likely to be receptive to the conversation. Don’t go ahead with the discussion if you feel very uncomfortable about raising the issue with others in your home.
- Think about whether it’s the right time and place to hold the conversation.
- Listen carefully to other people’s responses, and respond to their statements calmly, openly and without judgement.
- Remind them that you are not asking them to stop smoking altogether, but to smoke outside rather than in the home.
- Emphasise that children will only fully benefit if others in the household smoke outside too.

**Sharing information**

Parents often find it helpful to show other household members their personalised feedback graph, so that they too can see PM_2.5_ levels in the home. Discussing the graph with other household members may be enough to persuade them to assist you with creating a smoke-free home.

You may also find it helpful to:

- Share and discuss facts that are most relevant to household members from Handouts 1 and 2
- Explain an advantage of other household members trying to smoke outside and ask them for any additional advantages they can think of
- Ask family members what they think/feel about their smoking in the home
- Encourage other household members to reflect on the impact of their smoking behaviour on the well-being of others in the household.

**Overcoming family barriers to creating a smoke-free home**

Some household members may talk about barriers that they have to assisting with creating a smoke-free home. In some families, for example, not everyone understands or believes the health risks to children from second-hand smoke in the home. In other families, smoking in the home is something that parents or grandparents may have done for years, and breaking a habit like this can be hard to contemplate.

You will have already discussed potential family barriers, and identified possible solutions to overcome them, with your care professional. If you have thought of any new barriers since this discussion, try and identify some possible solutions yourself.

- Rehearse methods of dealing with family barriers. This will increase your confidence about having the discussion with other household members. It also gives you the opportunity to practice your responses to questions that family members may have.
- Ask family members if there is anything preventing them from making changes, and help them to come up with their own solutions to assist with creating your smoke-free home.

Most barriers can be overcome, especially when possible solutions are discussed together. However, if it becomes clear that other household members are unable or unwilling to assist you in creating a smoke-free home, then do the best **you** can to reduce second-hand smoke levels, by continuing to take your own smoking outside. If family members see you sticking to your goal, they may change their mind.

**Planning for a smoke-free home together**

If other household members are able to assist you in creating a smoke-free home, talk with them about how they will achieve this. They may want to complete their own personal action plan, and you may want to encourage them to develop their own ‘if-then’ plans. Monitor your progress together, discuss any setbacks as they arise, and praise successes.

**Asking visitors for their support**

Have a think about strategies for asking visitors for their support in making the home smoke-free in just the same way as you have done with family members. Reinforce your decision to create a smoke-free home by having a no-smoking sticker in the home, or by the door to remind visitors.

**AFRESH HANDOUT 7: QUITTING SMOKING**

**Why quit?**

Stopping smoking is the single most important thing a smoker can do to improve their health and will immediately reduce risk of heart disease, cancer, stroke, diabetes, rheumatoid arthritis and dementia. In addition, a 20-a-day smoker paying £7.50 a packet will save over £2700 a year.

**What tools can help me quit?**

You can use lots of different tools to help you quit smoking. Different things work for different people, so if you’ve already tried one or more of the products listed below, why not try something you’ve not used before:

- Nicotine therapies, such as chewing gum, patches, lozenges and inhalers can be provided free by NHS stop smoking services
- Your GP may be able to prescribe stop smoking medicines
- Some people have used e-cigarettes to help them quit. We don’t know how effective they are, but some users have found them helpful.

**What else can I do?**

Stop smoking services offer your best chance of quitting smoking. Get the best help:

- Phone Smokeline on **0800 84 84 84** 8am – 10pm 7 days a week
- Visit [www.canstopsmoking.com](http://www.canstopsmoking.com) for help, information or to web chat with an advisor
- Make an appointment with your GP who can refer you to your local stop smoking service
- Visit your local pharmacy to talk to your pharmacist about quitting

1. US Department of Health and Human Services. The health consequences of involuntary exposure to tobacco smoke: a report of the Surgeon General. US Department of Health and Human Services, 2006. [↑](#footnote-ref-1)
2. Semple, S and Latif, N. (2014) How long does second-hand smoke remain in household air: Analysis of PM_2.5_ data from smokers’ homes. Nicotine and Tobacco Research, 16 (10):1365-1370. [↑](#footnote-ref-2)
3. Campbell-Jack, D., Hinchliffe, S. & Bromley, C. (eds). Scottish Health Survey 2014: Volume 1: Main Report. Available from: www.gov.scot/Topics/Statistics/Browse/Health/scottish-health-survey [↑](#footnote-ref-3)
4. 1 Royal College of Physicians (2010) Passive smoking and children: A report by the Tobacco Advisory Group. London: RCP [↑](#footnote-ref-4)
