# Supplementary material for: Development of a Smoke-Free Homes Intervention for Parents: An Intervention Mapping Approach
Source: Health Psychol Bull. Author manuscript; Available in PMC 2020 Apr 24. (PMC7182446; doi:10.5334/hpb.20)
Supplement: Supplementary file 8 [file EMS86099-supplement-Supplementary_file_8_.zip › Literature Search Strategies.odt]

Supplementary file 1: Needs assessment literature review search strategies 

IM Step 1: Needs Assessment – Literature Search Strategies 

1. Rapid review of the literature on behavioural interventions to reduce indoor smoking by parents

Our initial intention was to conduct this review using several search engines, however a systematic review by Brown et al (2015)1 Brown N, Luckett T, Davidson M et al. Interventions to Reduce Harm from Smoking with Families in Infancy and Early Childhood: A Systematic Review. Int J Environ Res 2015; 12: 3091-3119. 

 with a similar focus was published prior to conducting our review. Therefore, our rapid review involved synthesising findings from the identified papers in their review. 

Eligibility:

Brown et al’s1 eligibility criteria as stated in their review were: 1) empirical study reports of interventions aimed at promoting a smoke-free home environment; 2) primary carers (parents, guardians, foster carers or grandparents) involved in the parenting of infants and young children. Where child age range exceeded 0–5 years, a mean age within the 0–5 year range was used as a criterion. 3) Included papers were published between 2000 and 2014 in peer reviewed journals to ensure a focus on the most recent research in the topic. 4) Papers were excluded if they were not written in English. 

In addition to these eligibility criteria, those set out in Table 1 also had to be met for the requirements of our rapid review:  

Table 1: Additional eligibility criteria

| Population/problem | Intervention | Outcome | Study design |
| --- | --- | --- | --- |
| Parents who smoke/SHS | Any intervention that has SHS as a primary or secondary outcome. | Changes in indoor smoking behaviour by parents. Should have a subjective and/or objective measure of SHS. | Randomized Controlled Trials (RCT’s) |

Information Sources: 

Electronic databases searched by Brown et al.1 included; MEDLINE, Cochrane Database of Systematic Reviews, PubMed, and CINAHL. Search terms included “cigarettes”, “smoking”, “tobacco”, “parent”, and “family”, as well as terms aimed at identifying intervention studies. The reference lists of included studies were searched manually by Brown et al.20 

Twelve papers were reviewed for interventions with a focus on second-hand smoke reduction (SHSr) by Brown et al.20 Five of these papers were excluded from the current rapid review as they did not meet our eligibility criteria (see Table 2 for exclusions). Our rapid review included an additional paper which Brown et al.20 identified as a smoking cessation paper, however on closer examination this paper also focussed on SHSr. Therefore, eight papers in total were included in this rapid review. 

Table 2: Exclusions

| Reference | Reason for exclusion |
| --- | --- |
| Fossum et al. 20042 Fossum B, Arborelius E, Bremberg S. Evaluation of a counseling method for the prevention of child exposure to tobacco smoke: An example of client-centered communication. Prev Med 2004; 38:295–301. | SHSr or indoor smoking was not measured therefore outcome eligibility not met. |
| Chan-Yeung et al. 20003 Chan-Yeung M, Manfreda J, Dimich-Ward H et al. A randomized controlled study on the effectiveness of a multifaceted intervention program in the primary prevention of asthma in high-risk infants. Arch Pediatr Adolesc Med 2000; 154: 657–663. Becker et al. 20044 Becker A, Watson W, Ferguson A et al. The Canadian asthma primary prevention study: Outcomes at 2 years of age. J Allergy Clin Immunol 2004; 113:650–656. Chan-Yeung et al. 2005.5 Chan-Yeung M, Ferguson A, Watson W et al. The Canadian childhood asthma primary prevention study: Outcomes at 7 years of age. J Allergy Clin Immunol 2005; 116:49–55. | Does not state how data was measured therefore outcome eligibility criteria not met. No specific results or measurements given for SHSr therefore outcome eligibility criteria not met. No results given for SHSr measurements therefore outcome eligibility criteria not met. |
| Huang et al. 20136 Huang CM, Wu HL, Huang SH et al.  Transtheoretical model-based passive smoking prevention programme among pregnant women and mothers of young children. Eur J Public Health 2013; 23: 777–782. | Focus was on intention to reduce SHS rather than actual behaviour change and no measurement of actual/perceived SHS change in the home was recorded, therefore intervention and outcome eligibility criteria not met. |

2. Rapid review of interventions that use feedback of objectively assessed data on health behaviour change

The focus of the second rapid review was to extract successful/frequently used strategies of successful feedback interventions to change health behaviour. The findings could in turn, guide the development of a practical intervention to reduce second-hand smoke in homes using an air-quality feedback instrument. Three well-defined health behaviours where feedback is commonly used were selected;  smoking cessation (not including smoking reduction); medication adherence (MA), and physical activity (PA). 

Eligibility: 

The eligibility criteria used in this rapid review included: 1) empirical study reports of interventions aimed at promoting a specific health behaviour through the use of objective feedback, 2) study reports which found the intervention to be effective, 3) RCT’s or Pilot RCT’s, 4) study reports that provided a comprehensive description of the intervention. Included papers were published between 2001 and 2015 in peer reviewed journals to ensure a focus on the most recent research in the topic. Papers were not considered if they were not written in English. 

Information Sources: 

Cochrane Reviews are widely recognised to be the gold standard in the synthesis of health relevant evidence. Consequently, the search for reviews was entirely focussed on the Cochrane Database of Systematic Reviews. Search terms for the smoking cessation element included “carbon monoxide” and “feedback”. A review by Bize et al.7 Bize R, Burnard B, Mueller Y et al. Biomedical risk assessment as an aid for smoking cessation (Review). The Cochrane Collaboration 2012; 12. 

 was selected to extract adequate studies, as their subject focus was relevant to our rapid review. The authors found that out of the fifteen trials that were included in the review, only two feedback interventions8 Bovet P, Perret F, Cornuz J et al.  Improved smoking cessation in smokers given ultrasound photographs of their own Atherosclerotic plaques. Prev Med 2002; 34:215-220.

 9 Parkes G, Greenhalgh T, Griffin M et al. Effect on smoking quit rate of telling patients their lung age: The step2quit randomised controlled trial. BMJ 2008; 336:598-600.      

 were found to be effective in increasing smoking cessation rates. These two studies were therefore included in our rapid review. 

For the medication adherence element of the current review, no systematic reviews were found specifically investigating trials which used objective feedback as part of the intervention. Thereafter, we conducted a manual search on Cochrane for individual trials using the search terms: "medication adherence" OR "adherence enhancing" AND "feedback intervention.” Two relevant studies were identified which were used in our review.10 De Bruin M, Hospers HJ, van Breukelen GJP et al. Electronic monitoring-based counselling to enhance adherence among HIV-infected patients: A randomized controlled trial. Health Psychol 2010; 29:421-428.  

 11 Ruppar TM. Randomized pilot study of a behavioural feedback intervention to improve medication adherence in older adults with hypertension. J Cardiovasc Nurs 2010; 25: 470-479.

No systematic reviews could be found which demonstrated that pedometer based interventions successfully increased PA. We then conducted a manual search on Cochrane for individual trials using the search terms: “physical activity AND feedback” and one relevant study12 De Blok BMJ, de Greef MHG, ten Hacken NHT et al. The effects of a lifestyle physical activity counselling program with feedback of a pedometer during pulmonary rehabilitation in patients with COPD: A pilot study. Patient Educ Couns 2006; 61:48-55.

 was identified. A manual search was then conducted on the University of Aberdeen’s Primo Central search engine using the search terms: “physical activity AND feedback intervention” whereby one more study13 Van Hoye K, Boen F, Lefevre J. The impact of different degrees of feedback on physical activity levels: A 4-week intervention study. Int J of Environ Res 2015; 12, 6561-6581. 

 was identified for inclusion. 

3. Review of data from previous qualitative research regarding reducing SHS in the home

The main objective of conducting the review of qualitative data was to establish: the current level of awareness/knowledge of SHS exposure; strategies/enablers to reduce SHS exposure in the home, and barriers to reducing SHS exposure in the home. 

We initially extracted information regarding relevant determinants of indoor smoking from existing qualitative research.14 Wilson I, Ritchie D, Amos A et al. 'I'm not doing this for me': mothers' accounts of creating smoke-free homes. Health Educ Res 2013; 28:165-78.

 15 Rowa-Dewar N, Amos A, Cunningham-Burley S. Children’s perspectives on how parents protect them from secondhand smoke in their homes and cars in socioeconomically contrasting communities: A qualitative study. Nicotine Tob Res 2014; 16:1429-1435. 

 16 Phillips R, Amos A, Ritchie D et al. Smoking in the home after the smoke-free legislation in Scotland: qualitative study. BMJ 2007; 335: 553.    

 17 Rowa-Dewar N, Lumsdaine C, Amos A. Protecting children from smoke exposure in disadvantaged homes. Nicotine Tob Res 2015; 17: 496-501. 

 All of the included studies took place in disadvantaged areas of Scotland with individuals who smoked in the home and/or who lived with people who smoked in the home, therefore making the results relevant to our target users of the intervention.  

During the course of our work, Passey et al (2016)18 Passey ME, Longman JM, Robinson J et al. Smoke-free homes: what are the barriers, motivators and enablers? A qualitative systematic review and thematic analysis. BMJ Open 2016; 6: e010260. 

 published the first systematic review and thematic analysis of the qualitative literature exploring the barriers, motivators and enablers of establishing and maintaining smoke-free homes.  Therefore our review also involved synthesised findings from the identified papers in their review. 

Eligibility:

Passy et al’s18 eligibility criteria as stated in their review were peer reviewed articles that: 1) were published in the English language, from 1990 to April 2014; 2) used qualitative data collection methods; 3) explored participants’ perspectives of home smoking behaviours, and the barriers, motivators and enablers to initiating and/or maintaining and smoke-free home. 

Information Sources: 

Electronic databases searched by Passey at el.18 included; MEDLINE, PsychINFO, Informit Online-Health (excluding sports science and HIV), CINAHL, Global Health, Web of Science, EMBASE and EBM Reviews: Cochrane Database of Systematic Review. As qualitative articles are poorly indexed, hand-searches were also conducted, for example within nine key tobacco, sociological and qualitative research journals, and key author searches were undertaken. 

Passey et al18 reviewed 22 papers reporting on 18 studies, from the UK (n=10), Australia (n=4), the USA (n=3), Canada (n=3) and China (n=2). We identified two further studies from Scotland which were included in this review.

References
