# Supplementary material for: Development of a Smoke-Free Homes Intervention for Parents: An Intervention Mapping Approach
Source: Health Psychol Bull. Author manuscript; Available in PMC 2020 Apr 24. (PMC7182446; doi:10.5334/hpb.20)
Supplement: Supplementary file 8 [file EMS86099-supplement-Supplementary_file_8_.zip › Statement re ethics application and letter confirming ethical approval.odt]

Statement re: ethics application document and letter confirming ethical approval

We have been unable to provide our ethics application and letter confirming ethical approval as this process was conducted via our University portal. Our study protocol, which we have provided, is very similar to the one that was uploaded as part of our ethics application.
