# Supplementary material for: Development of a Smoke-Free Homes Intervention for Parents: An Intervention Mapping Approach
Source: Health Psychol Bull. Author manuscript; Available in PMC 2020 Apr 24. (PMC7182446; doi:10.5334/hpb.20)
Supplement: Supplementary file 8 [file EMS86099-supplement-Supplementary_file_8_.zip › Step 4 interview schedule health and care professionals.odt]

Finding ways to reduce second-hand smoke exposure in homes and cars 

SEMI-STRUCTURED INTERVIEW WITH HEALTH/CARE PROFESSIONALS 

1. Introduction

We are keen to hear your views on air quality monitoring and the practicalities and challenges of using this approach within routine practice. There are no right and wrong answers. This is really just about your opinions. Your answers will be collated and summarised as part of a larger number of interviews. Your responses will remain anonymous. 

2. What are your general views on using air quality monitors with parents? (i.e. is this a useful thing to do, is it effective, what kinds of impacts does it have?)

3. Do you use air quality monitors with all clients, with some clients or with only a few clients? Tell us more about the characteristics of individuals you use air quality monitors with.

4. Do you feel you have enough information to use air quality monitors effectively? 

5. What do you think are the barriers to parents/carers creating a smoke-free home? How can these barriers be overcome?

6. To what extent do you think graphs are helpful in explaining air quality levels to parents/carers?

6b. How could graphs be improved/made more user-friendly?

6c. Do you think that the graphs are easy to understand? 

6d. Do you feel comfortable explaining the content of the graphs to clients?

7. Are there any challenges associated with measuring air quality in parent/carers homes in practice? 

8. Do parents/carers understand what PM2.5 means? Are you comfortable explaining this to them?

9. Do you have any suggestions for improving our current air quality feedback templates? 

10. Did you receive training on using air quality monitoring techniques with parents/carers? If yes, did the training meet your needs? If no, would you have liked to receive training in this area? 

11. Have you encountered any problems with the air quality monitor hardware?

12. Have you encountered any problems with the new air quality software?	

13. Any other comments or questions?
