# Supplementary material for: Development of a Smoke-Free Homes Intervention for Parents: An Intervention Mapping Approach
Source: Health Psychol Bull. Author manuscript; Available in PMC 2020 Apr 24. (PMC7182446; doi:10.5334/hpb.20)
Supplement: Supplementary file 8 [file EMS86099-supplement-Supplementary_file_8_.zip › Step 4 parent focus group consent form.odt]

Consent form

“Views on ways to encourage families to create smoke-free homes”

Please put your initials in each box to confirm:

- I confirm that I have read and understand the information sheet provided for this study. I have had the opportunity to consider the information, ask questions and have had these answered satisfactorily.

- I understand that my participation in this study is voluntary and that I am free to withdraw at any time, without giving a reason.

- I understand that information given in this focus group/interview may be used by the research team in future publications, reports or presentations.

- I understand that any personal data that could be used to identify me will be removed from the transcript of the focus group/interview and that I will not be identified in any publications, reports or presentations.

- I agree that the focus group/interview will be digitally recorded and then transcribed. 

Participant’s Name (Printed): ________________________				

Participant’s signature:  _________________________     Date: __________

Researcher’s signature: _______________________      Date: __________

Thank you for agreeing to take part in this study.

Your contribution is very much appreciated.
