# Supplementary material for: Development of a Smoke-Free Homes Intervention for Parents: An Intervention Mapping Approach
Source: Health Psychol Bull. Author manuscript; Available in PMC 2020 Apr 24. (PMC7182446; doi:10.5334/hpb.20)
Supplement: Supplementary file 8 [file EMS86099-supplement-Supplementary_file_8_.zip › Step 4 parent focus group information sheet.odt]

“Views on ways to encourage families to create a smoke-free home”

Do you have a child/children aged 5 or under?

Do you, or somebody else who lives with you, smoke?

Would you like to take part in a study about smoking in homes?

We would like to invite you to take part in a research project. This information sheet describes the project and explains what will be involved if you would like to take part.

What is the aim of the study?

We would like to hear your thoughts about a project we’ve been working on.

Most parents know that second-hand smoke (passive smoking) is harmful to children’s health. Some parents have a smoke-free home, and only smoke outside. Other parents find it difficult to make their home smoke-free.  

We’ve been helping parents who smoke indoors to learn more about levels of second hand smoke in their home, using air quality machines (pictured right). The machines let you see if there are sometimes dangerous levels of second hand smoke in your home. 

An air quality monitor

We’ve been giving parents feedback on their air quality using different types of information, including a graph which shows the amount of smoke in their home over time. We want to find out more about the best ways of giving parents this information, and we’d like to hear your views on this. 

What will taking part involve?

If you agree to take part, you will be invited to a small group discussion with other parents at the [NAME OF CENTRE]. If you are unable to attend this discussion we can arrange to interview you separately at the centre. 

We will show you some of the different types of information that we give parents to show their levels of second hand smoke in the home. We will also share with you some new ways that this information could be given. You will be asked your views on how easy it is to understand the information we share with you and how it could be improved. 

The discussion will take place at a time to suit you. It will last no longer than 1 hour. I would like to tape the discussion so I don’t need to take notes, but if you would prefer it not to be recorded I’ll take notes instead.  

 

At the end of the discussion, you may be asked if you would like to take part in the second part of the study, a few months later. This second part will help us to test out new ways of giving parents information on second-hand smoke levels in their home, to see whether the information actually helps parents to create a smoke-free home. Agreeing to take part in the discussion does not mean you have to take part in the second part of study.  

Do I have to take part?

No, taking part is voluntary.  If you would prefer not to take part you do not have to give a reason. If you take part but later change your mind you can withdraw at any time.

## What happens to any information that you obtain?

The University complies with the Data Protection Act and all information will be treated with the strictest confidence. We will record the focus group, but everything you say will be kept confidential. Your name and contact details will be kept separately from the recording and the written record of it. Any details that could be used to identify you will be removed from the written record and any quotes in written work will be entirely anonymous.

All electronic data will be stored on a password protected computer. Any paper copies will be kept in a locked filing cabinet in my office. All digital recordings will be destroyed after completion of the project. Other data from the study will be retained, in a secure location, for 7 years. 

Will you pay my expenses?

If you need to travel to get to and from the focus group, we will pay for buses, trains or taxis you use, or mileage if you come by car. We can pay up to £20, but we will need receipts for public transport.

What are the possible benefits of taking part?

There will be no immediate benefits for you, but by taking in part in this study you can help us better understand ways to help parents create a smokefree home. You may also learn from new things about protecting your own family from second-hand smoke in the home. To thank you for taking the time to talk to me, I would like to give you a £10 shopping voucher for taking part in the small group discussion.  You will also receive a £15 voucher if you take part in the second part of the study, which takes place a few months later. 

What are the possible risks of taking part?

There is no known risk if you take part in this study. 

Where can I get more information? 

If you have any questions about the project, please don’t hesitate to ask.  My contact details are: ------------------------------------------

Project team:	-------------------------------------

The study is funded by the Medical Research Council. 

Thank you for taking the time to read this information. If you are willing to be interviewed for this research project, please tell a member of staff at the [NAME OF CENTRE} or text me on ------------------------.
