# Supplementary material for: Development of a Smoke-Free Homes Intervention for Parents: An Intervention Mapping Approach
Source: Health Psychol Bull. Author manuscript; Available in PMC 2020 Apr 24. (PMC7182446; doi:10.5334/hpb.20)
Supplement: Supplementary file 8 [file EMS86099-supplement-Supplementary_file_8_.zip › Step 4 parent focus group topic guide.odt]

Finding ways to reduce second-hand smoke exposure in homes and cars 

FOCUS GROUP TOPIC GUIDE (1) 

1. Introduction

So we want to hear your views on this project that we’re working on. We’ve been measuring air quality in parents’ homes, to help them learn more about their levels of SHS. To do this, we use the machine, the air quality monitor. We give parents feedback on the air quality levels in their homes, and we do this using a combination of different graphs/visual things, and we give them some other information as well. And so that’s what we’d like your views on today. There are no right and wrong answers. This is really just about your opinions. 

       

- Smoking status

- Firstly, can I just check, is everyone here a current smoker? Is anybody here trying to quit at the moment?

- Does anyone here have a smoke-free home? (clarify that smoking is only allowed outdoors, with external doors shut) 

- Views on the air quality feedback currently used

- When we measure air quality in the home, we give parents graphs to show their air quality levels, and additional information which I’ll share with you in a minute. 

Firstly, I’d like to hear your views on the graphs that we currently use with parents [SHOW MOCK UP GRAPHS FOR 24 HOUR EXPOSURE LEVELS (FIGURE 1) AND WEEK LONG EXPOSURE LEVELS, AND EXPLAIN THEM TO PARTICIPANTS. ANSWER ANY QUESTIONS]

- How easy/difficult do you find it to understand this information?

- If I wasn’t here to explain the graphs to you, would you have been able to understand them by yourself? 

- What do you think parents would find useful about the graphs?

- Do you find parents would find the one day or one-week graph more helpful in understanding air quality levels? Why?  

- Do you think that the graphs would encourage parents to create a smoke-free home? 

- Do you think that graphs like this would encourage you to create a smoke-free home?

- Is there anything else you’d like to say about the graphs that I’ve showed you? 

- When we measure air quality in the home, we also give parents the following additional information on their air quality levels [SHOW FIGURE 3]: 

  - the average PM2.5 levels in the home

  - the maximum PM2.5 level in the home

  - The total time that air quality levels are higher than WHO guidance levels

  - The total time that particle levels are above the average levels found in Scottish bars before they became smoke-free 

- How easy/difficult do you find it to understand this information?

- If I wasn’t here to explain it to you, would you be able to understand it by yourself? 

- What do you think parents would find useful about this information? 

- Is there any part of the information that you think would particularly stand out for parents?

- Is there any part of the information that would particularly stand out for you? 

- Is there any part of the information that you think would encourage parents to create a smoke-free home?   

- IS there any part of the information that you think would encourage you to create a smoke-free home? 

- Do you think this information should be given to parents AS WELL as the graphs I showed you, or not? 

- Is there anything else you’d like to say about the information I’ve shown you? 

- Views on alternative approaches to air quality feedback

- We’d like to hear your views on other ways that we could provide parents with air quality feedback in the future, and I have some other types of air quality feedback to show you that I’d like your opinions on

SHOW FIGURES 4 AND 5, AND FOR EACH ONE ASK THE FOLLOWING SET OF QUESTIONS:

- What do you think this image is showing you?

- Do you think this image is easy to understand?

- How could this image be improved?   

- Comparing alternative feedback approaches with the approach that is currently in use

- You’ve seen some different approaches to feeding back air quality data today. Firstly, I showed you the graphs and information that we currently give to parents. Then I showed you some new possible ways of feeding back to parents on air quality levels in the home.  Of all the information that you’ve seen today:

- Which type of information do you think is the easiest to understand?

- Which type of information do you think would have the most impact on people? 

- Which type of information do you think would be most likely to encourage people to create a smoke-free home? 

6. Using graphs and information for goal setting 

- We’re keen to run a study where we measure air quality in people’s homes, and using the graphs, set goals for people to try and achieve better air quality levels. We’ve put together some graphs to show you what we mean….(SHOW GRAPHS)

- What do you think of this sort of approach?

- Do you think it would motivate people to reduce SHS levels in their home, or not? Why?

- Are there any other ways you think we could go about helping people to reduce the SHS levels in the home? 

- Conclusion

- Begin by summarise the key points discussed, then say:

- I wondered if you’ve got anything else you’d like to add, or ask me about? 

- Would you be interested to take part in a small study that will test out the best way of presenting this information?  Parents that agree to take part will have their air quality measured on two separate occasions, about 1-2 months apart. (If yes, mention voucher and take contact details. If no, say that’s fine, and we respect their decision) 

FIGURE 1: MOCK UP GRAPH FOR 24 HOUR EXPOSURE LEVELS

C:\Users\rco1\Papers written\AFRESH Health Psychology Bulletin\Figure 3 anon.png

FIGURE 2: MOCK UP GRAPH FOR PART-WEEK EXPOSURE LEVELS

C:\Users\rco1\Papers written\AFRESH Health Psychology Bulletin\Disclosure Package\Figure 2 anon .png

FIGURE 3:ADDITIONAL INFORMATION GIVEN ON AIR QUALITY LEVELS

C:\Users\rco1\Papers written\AFRESH Health Psychology Bulletin\Disclosure Package\Figure 4 anon.png

FIGURE 4: ALTERNATIVE APPROACH TO AIR QUALITY FEEDBACK (1)

C:\Users\rco1\Papers written\AFRESH Health Psychology Bulletin\AFRESH disclosure package Nov 18\Figure3_1.jpg

FIGURE 5: ALTERNATIVE APPROACH TO AIR QUALITY FEEDBACK (2)

C:\Users\rco1\Papers written\AFRESH Health Psychology Bulletin\AFRESH disclosure package Nov 18\Figure3_2.jpg
