# Supplementary material for: Development of a Smoke-Free Homes Intervention for Parents: An Intervention Mapping Approach
Source: Health Psychol Bull. Author manuscript; Available in PMC 2020 Apr 24. (PMC7182446; doi:10.5334/hpb.20)
Supplement: Supplementary file 8 [file EMS86099-supplement-Supplementary_file_8_.zip › Study Protocol.odt]

FINDING WAYS TO REDUCE SECOND-HAND SMOKE EXPOSURE IN HOMES: THE AFRESH PROGRAMME 

STUDY PROTOCOL

Objective

To create a smoke-free environment for children of parents who smoke, by having parents exchange ineffective strategies to create a smoke-free environment (e.g. smoking in another room in the house) for effective strategies (e.g. smoking outside, delaying smoking, quit smoking). 

| Investigators |  |
| --- | --- |
| Dr Sean Semple Senior Lecturer Respiratory Group, Division of Applied Health Sciences University of Aberdeen Dr Steve Turner Senior Lecturer (Clinical) Respiratory Group, Division of Applied Health Sciences University of Aberdeen Professor Marijn de Bruin Chair in Health Psychology Division of Applied Health Sciences University of Aberdeen |  |
| Dr Rachel O’Donnell Research Assistant Scottish Centre for Indoor Air, Child Health University of Aberdeen Ruaraidh Dobson Research Assistant Scottish Centre for Indoor Air, Child Health University of Aberdeen |  |
|  |  |
|  |  |
|  |  |

Study Synopsis

| Project Title | Finding ways to reduce second-hand smoke exposure in homes: The AFRESH programme |
| --- | --- |
| Sponsor name | Medical Research Council |
| Purpose of study | The health effects of exposure to second-hand smoke (SHS) are substantial. Most smokers try to protect their children from SHS, but apply strategies that are insufficiently effective. Previous work by the study team (e.g. REFRESH) has demonstrated that providing smoking parents with objective information about the effect of their behaviours on their home’s air-quality changed their behaviour and reduced children’s SHS exposure. Barriers to this approach were instrumentation cost and the labour-intensive method of intervention delivery, which involved home-visits to install and retrieve air quality monitoring devices. Recent work has identified an air-quality monitor that is cheap and can be self-installed at home by the user. This study seeks to determine how best to incorporate this new device in a simple package that can be used by NHS and non-NHS smoking intervention workers to encourage parents who smoke to make their home smoke-free. The project will involve three work-packages: (1) development of a theoretical framework for an intervention to promote parental self-management of home air quality using the air quality monitor, and identify the optimal way of intervention delivery; (2) work with the manufacturers of the device to refine the instrument to present the data in plots that can be easily interpreted by parents; (3) a feasibility study to evaluate intervention acceptability, likely rates of recruitment and effect size to examine the potential of a future large-scale trial. Throughout the project we will expand our partnership with key stakeholders responsible for the future adoption, delivery and sustainability of the program. |
| Study Design | An early phase design with three linked workpackages (WPs). 15 months. WP1 will involve the development of a theoretical framework for an intervention to promote parental self-management of indoor air quality, the identification of the optimal way of intervention delivery, and the development of the (preliminary) intervention materials including a user manual and module for training workers involved in providing advice about SHS. In WP2 work will be carried out to refine the software capability to produce the output in a format that fits the intervention strategy. We will also finalise delivery systems, including packaging. Parents who have and haven’t had air quality monitored in their home before will take part in focus groups and individual interviews to help identify the optimal way of presenting air quality feedback data to parents.    This will effectively co-developing an air quality feedback intervention, the feasibility of which will be tested in WP3, to provide the data necessary for preparing a large trial. |
| Study duration | 15 months |
| Sample Size | WP1 - Up to 10 health and social care professionals will take part in focus groups/individual interviews. WP2 – Up to 15 disadvantaged parents will take part in focus groups/qualitative interviews. This sample will include parents who have and haven’t had air quality monitored in their home before. WP3 – Up to 30 disadvantaged parents and up to 10 health and social care professionals will take part in the intervention feasibility trial, which will involve focus groups (health and social care professionals) and qualitative interviews (parents). |
| Summary of eligibility criteria | Inclusion criteria (all workpackages): Parents will be: Aged 18 or over. Fluent in English. Living in their own home, with a child/children under the age of 5 A current smoker, or living with someone else who smokes Living in a disadvantaged area of Lanarkshire or Edinburgh Exclusion criteria: 1. Unable to give informed consent.  This will be guided by Healthy Valley/Greendykes workers. |
| Sample recruitment | WP1: Health and social care professionals will be recruited through existing contacts of the research team. The sample will be selected to include professionals who have and haven’t had previous experience of using Dylos air quality machines. WP2: Recruitment of parents will take place at Healthy Valleys in Lanark, and at Greendykes Early Years Centre in South Edinburgh.  We will negotiate access with the necessary gatekeepers then arrange to visit the organisation to introduce the study. Workers will assist us in identifying smoking parents based on pre-defined inclusion criteria, through existing relationships within their community groups. Information sheets will be given to all potential participants informing them about the study and inviting them to participate in an interview/focus group (WP2) and the feasibility intervention (WP3). WP3: Up to ten workers involved in delivering the intervention at Healthy Valleys and Greendykes will take part in focus groups. Workers will invite up to 10 parents taking part in the feasibility intervention will take part in individual interviews.  We will evaluate the percentage of smokers initiating the study but not (fully) completing the intervention, and examine the causes of attrition through interviews. |

Background

Exposure to second-hand tobacco smoke (SHS) is associated with a wide-range of ill-health outcomes that are entirely preventable[^1]. More than 85% of SHS is invisible[^2] and many smokers are unaware of the impact of their smoking on air-quality in their home[^3]. Inhaling SHS can lead to acute irritant and chronic inflammatory effects on the respiratory system, and is likely to be especially harmful during early life. Children’s exposure to SHS in the UK is estimated to cause approximately 830 healthcare consultations per day, with over 20 children per day suffering illness leading to admission to hospital1. Non-smokers who live with smokers can have high SHS exposures, particularly young children who spend much of their day at home with a smoker[^4]. Exposure to SHS is linked to a wide range of adverse health outcomes in infants, children and adults, with exposure more common in disadvantaged households[^5].

There is growing evidence of the importance and benefits of presenting complex health information in visual formats that can be understood by the target population[^6],[^7] with considerable literature exploring how those with lower education and literacy levels can be helped[^8],[^9]. Data from occupational environments also indicates that visual exposure feedback can be very effective in changing behaviour in the workplace[^10]. Previous work has demonstrated that personalized measurements of household air quality can lead to changes in smokers' smoking behaviour and that visual, graphical and numerical data can be effectively explained to a wide-variety of users3, [^11]. 

The UK government has stated that they wish to see two-thirds of smokers’ homes being smoke-free by 2020[^12] and the Scottish government tobacco control strategy has a target of reducing the number of children exposed to SHS at home by 50% by 2020[^13], [^14]. Recent work[^15] has also shown that SHS remains in household air for many hours after a cigarette is extinguished: with these data being central to a public health TV advert broadcast as part of the Scottish Government’s rightoutside.org campaign[^16]. The study is very timely and aims to provide health workers with a simple, low-cost procedure to help change smoking behaviour of parents in their house, and create a safer and healthier environment for children.

The intervention will provide air quality feedback with the aim of encouraging smokers to make their home smoke-free. Central to this behaviour change intervention is parents’ intrinsic motivation to protect their children and be considered by others as a good parent3,11; and the importance of behavioural feedback and self-monitoring in self-regulating behaviour[^17] [^18] (provided here through the air quality monitor). Self-determination Theory[^19] and Control Theory[^20], [^21] have therefore been selected as the key theories to guide the development of our intervention. The intervention will draw upon existing resources and thus contain 1-on-1 contact between parents and workers (3rd sector/community group local champions interested in promoting smoke-free homes) trained in delivering the intervention. We will also explore the need for additional materials such as a website or leaflet. We will co-operate with smokers and stakeholders responsible for the future delivery of the intervention when developing the intervention strategy and designing the materials.  

The REFRESH project established the feasibility of using air-quality feedback to motivate smoking parents to change their behaviour to reduce their children’s exposure to SHS3, but that key barriers to implementing this approach were instrument and labour costs of delivery. Our work has established the need for a low-cost device that removed the necessity of labour-intensive home visits. A short, opportunistic study within the NHS Lanarkshire First Steps programme (which supports disadvantaged and vulnerable mothers) explored using a new air-quality device, the Dylos DC1700, to overcome many of the problems with REFRESH. This low-cost device has now been validated for measurement of SHS[^22], [^23] and, at recent workshops at the University of Aberdeen and at Action on Smoking and Health (ASH) Scotland during 2013/4, a range of NHS and non-NHS health improvement professionals have expressed interest in using this device further. Current methods of explaining air quality feedback to smokers have developed organically through the various projects we have become involved in over the past decade. There is now a need for a more structured, evidence-based approach to delivering air quality feedback. 

Aim

To produce an evidence-based method of explaining air quality feedback to smokers, which will in turn encourage parents who smoke to effectively create a smoke-free environment for their children.

Research questions

1. Intervention development: 

(a) What is the optimal way for parents to use the Dylos DC1700 for self-monitoring air quality in their home? 

	(b) What is the best way to feed back the air quality data to parents? 

(c) How best should this feedback be combined with additional motivational and goal setting techniques to create an effective intervention with sustained effects on behaviour? 

(d) How can this intervention be most efficiently delivered within the context of existing services? 

2. Intervention piloting: 

(a) What is the likely proportion of smoking parents interested in (i.e., recruitment rates) and completing this intervention (i.e., completion/attrition rates)? 

(b) What is the likely effect size of the intervention on implementing smoke-free house rules and on the air quality in the house? 

This project will seek to answer these questions through a partnership of technical scientists, public health specialists, a health psychologist, and end-users. This project will provide the main study with a finalised intervention package and a tool kit for implementing this in health-care and other settings. Moreover, it should provide information that is essential for designing a successful large-scale trial to evaluate the effectiveness and cost-effectiveness of this intervention.

Research Methods

1. Intervention development: 

WP1 (addressing RQ 1c & 1d) will commence with a rapid review of the literature on behavioural interventions to reduce indoor smoking by parents; and of interventions that use feedback of objectively assessed data on behaviour or behavioural outcomes. These findings will be combined with the data from previous qualitative research, and Self-regulation and Control Theory, to: (a) develop a theoretical model explaining the key determinants of parental behaviour change (i.e., exchange ineffective for effective strategies for improving indoor air quality), and potent behaviour change techniques[^24] for changing these; (b) identify the most efficient routes for delivery of the intervention; and (c) design and pre-test the intervention materials. Parents who smoke and smoking intervention workers will be closely involved in this process through focus group and one-to one interviews (intervention co-creation), to ensure a match with people’s existing levels of knowledge, norms and values, motivation, capabilities, and context. 

WP2 (addressing RQ 1a & 1b) will use the same focus groups and individual interviews to identify what elements of home air-quality information feedback are considered to be most useful, how this information can best be presented graphically and numerically, and any practical constraints with using the instrument. This co-production approach will ensure that the feedback material is understandable and acceptable to both smoking intervention workers and smokers. WP2 will also include the development of a training session and technical manual for smoking intervention workers and information for smokers on how to operate the device. 

Parents – focus groups/qualitative interviews 

Within WPs1 and 2, we propose to interview up to 20 disadvantaged parents who either smoke, or live with someone who smokes. They will also have a child/children under five. 

This sample will comprise of participants from Healthy Valleys and Greendykes centres. 

Parents will be recruited via gatekeepers from Healthy Valleys (Lanark), and Greendykes (South Edinburgh). Gatekeepers will identify potential participants, distribute invitation letters and information sheets (see Appendices 1-2) to them and liaise with the researcher to set up suitable focus group/interview times and dates. If preferred, a member of the research team will visit each centre to speak with staff and parents about the study, and then distribute information sheets to smoking parents that may be willing to take part in the study. There will be a minimum of 48 hours between the information received and the consent (Appendix 3) and interview.

We do not aim to recruit a representative population sample for this phase of the study. It is likely that some of these participants will have established some smoking restrictions in their own homes, but others may have experienced certain barriers in doing so (for example, relating to sole-parenting, lack of outdoor home space, or lack of motivation). 

Smoking intervention workers – focus groups/individual interviews

Within WPs1 and 2, we will conduct panel discussions with smoking intervention workers to seek to identify their views on what elements of home air-quality information feedback are considered to be most helpful, how this information can best be presented graphically and numerically, and any practical constraints with using the Dylos instrument. This research team will utilise existing contacts for this element of the work, inviting workers to take part who have extensive, some and no prior experience of using the Dylos machines. 

2. Intervention piloting: 

WP3 (addressing RQ 2a & 2b) will be a 6-month feasibility study, with 2 months recruitment and 2 months follow-up, followed by 2 months data analyses and report writing. We plan to conduct this feasibility study with parents and health and social care professionals associated with Healthy Valleys (Lanark) and Greendykes Early Years Centre (South Edinburgh). We will seek to trial the intervention with up to ten community based workers and 25-30 parents. 

Some parents and workers taking part in the intervention pilot will have volunteered to being contacted about this during their interview/focus group associated with WPs 1 and 2 (but can still withdraw from this phase of the research at any time). Workers involved in delivering the intervention will identify additional smoking parents based on pre-defined inclusion criteria, through existing relationships within their centre. When re-contacting parents (who have previously agreed to be contacted to be asked about taking part in the intervention pilot), if their telephone number is no longer valid we will contact the relevant Centre and ask them to contact the client on our behalf. 

All workers will receive our half day training course and written guidance (see Appendix 4) outlining the aims of the project and information on how to recruit participants. Training will also be provided on SHS advice and how to use the Dylos machine to support this advice (provided by ASH Scotland and University of Aberdeen). 

All parents will be invited to take part in the study with study information sheets provided (see Appendix 5). Written informed consent will be gained from those taking part (see Appendix 6). 

Participants regularly visit the Centre and air quality discussions will be built in to the existing contact with the worker, so there will be little additional load on workers’ time. Participants will bring monitors back into the centre once they have had them in their house for a week, and they will be collected from the centre by a member of the research team.  

Recruited families will trial the use of the Dylos device and receive the new air quality feedback package co-developed in WPs 1 and 2. Alongside air-quality monitoring, short interview-administered questionnaires to determine changes in smoking, household rules and quit attempts will also be administered to both groups at baseline and 2-month follow-up (see Appendices 7-8). All air quality monitoring devices will be wiped down with an alcohol wipe after each installation to address any infection control issues. Up to 10 parents will be invited to take part in an individual interview at the end of the intervention (see Appendix 9), which will explore their experiences of taking part in the intervention pilot, and the impacts of the feedback they received on their smoking behaviour in the home. Verbal consent to record each interview will be sought before it commences.  Centre workers will also be invited to take part in a focus group at the end of the intervention (see Appendix 10), to explore their experiences of intervention delivery. This may be delivered in an individual interview format if staff prefer. Verbal consent will be sought to record the focus groups/interviews before they commence.

The numbers of participants in the intervention pilot have been selected in order to include a diverse range of environments and personal contexts, whilst enabling data collection and analysis in the time available. We will evaluate the percentage of smokers initiating the study but not (fully) completing the intervention, and examine the causes of attrition through interviews. 

Ethical Approval has been obtained from the University of Aberdeen College Ethics Review Board (CERB). 

Data collection

Our primary study measure will be objective air quality measures, and secondary outcomes will be self-reported uptake of smoke-free rules and several behavioural determinant measures (e.g., knowledge, risk perception, motivation, capability). Pre-post measures will be used to estimate the likely effect of the intervention on indoor air quality, which can then be used based on the literature to estimate the health benefits for family members including the children.  Process measures will include fidelity of intervention delivery and satisfaction with the intervention from smokers and professionals. This will also involve focus groups with smoking intervention workers for refining the participant inclusion strategies, measures, and intervention (materials, training). Interviews with parents will also focus on evaluating and where necessary improving study procedures and (intervention) materials.  Individual interviews and focus groups have been demonstrated to be effective means by which to engage people in research on this topic area. Interviews will take place at a time and location agreed by each parent and the relevant gatekeepers. Following the WP1/2 parent focus groups/interview, participants will be offered a high street shopping voucher of £10 to thank them for giving their time. Parents will be given a £15 voucher if they participate in the intervention pilot (WP3), and a further £10 voucher if they participate in a qualitative interview at the end of the pilot. 

The parent interviews and professionals’ focus groups/interviews associated with WPs 2 and 3 will all follow topic guides (Appendices 14-15). Focus group sizes will be limited to 5-6 participants. We anticipate that this will allow for lively discussion and the production of rich data, without becoming unwieldy. The focus group/individual interview discussions are expected to last around 45 minutes, depending on the time participants are able to give. All interviews will be digitally recorded (with consent), and transcribed for in-depth analysis.

Confidentiality and anonymity

The researchers will explain to all participants at the start of interviews and focus group discussions that they will not discuss what is said with others, including parents and gatekeepers. They will also be assured that their name and other identifying features of what they said would be anonymised but that their words might be quoted in reports and presentations. Focus group participants will also be asked not to share what other participants said with anyone outside the group. At the start of the intervention pilot workers will also make clear to parent participants that their air quality monitoring data will remain anonymous. 

Data analysis 

Quantitative analyses will be used to determine: 

- Percentage uptake by clients invited to take part;

- Changes in household air quality as measured by PM2.5 concentrations at baseline and follow up points;

- Changes in attitudes towards the hazard presented by second-hand smoke;

- Changes in household smoking rules.

Qualitative analysis will be ongoing as data collection progresses. Data collection and analysis will take place concurrently, informed by thematic analysis, with further analyses being undertaken after data collection is completed. Data will be analysed thematically in order to address the research questions, while also considering the group dynamic and context. All transcripts will be repeatedly read and cross-compared by members of the research team to identify recurrent themes, sub-themes and issues within and across interviews and focus groups, including those not foreseen at the study’s outset. This approach will maximise rigour and counteract the possibility of researcher bias. Once key themes have been identified and agreed upon (relating to the original research questions and emergent findings) coded data sets will then be subjected to further in-depth analysis to allow for the identification of further themes and sub-themes. 

6. Timetable

This work will be completed within 17 months starting June 2015: 

| MONTHS | DURATION | TASKS |
| --- | --- | --- |
| 1-10 | Jun 15-Jan 16 | Development of a theoretical framework for the intervention (WP1) |
| Jun 15-Feb 16 | Identification of the optimal way of intervention delivery (WP1) |
| Jun 15-Mar 16 | Development of the preliminary intervention materials including a user manual and module for training workers (WP1) |
| 6-11 | Nov-Dec 15 | Ethical approval obtained from the University of Aberdeen (WP2) |
| Jan 16 | Parents recruited for interviews/focus groups (WP2) |
| Feb–Apr 16 | Interviews/focus groups with parents conducted (WP2) |
| 12-17 | May–Jul 16 | Feasibility study – recruitment and baseline air quality measure (WP3) |
| Jun-Aug 16 | Feasibility study – follow up air quality measure (WP3) |
| Sept-Oct 16 | Data analysis and report writing (WP3) |

Reporting and Dissemination

Findings from the study will be disseminated at national and international conferences, and in high impact peer-reviewed academic journals.

This project will provide a finalised intervention package and a tool kit for implementing this in health-care and other settings. Moreover, it should provide information that is essential for designing a successful large-scale trial to evaluate the effectiveness and cost-effectiveness of this intervention.   

APPENDIX 1: PARTICIPANT INVITATION LETTER 

						    Division of Applied Health Sciences

							University of Aberdeen

                                                                                                                           1.068 Polwarth Building

                                                                                                                                                 Foresterhill

                                                                                                                                  Aberdeen AB25 2ZD

Date as postmarked

Finding ways to reduce second-hand smoke exposure in homes:

The AFRESH programme

Dear (Individual’s name here)

Your [INSERT NAME OF CENTRE] worker has given me permission to invite you to take part in the above research study. 

I have enclosed further information about this study to help you decide whether you would like to take part or not.  If you would like to take part, please let your Centre worker know. We will then arrange a suitable time for our researcher to come to the Centre, to interview you for the research study.  

Thank you for your time and consideration in this matter. 

Yours sincerely

[INSERT ELECTRONIC SIGNATURE]

Sean Semple (Chief Investigator)

	

unilogo

APPENDIX 2: PARTICIPANT INFORMATION SHEET: PARENT INTERVIEWS 

Finding ways to reduce second-hand smoke exposure in homes: 

The AFRESH programme 

Do you have a child/children aged 5 or under?

Do you, or somebody else who lives with you, smoke?

Would you like to take part in a study about smoking in homes?

We would like to invite you to take part in a research project. This information sheet describes the project and explains what will be involved if you would like to take part.

What is the aim of the study?

We would like to hear your thoughts about a project we’ve been working on. 

Most parents know that second-hand smoke (passive smoking) is harmful to children’s health. Some parents have a smoke-free home, and only smoke outside. Other parents find it difficult to make their home smoke-free.  

We’ve been helping parents who smoke indoors to learn more about levels of second-hand smoke in their home, using air quality machines (pictured right). The machine measures levels of second-hand smoke in your home. 

An air quality monitor

We’ve been giving parents feedback on their air quality using different types of information, including a graph which shows the amount of smoke in their home over time. Now, we want to find out more about the best ways of giving parents this information, and we’d like to hear your views on this. 

What will taking part involve?

If you agree to take part, you will be invited to take part in a focus group at the [NAME OF CENTRE]. If you are unable to take part in the focus group, but keen to participate, we can arrange to interview you at the centre, at a time which better suits you. 

We will show you some of the different types of information that we give parents to show their levels of second-hand smoke in the home. You may remember some of this information from our previous work. We will also share with you some new ways that this information could be given. You will be asked your views on how easy it is to understand the information we share with you and how it could be improved. 

The discussion will take place at a time to suit you. It will last no longer than 1 hour. I would like to tape the discussion so I don’t need to take notes, but if you would prefer it not to be recorded I’ll take notes instead.  

 

At the end of the discussion, you may be asked if you would like to take part in the second part of the study, a few months later. This second part will help us to test out new ways of giving parents information on second-hand smoke levels in their home, to see whether the information actually helps parents to create a smoke-free home. Agreeing to take part in the focus group/interview does not mean you have to take part in the second part of study.  

Do I have to take part?

No, taking part is voluntary.  If you would prefer not to take part you do not have to give a reason. If you take part but later change your mind you can withdraw at any time.

## What happens to any information that I give you?

The University complies with the Data Protection Act and all information will be treated with the strictest confidence. We will record the focus group, but everything you say will be kept confidential. Your name and contact details will be kept separately from the recording and the written record of it. Any details that could be used to identify you will be removed from the written record and any quotes in written work will be entirely anonymous.

All electronic data will be stored on a password protected computer. Any paper copies will be kept in a locked filing cabinet in my office. All digital recordings will be destroyed after completion of the project. Other data from the study will be retained, in a secure location, for 7 years. 

What are the possible benefits of taking part?

There will be no immediate benefits for you, but by taking in part in this study you can help us better understand ways to help parents create a smoke-free home. You may also learn from new things about protecting your own family from second-hand smoke in the home. To thank you for taking the time to talk to me, I would like to give you a £10 shopping voucher for taking part in the small group discussion.  You will also receive a £15 voucher if you take part in the second part of the study, which takes place a few months later. 

What are the possible risks of taking part?

There is no known risk if you take part in this study. 

Where can I get more information? 

If you have any questions about the project, please don’t hesitate to ask.  My contact details are:

Project team:	 

The study is funded by the Medical Research Council. 

This study has been reviewed by the Ethics Review Board of the College of Life Sciences and Medicine of the University of Aberdeen. 

Thank you for taking the time to read this information. If you are willing to be interviewed for this research project, please tell your worker, or another member of staff at [NAME OF CENTRE], who will contact me so that we can arrange a suitable time to visit you. 

APPENDIX 3: CONSENT FORM: PARENT FOCUS GROUPS/INTERVIEWS 

Finding ways to reduce second-hand smoke exposure in homes: 

The AFRESH programme

#  Consent form

Please put your initials in each box to confirm:

- I have read and understand the information sheet provided for this study. I have had the opportunity to consider the information, ask questions and have had these answered satisfactorily.

- I have agreed to take part in this study. I understand that my participation in this study is voluntary and that I am free to withdraw at any time, without giving a reason.

- I understand that information given in this focus group/interview may be used by the research team in future publications, reports or presentations.

- I understand that any personal data that could be used to identify me will be removed from the transcript of the focus group/interview and that I will not be identified in any publications, reports or presentations.

- I agree/disagree that the focus group/interview will be digitally recorded and then transcribed. 

Participant’s Name (Printed): ________________________				

Participant’s signature:  _________________________     Date: __________

Researcher’s signature: _______________________      Date: __________

Thank you for agreeing to take part in this study. Your contribution is very much appreciated

APPENDIX 4: STUDY GUIDELINES FOR CARE PROFESSIONALS 

Finding ways to reduce second-hand smoke exposure in homes:

The AFRESH programme

 Guidelines for Care Professionals

General points about the AFRESH programme

Second-hand smoke (SHS) exposure is associated with a number of illnesses in children.  Pre-school children are especially at risk from SHS since they have a higher respiratory rate, spend longer periods in the home & are often unable to remove themselves from environments where they are exposed to smoke.  Parents who don’t want to or can’t stop smoking, can reduce their children’s exposure by creating a smoke-free home.  

There is evidence from other studies that providing smoking parents with objective information about the effect of their smoking on their home’s air-quality can change their behaviour and reduce children’s SHS exposure. More recent work has identified a low-cost air-quality monitor that is simple to use. During phases 1 and 2 of the current study, we identified an evidence-based approach to delivering air quality feedback to participants. This third phase of the study will now test out this new approach, to find out whether it will encourage parents who smoke to effectively create a smoke-free environment for their children. 

Aims

- To test whether this new method of providing and explaining air quality feedback to parents encourages them to effectively create a smoke-free home.  

- To examine the likely proportion of parents interested in taking part and completing this type of programme.  

- To explore the experience of participating parents of receiving this type of air quality feedback programme. 

- To explore the experience of care professionals in delivering the AFRESH programme within the context of their existing service.  

Methodology

The target group will be parents you currently support from the [NAME OF CENTRE] who have pre-school children who are currently exposed to second-hand smoke.

Participation in this study involves 5 contact sessions with each parent who takes part, spread over the course of approximately 4 weeks. We have developed an outline of the suggested structure for each contact session, together with materials to be used at each session (see separate AFRESH programme booklet). 

During contact session 1 (approx. 20 mins), you will be establishing with parents their current knowledge, belief and attitudes regarding children’s exposure to second-hand smoke in the home. You will also be asking parents about the things that may make it harder/easier for them to create a smoke-free home. At the end of session 1, you will be inviting parents to take part in the intervention pilot, giving them an information sheet with details of the study. Each parent should have at least 48 hours to consider whether or not they would like to take part before moving onto Session 2.

Session 2 (approx. 10 mins) involves confirming that parents have read the information sheet, and answering any questions they may have.  Parents who are willing to take part in the study are then asked to complete a consent form, and are given a Dylos machine to take home and install in their living room for 5 days. The Dylos machine should be returned to the centre after 5 days so that you can download the personalised data in preparation for session 3. 

Session 3 (approx. 20-60 mins) can be split into two shorter sessions if required. At this session, you will be giving each parent their personalised feedback on air quality levels in the home, and discussing ways in which their levels could be improved. This involves looking at barriers and solutions to creating their smoke-free home, and making a personal action plan for them to reduce SHS levels in the home. Session 3 also incorporates discussion on involving other household members in creating a smoke-free home, if applicable. 

Session 4 (approx. 5 mins) takes place between 2-4 weeks after session 3, and involves giving parents the Dylos machine for a second time. They will then install it in their living room for another 5 days to see whether they have been able to reduce levels of second-hand smoke in the home. As before, the Dylos machine should then be returned to the centre so that you can download the personalised data in preparation for session 5. 

Session 5 (approx. 20 mins) involves giving each parent their personalised feedback on air quality levels in the home, comparing their most recent results with those measured during Session 2. This provides an opportunity for parents to see whether they have been able to reduce levels of SHS in their home, using the personal action plan that was developed during Session 3.  

At the end of the study, we will ask you to ask parents if they would like to take part in a short interview with one of the research team from the University of Aberdeen. This will seek to explore their experience of taking part in the study. We will also ask you if you are able to take part in a short interview or focus group, in order to explore your experiences and views on the usefulness of this approach as a means of motivating parents to change behaviours or that of others, in relation to SHS.

General points for recruiting parents to the pilot intervention: 

- Be focussed on the aims of the project.

- Show understanding of their situation without agreeing that they have no other choice.

- The emphasis of the study is NOT on quitting smoking, but on reducing their child’s exposure to second-hand smoke in the home.

APPENDIX 5: PARTICIPANT INFORMATION SHEET

Finding ways to reduce second-hand smoke exposure in homes: 

The AFRESH programme

Participant Information Sheet

Do you have a child/children aged 5 or under?

Do you, or somebody else who lives with you, smoke?

Would you like to take part in a study about smoking in homes?

We would like to invite you to take part in a research project. This information sheet describes the project and explains what will be involved if you would like to take part.

What is the aim of the study?

We would like to work with parents who smoke indoors to learn more about the levels of second-hand smoke in their home, using air quality machines (pictured right). 

Most parents know that second-hand smoke (passive smoking) is harmful to children’s health. Some parents have a smoke-free home, and only smoke outside. Other parents find it difficult to make their home smoke-free.  

An air quality monitor

The machines let you see if there are sometimes dangerous levels of second-hand smoke in your home. We’d like to give you feedback on the air quality in your own home, to show you the amount of smoke in your home. We’d like to measure air quality levels in your home on two occasions, for 5 days each time, to see whether the information we give you encourages you to reduce second-hand smoke levels in your home.  

What will taking part involve?

We’d like to measure air quality levels in your home on two occasions, for 5 days each time, to see whether the information we give you encourages you to reduce second- hand smoke levels in your home.  

XX [staff name] will give you the air quality machine to take home and install in your living room. After 5 days, and once you’ve brought the machine back to the centre, XX will arrange a suitable time to explain your results with you, and discuss ways in which you could reduce second-hand smoke levels in your home, if applicable. This will include discussion of barriers that may make it more difficult for you to create a smoke-free home, and possible solutions to these barriers. You will then be asked to take the air quality machine home for a second time, and install it in your living room for another 5 days. Once you bring it back to the centre, XX will arrange a suitable time to go through your second set of results with you, to see how they compare to your first set of results, and whether levels of second-hand smoke in your home have reduced.  

At the end of the study, you may be asked if you would like to take part in a short interview about your experiences of taking part in the study. 

Do I have to take part in the study?

No, taking part is voluntary.  If you would prefer not to take part you do not have to give a reason. If you take part but later change your mind you can withdraw at any time.

## What happens to any information that I give you?

The University complies with the Data Protection Act and all information will be treated with the strictest confidence. We will record the focus group, but everything you say will be kept confidential. Your name and contact details will be kept separately from the recording and the written record of it. Any details that could be used to identify you will be removed from the written record and any quotes in written work will be entirely anonymous.

All electronic data will be stored on a password protected computer. Any paper copies will be kept in a locked filing cabinet in my office. All digital recordings will be destroyed after completion of the project. Other data from the study will be retained, in a secure location, for 7 years. 

What are the possible benefits of taking part?

There will be no immediate benefits for you, but by taking in part in this study you can help us better understand ways to help parents create a smoke-free home. You may also learn from new things about protecting your own family from second-hand smoke in the home. To thank you for taking the time to take part and to compensate you for the electricity costs of operating the monitor (approximately 10 pence per day), you will receive a £15 shopping voucher at the end of the study. You will also receive a £10 shopping voucher if you take part in an interview once the study has finished.  

What are the possible risks of taking part?

There is no known risk if you take part in this study. 

Where can I get more information? 

If you have any questions about the project, please don’t hesitate to ask.  My contact details are:

Project team:	

 

The study is funded by the Medical Research Council. 

This study has been reviewed by the Ethics Review Board of the College of Life Sciences and Medicine of the University of Aberdeen.

Thank you for taking the time to read this information. If you are willing to take part in the study, please tell XXX [staff name], or another member of staff at [NAME OF CENTRE]. 

APPENDIX 6: CONSENT FORM FOR PARENTS: PILOT INTERVENTION 

Finding ways to reduce second-hand smoke exposure in homes: 

The AFRESH programme

Consent form

Please put your initials in each box to confirm:

- I have read and understand the information sheet provided for this study. I have had the opportunity to consider the information, ask questions and have had these answered satisfactorily.

- I give permission for measurement of indoor air to be made in my home. 

- I have agreed to take part in this study. I understand that my participation in this study is voluntary and that I am free to withdraw at any time, without giving a reason.

- I understand that any information I give may be used by the research team in future publications, reports or presentations.

- I understand that any personal data that could be used to identify me will not be used in any publications, reports or presentations.

Participant’s Name (Printed): ________________________				

Participant’s signature:  _________________________     Date: __________

Researcher’s signature: _______________________      Date: __________

Thank you for agreeing to take part in this study. Your contribution is very much appreciated.

Participant ID

APPENDIX 7: BASELINE QUESTIONNAIRE FOR PARENTS TAKING PART IN THE INTERVENTION PILOT 

Finding ways to reduce second-hand smoke exposure in homes:  

The AFRESH programme

Baseline questionnaire

- Is your home

a detached house 						□

a semi-detached house 					□

a terraced house  						□

a tenement 						□

a flat 							□

If your home is a tenement or flat what floor do you live on? ____________

- Does your home have 

a private garden 						□

a shared garden 						□

no garden space 						□

a balcony	 						□

- Is smoking allowed inside your home (tick all that apply)?

Smoking is not allowed					□

Smoking is allowed in certain places only			□

Smoking is only allowed when no children are present	□

Smoking is only allowed on special occasions		□

Smoking is allowed anywhere in the home			□

Other (please specify)  □    ____________________________	

- Do you currently have a car? (Y/N)

- Can you tell us about the people you share your home with and if they smoke please estimate how many cigarettes, on average, they might smoke in the home each day? 

| Person Number | Relationship to You | Average number of cigarettes they smoke in the home each day (Tick appropriate box) |
| --- | --- | --- |
| 1 |  | Non-smoker□  < 5□  5 - 10□  10 – 20□  > 20□ Don’t know□ |
| 2 |  | Non-smoker□  < 5□  5 - 10□  10 – 20□  > 20□ Don’t know□ |
| 3 |  | Non-smoker□  < 5□  5 - 10□  10 – 20□  > 20□ Don’t know□ |
| 4 |  | Non-smoker□  < 5□  5 - 10□  10 – 20□  > 20□ Don’t know□ |
| 5 |  | Non-smoker□  < 5□  5 - 10□  10 – 20□  > 20□ Don’t know□ |

- If smoking is allowed in certain places only, could you tell us where (tick all that apply)?

Kitchen/utility room   	□			Living room/lounge	□

Dining room		□			Your bedroom		□

Attached garage	□

Other (please specify) □      __________________________

- To what extent do you agree or disagree with each of the following statement about smoking?   Please tick one box for each statement.

|  | Strongly agree | Agree | No strong opinion | Disagree | Strongly Disagree |
| --- | --- | --- | --- | --- | --- |
| Inhaling other people’s tobacco smoke poses a high risk to health |  |  |  |  |  |
| I would challenge someone smoking in a non-smoking area |  |  |  |  |  |
| The dangers of inhaling other people’s tobacco smoke are greatly exaggerated |  |  |  |  |  |
| I would ask someone who smokes to smoke outside of my house |  |  |  |  |  |
| Children are more at risk from other people’s tobacco smoke than adults |  |  |  |  |  |
| Exposure to other people’s tobacco smoke can increase the severity of asthma in children |  |  |  |  |  |
| Other people’s tobacco smoke can cause significant problems for children |  |  |  |  |  |

Date Completed: ___________________                            

Name of Centre Worker: _______________­­­­­­­­­­­­­­­­­­­­­­­­______

 

Participant ID

APPENDIX 8: FOLLOW-UP QUESTIONNAIRE FOR PARENTS TAKING PART IN THE INTERVENTION PILOT 

Finding ways to reduce second-hand smoke exposure in homes:

The AFRESH programme

Follow-up questionnaire

- Following the feedback you have received on air quality in your home, please tell us about current smoking rules inside your home (tick all that apply):

Smoking is allowed anywhere in the home			□

Smoking is allowed in certain places only			□ 

(if ticked, go to question 2)

Smoking is only allowed when no children are present	□

Smoking is only allowed on special occasions		□

Smoking is never allowed anywhere inside the home	□

Other (please specify)     ____________________________

- If you said smoking is allowed ‘in certain places only’ inside your home in question 1, please tell us where (tick all that apply). (If you did not say smoking is allowed in ‘certain places only’ in question 1, skip this question)

Kitchen/utility room		□

Dining room 			□

Living room/lounge		□

Your bedroom			□

Another adult’s bedroom	□	

Attached garage		□

Other (please specify)     ____________________________

3. Since you had first indoor air quality measurement and feedback have you changed any aspect of your smoking behaviour or smoking rules in the home?

Yes □	   No   □ 	(if yes, go to question 4, if no go to question 5)

4. Which of the following changes have you made to your smoking behaviour, or smoking rules in the home (please tick all that apply)

Smoking is allowed in fewer rooms indoors			□

Smoking is not allowed in rooms where children are 	□

Smoking is no longer allowed anywhere indoors 		□

I have reduced the amount I smoke				□
I have stopped smoking					□
Other (please specify)     ____________________________

5. Thinking about yourself, since you had your first indoor air quality measurement and feedback, have you made any of the following changes to your smoking behaviour in the presence of children?

I smoke in the presence of children inside the home...:

Not at all						□

Less than before	 				□

About the same as before 				□

More than before					□

Not applicable - I don’t smoke			□

6. Thinking about other people in your household, since you had your first indoor air quality measurement and feedback, have they made any changes to their smoking behaviour in the presence of children? 

No changes have been made				□

They smoke less than before				□

They smoke about the same as before 		□

They smoke more than before			□

Not applicable - nobody else smokes	 in the home	□

Date Completed: ___________________                            

Name of Centre Worker: _______________­­­­­­­­­­­­­­­­­­­­­­­­______

APPENDIX 9: TOPIC GUIDE FOR PARENTS TAKING PART IN THE INTERVENTION PILOT 

Finding ways to reduce second-hand smoke exposure in homes: 

The AFRESH programme

 

INDIVIDUAL INTERVIEW SCHEDULE

Interviews will be semi-structured; the order and phrasing of the questions will depend on the participant’s responses. 

1. INTRODUCTION

- Describe interview purpose: 

This interview gives you an opportunity to reflect on your experience of taking part in the study, and if applicable, your experience of making any changes in your (or other householders’) smoking practices as a result..

- Describe duration and procedure of interview: 

The interview will take approximately 45 minutes. You do not have to answer every question, and you can stop the interview at any time if you wish to. Reiterate confidentiality, non-identifiable reporting, and confirm consent to record.

- DEMOGRAPHICS

Are you living in a:

  - House

  - Flat with shared entrance (which floor and does it have a balcony?)

  - Flat with own entrance

  - Bungalow

  - Other (specify)

- Do you have a garden? (Y/N)

- What is your age?

- How many children live with you?

- What are the ages of the children living with you?

- Does anyone else live here with you? If yes, do they smoke?

- Do you currently smoke?

- THE INTERVENTION

So you’ve recently had the air quality machine in your home, on two separate occasions, to measure air quality levels in your home.  

- Were you living in the same house/flat both times your air quality was measured?

- Were the same people living with you both times your air quality was measured?  

(If no to either b or c, record what/when changes took place)

c. Were you given any of the following leaflets/handouts by the centre staff [present each material to the parent in turn], and if so, what did you think about them? (were they easy to understand, were they helpful to you or not, could they be improved, and if so how?)

 

| Material | Received? (Y/N) | Comments |
| --- | --- | --- |
| Factsheet 1: What you need to know about SHS |  |  |
| Factsheet 2: SHS |  |  |
| Handout 1: Barriers, facilitators and solutions for creating a smoke-free home |  |  |
| Handout 2: Example of personalised feedback from another parents’ home |  |  |
| Handout 3: Your guide to the air quality intervention |  |  |
| Handout 4: Personal smoke-free home action plan |  |  |
| Handout 5: Difficult situations and ‘if-then plans |  |  |
| Handout 6: Encouraging other household members to assist with creating a smoke-free home |  |  |
| Handout 7: Quitting smoking |  |  |

4. DEVELOPING A PERSONAL ACTION PLAN FOR REDUCING SHS LEVELS IN THE HOME

a. How did you find the process of developing a personal action plan to reduce levels of second-hand smoke in your home? 

	b. Did you think the plan you made was realistic, or not? Why? 

5. ASKING OTHER HOUSEHOLD MEMBERS TO ASSIST WITH CREATING A SMOKE-FREE HOME

a. Did you speak with XX (staff member) about persuading other household members to assist with creating a smoke-free home? (If no, go to section 6)

b. What was your experience of raising this issue with other household member like? (Did you feel prepared? Did you feel confident? Did household members listen? Were you able to get their help? Did you use any of the materials or graphs provided?) 

6. CHANGING BEHAVIOUR AS A RESULT OF AIR QUALITY FEEDBACK

a. How did you feel about the feedback you received from your first measure of air quality in your home?

b. Did you/others in your household make any changes to your/their smoking behaviour in the home as a result of your first set of personalised feedback?

If NO CHANGES were made: 

- Explore reasons for no change (i.e. was the personal action plan realistic, was there a lack of interest or motivation in changing, were they unable to negotiate change with others in the household, weather) 

- Explore barriers to (actual) change (i.e. these could focus on place, people, practical features of methodology. Are these barriers ones that were identified in the planning stages, or are they new barriers?)

- Are these barriers things that could be overcome – are they permanent barriers or ones that could be changed in the future? 

If YES, CHANGES were made:

- What changes did you make? (i.e. to own smoking behaviour in the home, and any changes to house rules on smoking for other members of family/friends) 

- How was change achieved? (i.e. what methods have you used, how was change negotiated with others)

- Did you make changes using your personal action plan? (if yes, how did this go? If not, what motivated change? Did taking part in the study play a role in changes made?)

7. MOST RECENT AIR QUALITY FEEDBACK 

a. How do you feel about your most recent air quality reading. Is it what you were expecting?   

b. Is this latest set of feedback likely to influence your/others smoking in the home? (i.e. making changes now that weren’t made earlier, keeping up the changes that have already been made, feeling de-motivated if levels have increased?)

c. Where do you think you might be with smoking in 6 months time? (explore any intentions to make changes to smoking behaviour, including cutting down, using e-cigarettes, and quitting)  

8. AIR QUALITY FEEDBACK 

- How understandable was the information you were given about your air quality measurements? (show them each feedback sheet in turn and ask for their opinions)

- Are there any ways that you think the information you were given could be improved? 

9. PERCEIVED BENEFITS OF TAKING PART IN THE STUDY

a. Have you learnt anything new about SHS as a result of taking part?	

b. Are there any benefits to having taken part in the study? (for the participant, for their family, or for their friends) Can you tell me about that?

10. PERCEIVED NEGATIVES OF TAKING PART IN THE STUDY:

- Have you had any negative experiences during the study, about any aspect of the research? (If so, do you think these can these be avoided in the future?)

11. CONCLUSIONS	
- Invite any other questions or comments

- Thank them once again for taking part and all their efforts

- Confirm that we will build on this learning as we take forward the indoor air quality monitoring work, and that we will share our findings in due course

- Ask if they would like to opt-in to receive a summary of findings 	

APPENDIX 10: TOPIC GUIDE FOR CARE PROFESSIONALS TAKING PART IN THE INTERVENTION PILOT 

Finding ways to reduce second-hand smoke exposure in homes:

The AFRESH programme

FOCUS GROUP TOPIC GUIDE

Introduction

- Thank workers for taking part in this research, run by the University of Aberdeen

- Outline purpose of focus group, which should take no longer than 1 hour

- Purpose: to try and find out more about your own experiences of involvement in this research, so that we can continue to refine our approach as we further develop this work

- Confirm all present consent to being audio recorded, restate that confidentiality will be maintained and no personally identifiable information will appear in publications or presentations 

- Invite workers to briefly introduce themselves and describe their work remit

Areas for discussion:

TRAINING 

1. To begin with, I’d like us to explore your experiences of the training you received before beginning to recruit parents to the study:

- In your opinion, did the training cover all areas required? For each area, did it go into the right amount of detail?

- Did the training give you the skills and knowledge required to provide second-hand smoke advice to parents confidently?

- Did the training give you the skills and knowledge required to work with the Dylos machines confidently?

- Do you have any comments or thoughts on how the training could be improved in the future? 

RECRUITMENT

2. Next I’d like us to focus on your experiences of recruiting parents to the study. How did you find the recruitment process?

- Was it easy/difficult to recruit parents? (and reasons)

- What factors did/do you take into consideration when judging who to approach to take part?

- Have there been any specific challenges/facilitators in recruiting to the study?

PROCOTOL

3. Next I’d like us to focus on your experiences of actually running the intervention with parents.

- Was ‘The AFRESH Programme’ manual helpful to you or not? (and reasons) Are there any specific parts you found particularly helpful/unhelpful?

- Were the handouts/leaflets helpful? Did you tend to use them with parents, or not?

- How feasible has it been to deliver the intervention in your work setting? 

- How could the intervention be improved?

DATA COLLECTION: AIR QUALITY MONITORS

4. Now I’d like us to think about the use of air quality monitors in the study. 

 

- Have there been any issues arising from participants using the air quality monitors?

 

- Have there been any issues with installing/removing air quality monitors?

- What has your experience been like downloading the air quality data?

- What has your experience been like providing parents with air quality feedback sheets? 

DATA COLLECTION: PARTICIPANT FEEDBACK

- Have you had any follow up discussions since the study ended with participants about their/others smoking behaviour in the home? 

FURTHER WORK 

6. How feasible do you think it would be to incorporate the use of air quality monitoring as part of routine practice within your work?

- What do you think the key challenges/facilitators would be? 

- If we were to run the study again, is there anything you can think of that could be improved or done differently? 

- Can you think of any other areas of community practice where air quality monitoring could potentially be incorporated in a similar way to here?

CONCLUSION

- Invite any other questions or comments from staff

- Thank them once again for taking part and all their efforts

- Confirm that we will build on this learning as we take forward the indoor air quality monitoring work, and that we will share our findings in due course 

[^1]: Royal College of Physicians Tobacco Advisory Group. ‘Passive Smoking and Children’. Royal College of Physicians, London, UK, 2010. 14. BBC News 25th March 2014. Campaign to cut children's smoke exposure http://www.bbc.co.uk/news/uk-scotland-scotland-politics-26732748 [Accessed 29th August 2014] 15. Semple S, et al. How Long Does Secondhand Smoke Remain in Household Air: Analysis of PM2.5 Data From Smokers' Homes. Nicotine Tob Res. 2014 Jun 5. pii: ntu089. [Epub ahead of print] 16. Scottish Government Public Health Information Campaign (2014). Find out how to protect your children from the hidden dangers of second-hand smoke. www.rightoutside.org [Accessed 9th September 2014]
[^2]: Gee I, et al. Nearly 85% of tobacco smoke is invisible--a confirmation of previous claims. Tob Control. 2013;22:429.
[^3]: Wilson I, et al. 'I'm not doing this for me': mothers' accounts of creating smoke-free homes. Health Educ Res. 2013 Feb;28:165-78.
[^4]: Jarvis MJ, et al.  Impact of smoke-free legislation on children's exposure to secondhand smoke: cotinine data from the Health Survey for England.  Tob Control 2012 Jan;21(1):18-23.
[^5]: Akhtar PC, et al. Changes in child exposure to environmental tobacco smoke (CHETS) study after implementation of smoke-free legislation in Scotland: national cross sectional survey. BMJ. 2007;335:545
[^6]: Edwards A, et al. Explaining risks: turning numerical data into meaningful pictures. BMJ. 2002 April 6; 324(7341): 827–830.
[^7]: Hawley ST, et al. The impact of the format of graphical presentation on health-related knowledge and treatment choices. Patient Educ Couns. 2008 Dec;73(3):448-55.
[^8]: McCaffery KJ, et al. Med Decis Making. 2012 Jul-Aug;32(4):532-44. The influence of graphic display format on the interpretations of quantitative risk information among adults with lower education and literacy: a randomized experimental study.
[^9]: Garcia-Retamero R, et al.  Using Visual Aids to Improve Communication of Risks about Health: A Review. Scientific World Journal. 2012; 2012: 562637.
[^10]: Rosen G, et al. A review of video exposure monitoring as an occupational hygiene tool.  Ann Occ Hyg 2005; 49: 201-217.
[^11]: Wilson I, et al. REFRESH-reducing families' exposure to secondhand smoke in the home: a feasibility study. Tob Control. 2013;22:e8. doi: 10.1136/tobaccocontrol-2011-050212.
[^12]: Department of Health. A smokefree future: a comprehensive tobacco control strategy for England. 2010. http://webarchive.nationalarchives.gov.uk/20130107105354/http://www.dh.gov.uk/en/Publications andstatistics/Publications/PublicationsPolicyAndGuidance/DH_111749  [Accessed 29th August 2014]
[^13]: Scottish Government. ‘Creating a Tobacco-Free Generation: A Tobacco Control Strategy for Scotland’. 2013  http://www.scotland.gov.uk/Resource/0041/00417331.pdf [Accessed 29th August 2014]
[^14]: BBC News 25th March 2014. Campaign to cut children's smoke exposure http://www.bbc.co.uk/news/uk-scotland-scotland-politics-26732748 [Accessed 29th August 2014]
[^15]: Semple S, et al. How Long Does Secondhand Smoke Remain in Household Air: Analysis of PM2.5 Data From Smokers' Homes. Nicotine Tob Res. 2014 Jun 5. pii: ntu089. [Epub ahead of print]
[^16]: Scottish Government Public Health Information Campaign (2014). Find out how to protect your children from the hidden dangers of second-hand smoke. www.rightoutside.org [Accessed 9th September 2014]
[^17]: de Bruin M et al.  Self-regulatory processes mediate the intention-behavior relation for adherence and exercise behaviors.  Health Psychol 2012; 31, 695-703.
[^18]: Michie S, et al. Effective techniques in healthy eating and physical activity interventions: a metaregression. Health Psychol. 2009; 28: 690-701.
[^19]: Deci E & Ryan RM.  The "what" and "why" of goal pursuits: Human needs and the selfdetermination of behaviour. Psychological Inquiry 2000; 11:227-268.
[^20]: Carver CS & Scheier MF. Control theory: A useful conceptual framework for personality-social, clinical, and health psychology. Psychological Bulletin 1982; 92: 111–135.
[^21]: Carver CS & Scheier MF. On the self-regulation of behavior. New York, NY: Cambridge University Press (1998).
[^22]: Semple S, et al. An inexpensive particle monitor for smoker behaviour modification in homes. Tob Control. 2013 Sep;22:295-8.
[^23]: Semple S, et al. Using a new, low-cost air quality sensor to quantify second-hand smoke (SHS) levels in homes. Tob Control. 2013 Sep 17. doi: 10.1136/tobaccocontrol-2013-051188
[^24]: Bartholomew LK, et al. 2011. Planning health promotion programs; an Intervention Mapping approach, 3rd Ed. San Francisco, CA: Jossey-Bass.
